# Allylic Carbocyclic Inhibitors Covalently Bind Glycoside Hydrolases

**DOI:** 10.1021/jacsau.3c00037

**Published:** 2023-03-20

**Authors:** Tatyana D. Grayfer, Khalil Yamani, Erik Jung, Gleb A. Chesnokov, Isabella Ferrara, Chien-Chi Hsiao, Antri Georgiou, Jeremy Michel, Aurélien Bailly, Simon Sieber, Leo Eberl, Karl Gademann

**Affiliations:** † Department of Chemistry, University of Zurich, Winterthurerstrasse 190, 8057 Zürich, Switzerland; ‡ Department of Plant and Microbial Biology, University of Zurich, Zollikerstrasse 107, 8008 Zürich, Switzerland

**Keywords:** total synthesis, natural products, carbohydrates, covalent inhibitors, proteomics

## Abstract

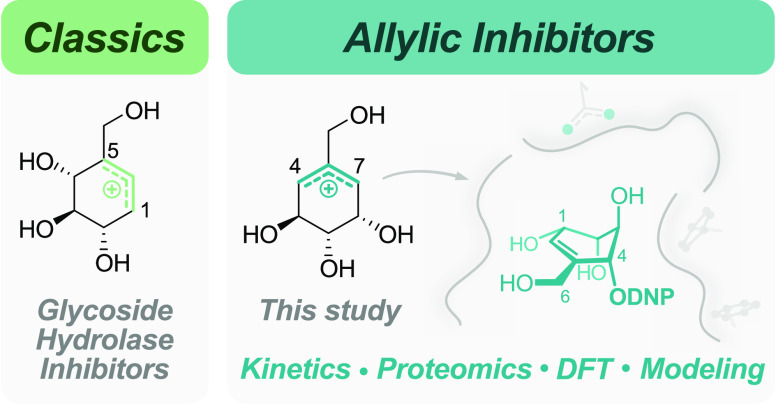

Allylic cyclitols were investigated as covalent inhibitors of glycoside hydrolases by chemical, enzymatic, proteomic, and computational methods. This approach was inspired by the C_7_ cyclitol natural product streptol glucoside, which features a potential carbohydrate leaving group in the 4-position (carbohydrate numbering). To test this hypothesis, carbocyclic inhibitors with leaving groups in the 4- and 6- positions were prepared. The results of enzyme kinetics analyses demonstrated that dinitrophenyl ethers covalently inhibit α-glucosidases of the GH13 family without reactivation. The labeled enzyme was studied by proteomics, and the active site residue Asp214 was identified as modified. Additionally, computational studies, including enzyme homology modeling and density functional theory (DFT) calculations, further delineate the electronic and structural requirements for activity. This study demonstrates that previously unexplored 4- and 6-positions can be exploited for successful inhibitor design.

Humans are fueled by a constant supply of glucose. Glycoside hydrolases (GHs) drive both glucose uptake and the digestion of oligosaccharides, glycoproteins, and glycolipids in the human body.^1−5^ Of medical relevance, α-glucosidases are responsible for the cleavage of polysaccharides, such as starch, and consequently, many inhibitors have been developed for use in the clinic,^6−11^ or for mechanistic studies.^6,12−15^ Therefore, the identification of new inhibitors drives the understanding of key mechanistic pathways in glycoscience.^1,16,17^

Streptol/valienol^18,19^ and kirkamide^20,21^ (Figure 1A) are C_7_ cyclitol natural products that have been identified^22,23^ in an obligatory symbiosis system composed of the plant *Psychotria kirkii* and the bacterium *Candidatus* Burkholderia kirkii. These natural products have been shown to act as allelochemicals inhibiting plant (competitor) growth,^19,23,24^ as insecticides,^20^ and grazer deterrents.^18^ An interesting compound is represented by streptol glucoside, which was isolated from the same bacteria/plant symbiosis. In this compound, glucose is linked *via* the glycosidic 1-position to the 4-position of streptol (Figure 1A). This compound raises an interesting question: can the allylic C–O bond be cleaved with glucose as leaving group? And more generally, can allylic cyclitols with leaving groups at both possible 4- and 6-positions function as glucoside hydrolase (GH) inhibitors (Figure 1B)?

**Figure 1:**
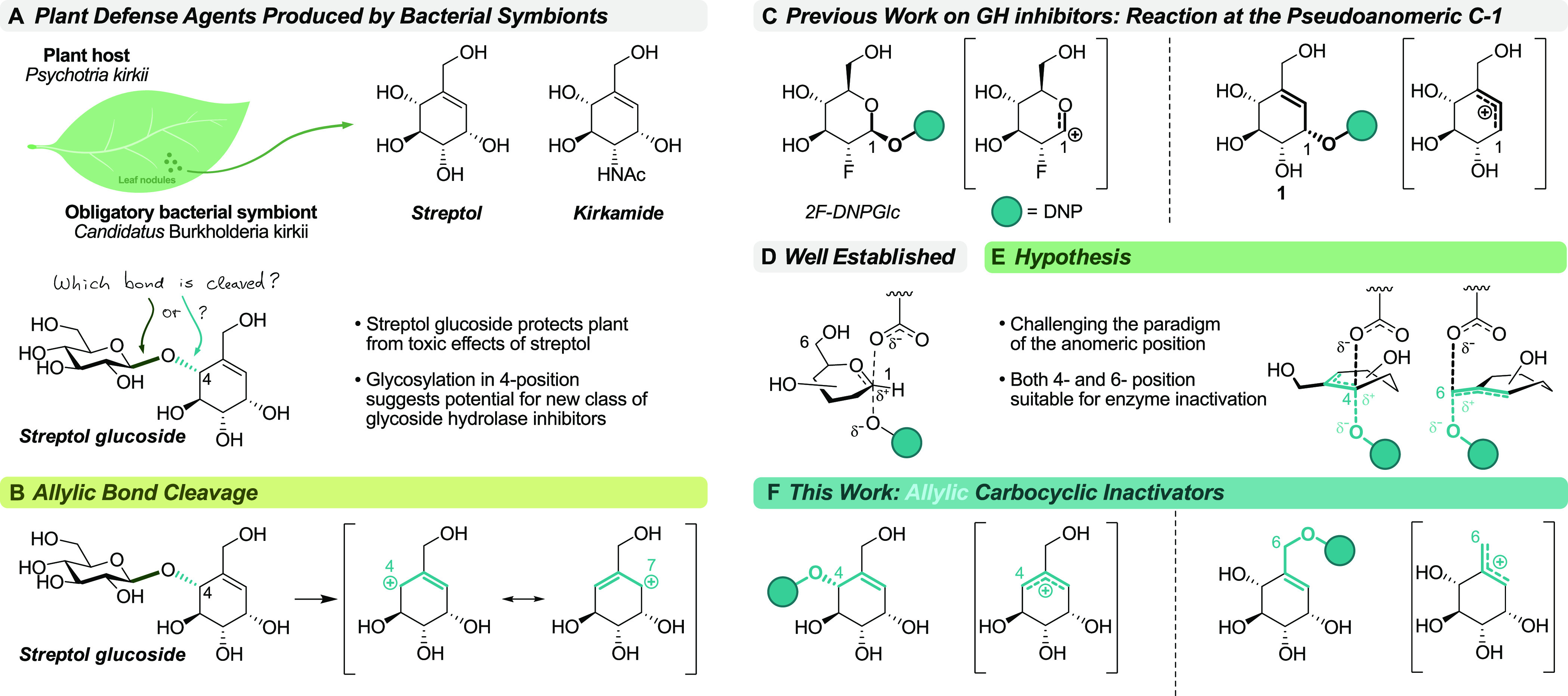
(A) Streptol (Valienol) and kirkamide are allelopathic agents produced by plant-symbiotic bacteria for defense purposes. Streptol glucoside features a potential leaving group in the 4-position.^18−24^ (B) Can the allylic C–O bond in streptol glucoside be cleaved? (C) Well-established mechanism for enzymatic carbohydrate hydrolysis at the anomeric position. (D) Can substrates with leaving groups at the 4- and 6-positions serve as inhibitors? In contrast to the well-established Koshland mechanism (C),^41^ the allylic inactivators might require reorientation in the active site (D). (E) Previous studies focused on the (pseudo)-anomeric 1-position.^25−40^ (F) This work investigates allylic carbocyclic inactivators. DNP = 2,4-dinitrophenyl.

The general mechanistic approach for GH inhibitors utilizes the pseudo-anomeric position of carbohydrate mimics for chemical modification, and built on this approach, mechanistic probes and clinical candidates have been developed for both noncovalent and covalent inhibitors.^8,25−29^ As a prototype example, 2,4-dinitrophenyl-2-deoxy-2-fluoro-α-d-glucopyranoside (2F-DNPGlc) utilizes the anomeric position for biological activity (Figure 1C, left).^30−33^ In an interesting extension, the groups of Bennet and Withers and their respective co-workers independently used a C_7_ cyclitol grafted with leaving groups in the pseudo-anomeric 1-position such as **1** (Figure 1C, right).^34−40^ They demonstrated that increasing the positive partial charges at position 1 promotes the formation of a covalent bond with the enzyme. However, all of these studies support the traditional Koshland mechanism with leaving groups in the C-1 position (Figure 1D).^41^

The central hypothesis in this study is that inhibitors with leaving groups at 4- and 6-position will interact with and ideally block GH enzymes (Figure 1E). To test this hypothesis, we prepared and evaluated the corresponding allylic carbocyclic inhibitors (Figure 1F). In this design, the leaving group and subsequent positive charge are localized in positions that are generally thought not to be accessible to GH enzymes. Consequently, as the hallmark Koshland mechanism invokes reaction at the C-1 position (Figure 1D),^17,41,42^ the proposed allylic inhibitors might require both rotational and conformational transposition in the active site (Figure 1E).^17,43−45^

In this study, we prepared and investigated streptol derivatives **2** and **3** featuring a 2,4-dinitrophenyl ether in positions 6 and 4, respectively. These compounds allowed us to probe the hypothesis whether intermediate cations at the 4- and 6-positions provide viable options for GH inhibitors. The inhibitory activity of the new inhibitors was evaluated by biochemical assays, and their mode of action was investigated by proteomics and computational approaches.

We first started with the preparation of known compounds **1** with a DNP ether^35^ and **1**′ with a *para*-nitrophenyl (PNP) ether^34^ for comparison purposes. The synthesis route is based on the known tricyclic compound **4**, prepared by Mehta’s method from benzoquinone and cyclopentadiene.^46^ The diol **4** was protected as an acetonide, followed by a *retro*-Diels–Alder reaction (Ph_2_O, 230 °C) to furnish a cyclohexanone, which was reduced by LiAlH_4_ to yield secondary alcohol **5** (48% over 3 steps) as a single diastereoisomer. The silyl group was cleaved under standard conditions, and the resulting 1,3-diol was transformed into an acetonide. Removal of the acetate in position 1 led to tricyclic bis-acetonide **6** (68% over 3 steps). The aryl group was then introduced via aromatic nucleophilic substitution with 1-fluoro-2,4-dinitrobenzene or 4-fluoronitrobenzene. After deprotection of the ketals with hydrochloric acid, the desired streptol derivatives **1** (88% over 2 steps) and **1**′ (63% over two steps) were obtained (Scheme 1).

**Scheme 1:**
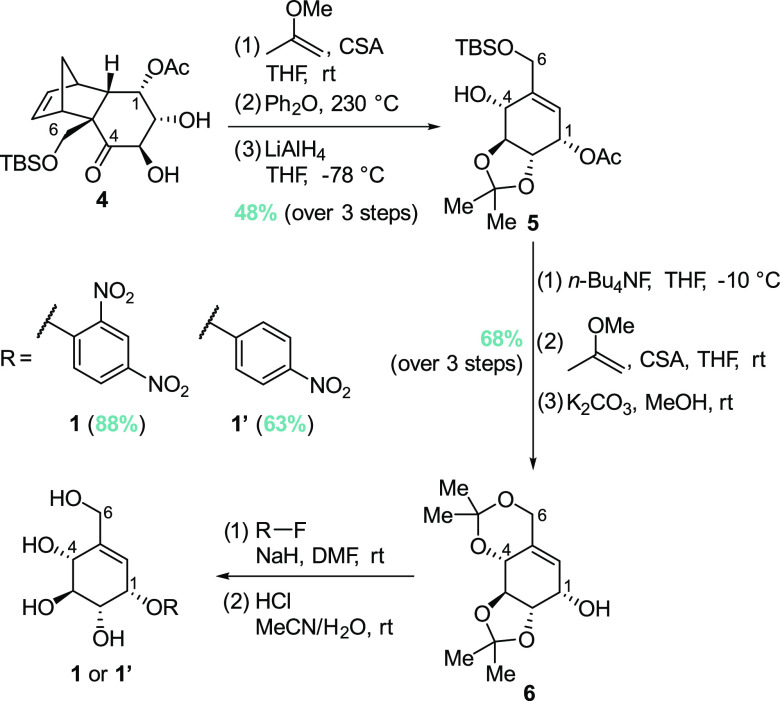
Synthesis of Streptol Derivatives **1** and **1**′ CSA = camphorsulfonic acid.

We then proceeded to the synthesis of targets **2** and **3** starting from alcohol **7**, which can be synthesized in three steps from **4**.^46^ Nucleophilic aromatic substitution of 1-fluoro-2,4-dinitrobenzene led to 2,4-dinitrophenyl ether **8** in an excellent yield of 93%. The first attempt to cleave the silyl ether group was achieved with *n*-Bu_4_NF (tetrahydrofuran, THF, 0 °C, 10 min). Following the removal of the silyl ether, we observed migration of 2,4-dinitrophenyl from C-4 to C-6. As the C-6 ether was one of our synthetic targets, we completed its synthesis through methanolysis of the acetate groups (NaOMe, MeOH) to 6-DNP streptol derivative **2**. Several conditions were evaluated to deprotect the silylated primary OH group in **8** without concomitant migration of the DNP group. Finally, the silyl ether was cleaved by exposure to aqueous hydrochloric acid in acetonitrile at room temperature. When complete desilylation was confirmed by thin-layer chromatography (TLC), dioxane and hydrochloric acid were added to the reaction mixture, which was heated to reflux resulting in global deprotection. This optimized protocol delivered the desired 4-DNP streptol derivative **3** in 38% yield (Scheme 2). It is important to note that carbasugar derivatives **2** and **3** are very hygroscopic compounds that also retain methanol and, consequently, must be dried under high vacuum for several days and stored under Ar to avoid contact with moisture. Their purity was assessed by quantitative NMR analysis (qNMR) prior to the experiments with the enzyme to evaluate their concentration as accurately as possible.

**Scheme 2:**
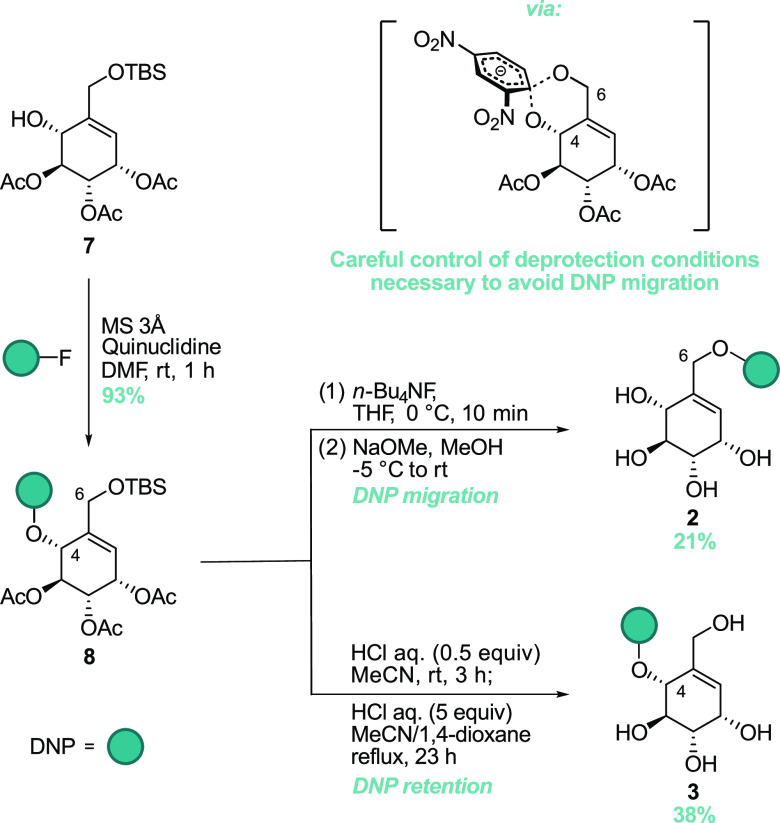
Synthesis of Streptol Derivatives **2** and **3** DNP = 2,4-dinitrophenyl.

We then evaluated the freshly synthesized compounds **2** and **3** for their inhibitory activity on GH13 yeast α-glucosidase from *Saccharomyces cerevisiae* incubated with the streptol derivatives at different concentrations and tested them against its known substrate 4-nitrophenyl-α-d-glucopyranoside (PNP-Glc) at different time points (see the Supporting Information, SI for the detailed protocol). Exponential decay of its activity was observed, providing strong evidence that compounds **2** and **3** indeed act as inhibitors of this α-glucosidase (Figure 2). The same inhibition assays were also performed with the inhibitors **1**, **1**′, **2**, **3**, and the GH13 yeast α-glucosidase G0660 (Figures S3 and S4). Noteworthy, by performing reactivation experiments, the activity of the enzyme was not restored even after 5 days except in the case of **1**′, as previously reported^35^ (Figures S5 and S6).

**Figure 2:**
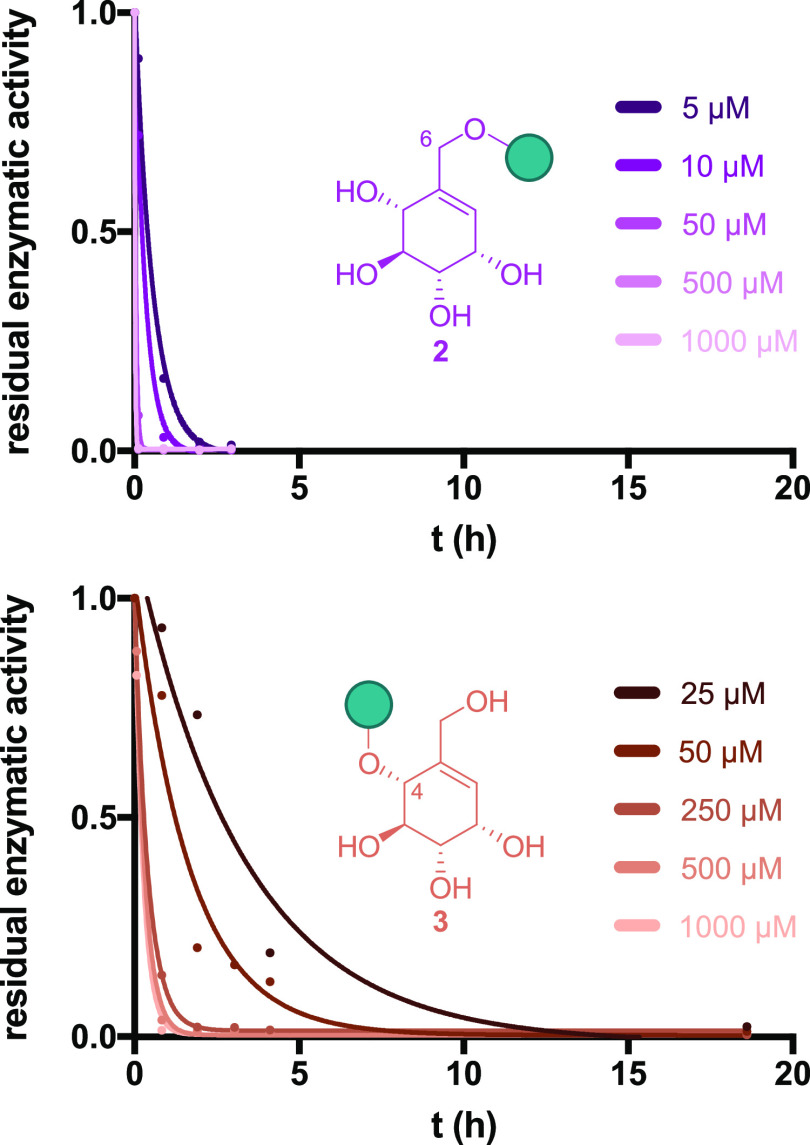
Residual enzymatic activity evolution over time of GH13 yeast α-glucosidase (G5003) incubated with **2**, purple curves on the top and **3**, brown curves on the bottom at different concentrations (see the SI for procedure).

Based on these observations, the kinetic model of α-glucosidase inactivation by a covalent inhibitor can be described by equation 1 in Scheme 3. The first step is the complexation of the inhibitor (I-LG) to the enzyme characterized by constant *K_i_*. The formation of the covalent bond is described by the first-order rate constant *k*_inact_ (Scheme 3).^35,47^

**Scheme 3:**
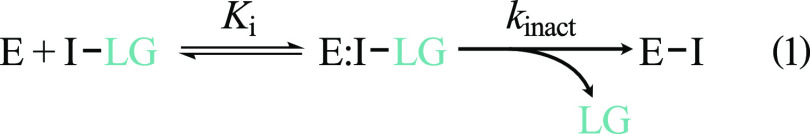
Kinetic Model of GH13 Yeast α-Glucosidase Inactivation by **2** and **3**

According to our hypothesis and this model, dinitrophenolate is released during the experiments. To investigate whether the released dinitrophenolate from the inhibitors interferes with the assay readout, we synthesized nonfluorescent difluorophenyl derivatives and conducted a series of control experiments. Compounds **9** and **9**′ possess a 2,6-difluorophenyl ether and a 3,5-difluorophenyl ether, respectively, on the 6-position of the streptol core. Both cyclitols were synthesized, and their activity against α-glucosidase G5003 was evaluated. Compound **7** was acetylated (NaOAc, Ac_2_O); then, the obtained silyl ether was reacted with buffered HF·py (preventing acetyl migration)^48^ to furnish the tetra-acetoxy derivative **10** in a good yield of 80% (2 steps). A Mitsunobu reaction was performed using 2,6-difluorophenol or 3,5-difluorophenol to yield the corresponding ethers **11** (61%) and **11**′ (93%). Methanolysis (K_2_CO_3_, MeOH) furnished the desired streptol derivatives functionalized in position 6 with a 2,6-difluorophenyl ether **9** (66%) or a 3,5-difluorophenyl ether **9**′ (47%) (Scheme 4).

**Scheme 4:**
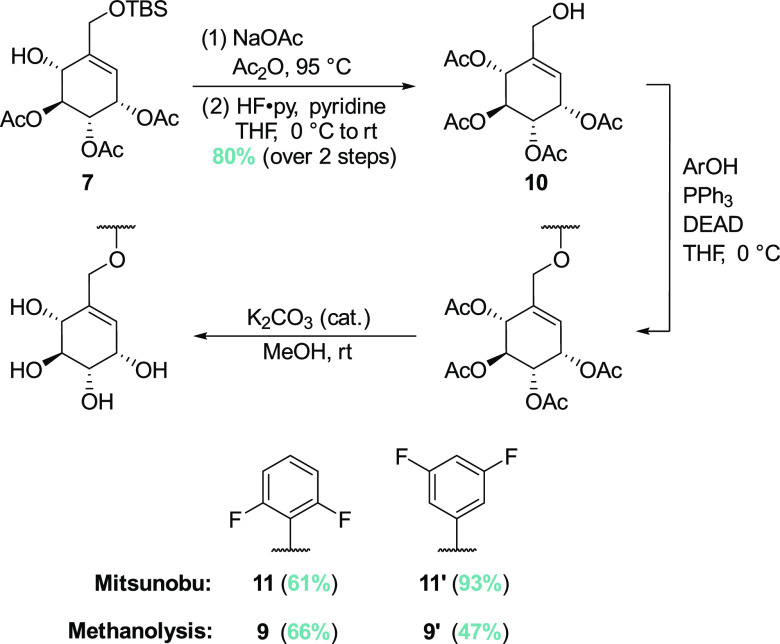
Synthesis of Streptol Derivatives **9** and **9**′

Based on previous findings and estimated from their higher p*K*_a_ values, these fluorinated compounds were expected to be significantly less active.^35^ The activity of these derivatives was evaluated. Derivative **9**′ is not soluble in water, even with addition of DMSO, preventing characterization of the inhibitory activity with the established enzyme assay. However, compound **9** could be solubilized in water, and no activity was observed up to a concentration of 2 mM. In addition, the compound solubility above 3 mM in water was too low to further evaluate its activity and therefore measure the kinetics parameters of inhibition of the enzyme by this compound (Figure 3). As predicted, these results underscore the importance of good leaving groups in the 6-position for inhibitory activity.

**Figure 3:**
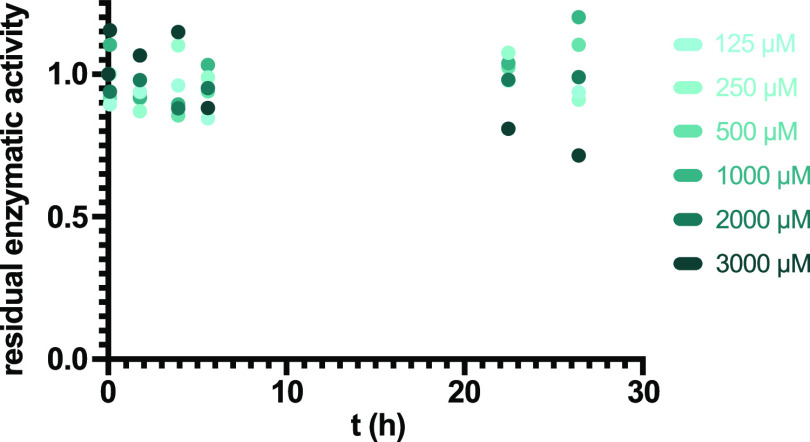
Residual enzymatic activity evolution over time of GH13 yeast α-glucosidase (G5003) incubated with **9** at the indicated concentrations (see the SI for procedure).

Released dinitrophenolate absorbs at 405 nm like *p*-nitrophenolate from PNP-Glc used to measure the activity of the enzyme (see the SI for procedure). To ensure that the observed increased absorbance in the assay was only due to PNP-Glc, the stability of the newly designed most-active inhibitor **2** was investigated. The absorbance of this compound in the media with or without PNP-Glc (Figure 4) was measured over time. As can be seen in Figure 4, up to inhibitor concentrations of 100 μM, there is no significant increase in absorption. It is only at inhibitor concentrations as high as 500 μM that the absorption caused by 2,4-dinitrophenolate becomes visible. However, in the assay performed for compounds **2** and **3**, the highest concentration used (in the “kinetic plate,” see procedure in the SI) was 100 μM. Therefore, compound **2** (and thus the less active **3**) is stable in the concentration range used in the assay. This control experiment allows us to neglect the absorbance due to the dinitrophenolate and indicates that these inhibitors have slow nonspecific reactivity with bovine serum albumin (BSA) that only becomes prominent at concentrations exceeding 100 μM.

**Figure 4:**
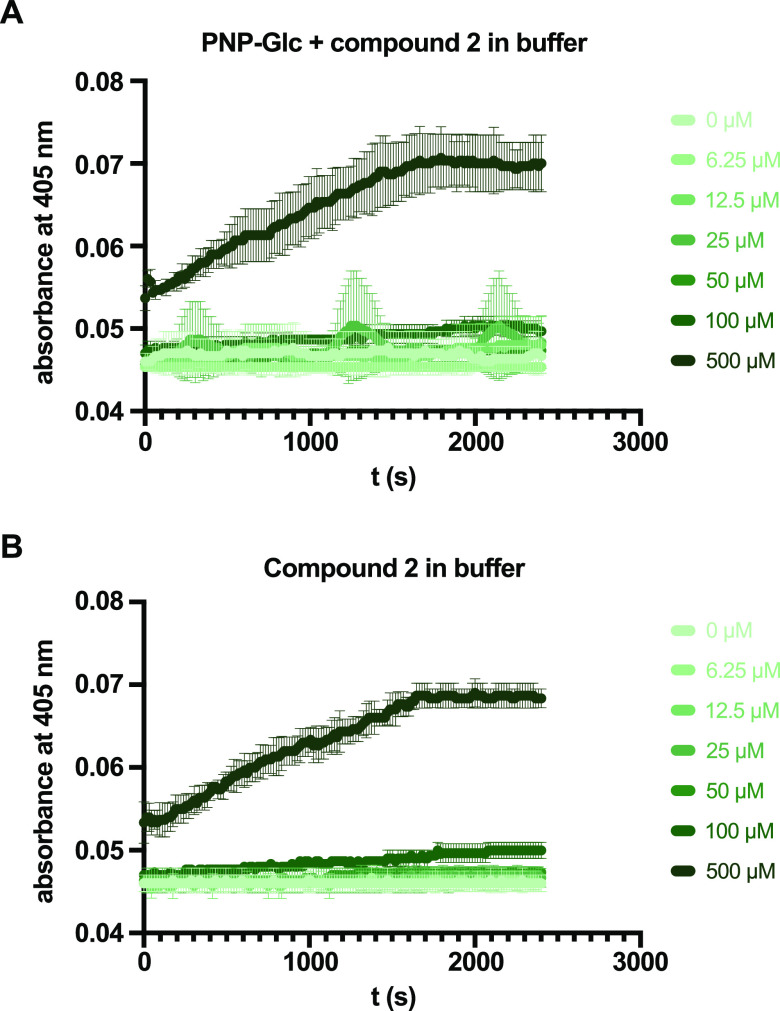
Serial dilution of compound **2** in milli-Q water (5, 1, 0.5, 0.25, 0.125, and 0.0625 mM) was prepared. (A) 10 μL of each concentration was then diluted 10-fold into 90 μL of a 50 μM solution of PNP-Glc in buffer (pH 6.83, BSA 1 mg/mL). (B) 10 μL of each concentration was then diluted 10-fold into buffer (pH 6.83, BSA 1 mg/mL). The absorbance at 405 nm was measured in intervals of 16 s for 40 min. The experiment was performed in triplicate. Data presented as mean ± standard deviation (SD) (*n* = 3).

Ensured of the accuracy of the inhibitory assay (Figure 2), the pseudo-first-order rate loss of activity of the enzyme was plotted *versus* inhibitor (**2** and **3**) concentration, and an excellent fit to the Michaelis–Menten equation was observed (Figure 5). Hence, *K_i_*, *k*_inact_, and the second-order rate constant *k*_inact_/*K_i_* were determined and are reported in Table 1, together with the data from Bennet’s studies.^35,36^ When the 2,4-dinitrophenyl ether substituent was transposed to the allylic C-6 position (compound **2**) instead of C-1 (compound **1**), the binding constants of the enzyme–inhibitor complex are surprisingly comparable (*K_i_*_,1_ ≈ *K_i_*_,2_). However, enzyme inactivation is slower for **2** and **3** than for **1**. The weakest inhibitory parameters were observed for PNP derivative **1**′, which is substituted by a worse leaving group than the other compounds (Table 1 and Figures S3 and S4). The inhibitory activity of the compounds can be ranked as follows: **1** ≫ **2** > **3** ≥ **1**′, for both enzyme G5003 and G0660 (Table 1 and Figures S3 and S4). Taken together, these data further corroborate the hypothesis that these streptol derivatives with leaving groups at the allylic 4- or 6-position act as α-glucosidase covalent inhibitors.

**Figure 5:**
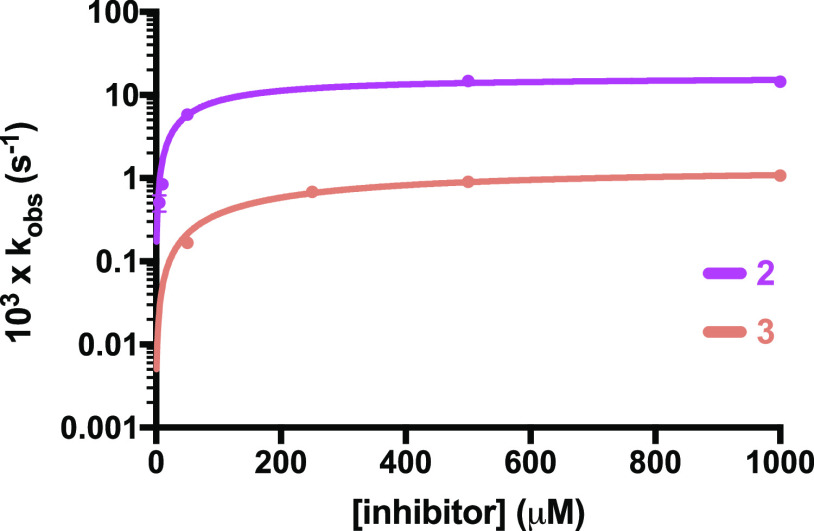
Kinetics for covalent inhibition of GH13 yeast α-glucosidase. Pseudo-first-order reaction rate *k*_obs_ (s^–1^) plotted against the concentration of inhibitor **2** (purple) and **3** (brown) with enzyme G5003. The lines are the best nonlinear fit to a standard Michaelis–Menten equation.

**Table 1:** Kinetic Parameters for the Covalent Inhibition of GH13 Yeast α-Glucosidase G5003 by Streptol Derivatives **1**, **1**′, **2**, and **3**

entry	compound	*k*_inact_ (×10^–3^ s^–1^)	*K_i_* (μM)	*k*_inact_/*K_i_* (M^–1^ s^–1^)
1	**1**	174 ± 28^a^	84 ± 19^a^	2070 ± 570^a^
2	**1**′	0.91 ± 0.06^a^	570 ± 90^a^	1.59 ± 0.27^a^
3	**2**	16.6 ± 0.9	96 ± 22	173 ± 49
4	**3**	1.41 ± 0.08	289 ± 47	4.87 ± 1.06

a Data taken from refs (35) and (36).

We next investigated the mechanism of action of these inactivators by mass spectrometry-based proteomics approaches. The mass spectra were measured for carbasugar derivatives **1**, **2**, and **3** incubated with enzymes G5003 and G0660 used for the assays and compared to that of the free enzyme. The mass spectra of the enzyme G5003 incubated with streptol derivatives **1**, **2**, and **3** display a shift (∼158 Da) compared to the spectrum of the free enzyme indicating the covalent addition of streptol (Figures 6 and S12–S16). Similar results were observed by comparing the mass spectrum of the free enzyme G0660 with that from the reaction product between the enzyme and **1**, **2**, and **3** (Figures S7–S11). In that case, the presence of lactose increases the complexity of the analysis due to the formation of several adducts with an increment of 324 Da (covalent addition of lactose). Taken together, these data provide evidence for one streptol unit covalently bound to this GH13 yeast α-glucosidase and further establish the 2,4-dinitrophenyl group as a competent leaving group on every allylic position (as in **1**, **2**, and **3**) of this carbasugar core.

**Figure 6:**
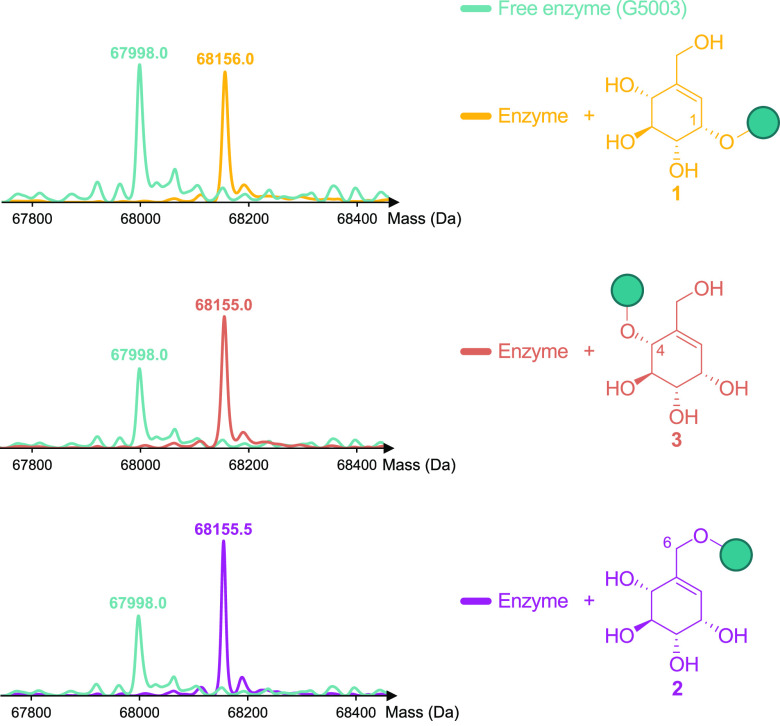
Mass spectrum of the free α-glucosidase G5003 in cyan and the product of the reaction of this enzyme incubated with the different inhibitors (**1** yellow, **3** brown, **2** purple).

To identify the site of labeling of the enzyme, trypsin-mediated digestion of α-glucosidase G5003 and G0660 incubated with **1**, **2**, or **3** (10 mM concentration) followed by LC-MS/MS analysis of the fragments was performed. For enzyme G5003, Asp214 (for **1** and **2**) and Asp5 (for **3**) are modified with a unit of 158.06 Da, as shown by the tandem MS/MS signal of the labeled peptide 213–220 and 3–12 (Figure S17). For the enzyme G0660, labeling of Asp214 is observed for all inhibitors; however, labeling of Asp5 is also observed for compounds **2** and **3** (Figures S18 and S19). Aspartic acid residues are indeed expected to be part of the active site of glucosidase, and Withers and co-workers reported evidence that the nucleophilic active site of the GH13 yeast α-glucosidase is Asp214.^49,50^ Based on all of these data, we conclude that the designed compounds are inhibiting the activity of the yeast α-glucosidase by blocking the nucleophilic residue in the active site. The labeling of **2** and **3** on Asp5 could be due to a nonspecific interaction, as Asp5 is solvent-exposed. Higher relative labeling of **2** and **3** compared to **1** may explain their lower inhibitory activity. To rationalize the difference observed in the enzyme inhibition depending on the position of the 2,4-dinitrophenyl ether substituent, we evaluated our inhibitors using two different computational approaches.

First, we studied the structure-based difference in the reactivity of compounds **1**, **2**, and **3** toward nucleophilic displacement of the DNPO-group. If the inhibition process is hypothesized to be on the S_N_1 side of the mechanistic continuum, we first compared the stability of the intermediate carbocations based on hydride affinity values (Figure 7). Hydride affinities were calculated as negatives of Gibbs energy differences of the corresponding hydride–ion addition reactions in implicit water (CPCM, as implemented in ORCA 5.0.1).^52^ The conformations of the carbocations lowest in energy were used, as found by CREST. The values for both direct and vinylogous addition of the hydride were calculated at the level of ωB97M-V/def2-TZVPP.^53,54^ The hydride affinity values for the allylic carbocations derived from **2** and **3**, and in particular the two ends of the allylic carbocations were found to be close: 105.0 and 104.0 kcal/mol for **3**, 106.1 and 104.6 kcal/mol for **2**. In contrast, the hydride affinity for the carbocation derived from **1** has a significantly lower value (94.8 kcal/mol), suggesting considerably higher stability. As a reference, the hydride affinity for the oxocarbenium ion derived from glucose was also calculated and found to be 90.1 kcal/mol. Therefore, the obtained values suggest **1** to be more prone to an S_N_1 type of reactivity than **2** or **3** thermodynamically, with **2** and **3** having similar stability of the corresponding allylic carbocations. Interestingly, the hydride affinity of **1cc** is closer to the one of glucose-derived carbocation than to the ones from **2** and **3**, which corroborates earlier studies.^34,38^ Such a difference in the stability of the carbocations can be attributed to the conformational properties of the intermediate carbocations. In **1cc**, there are two axial hydrogen atoms at C-2 and C-4 conformationally locked with the corresponding C–H bonds available for hyperconjugation; however, in carbocations **2cc** and **3cc**, there is only one axial C–H bond available for hyperconjugation in each case (H at C-4 for **2cc**, H at C-3 for **3cc**). Noteworthy, in the oxocarbenium ion from glucose, there is only one axial C–H bond available for hyperconjugation. These findings, coupled with Bell–Evans–Polanyi principle, suggest the following order of reactivity: **1** > **3** ≥ **2** in the case of S_N_1-type of reactions. The same reactivity pattern can also be expected in the case of S_N_2 type reactions, since in the transition state, the positive charge at the carbon center builds up, which means that energy barrier height should correlate with the stability of the corresponding carbocation (assuming similar steric demand). These results also suggest that inversion of the stereogenic center at C-1 in **2** or **3** would lead to more reactive compounds.

**Figure 7:**
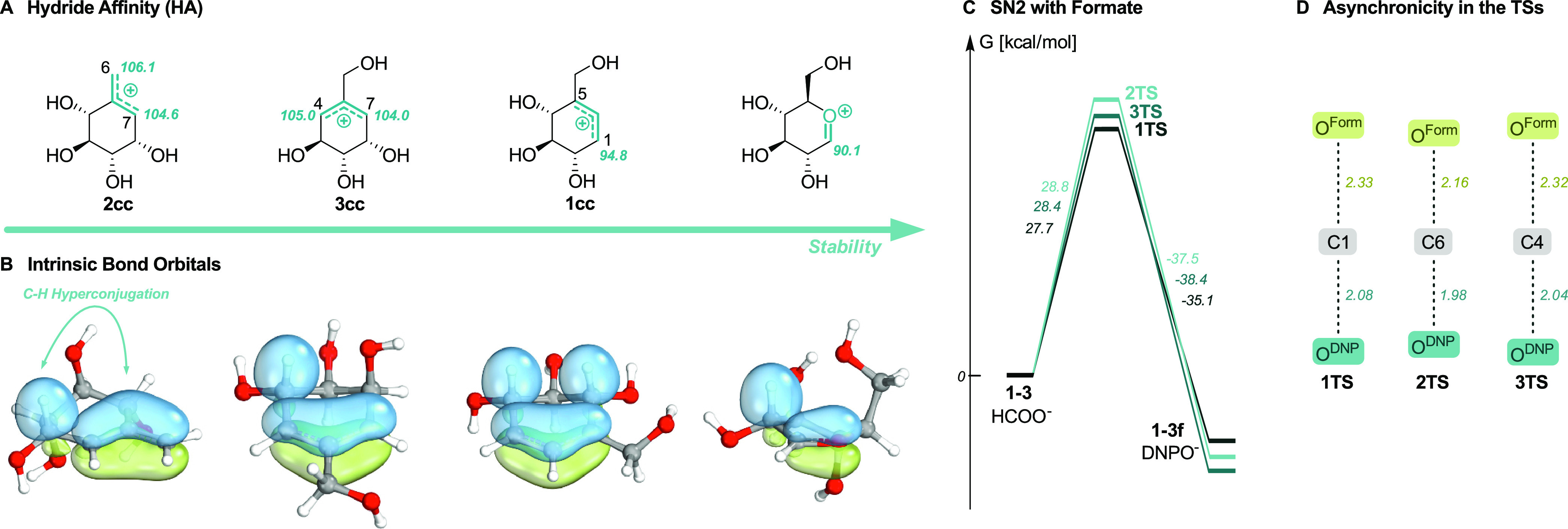
(A) Hydride affinity values for the carbocations derived from **1**, **2**, **3**, and glucose; (B) visualization of the Intrinsic Bond Orbitals (IBO)^51^ of the allylic system of the carbocations **1-3cc**, glycosyl pyranosylium cation, and C–H bonds involved in hyperconjugation; (C) energy profiles (Gibbs free energy, in kcal/mol) for the S_N_2 type reactions of **1**, **2**, and **3** with formate anion, CPCM(water)-ωB97M-V/def2-TZVPP//r^2^SCAN-3c; and (D) bond lengths (in Å) in the transition states showing the level of asynchronicity.

To investigate the S_N_2-type mechanistic hypothesis, we modeled S_N_2-type second-order displacement of the DNPO-group by a formate-ion as a model for the carboxylate in the enzyme pocket. It is important to mention that proper simulation of S_N_1 processes is still difficult due to the high importance of the dynamic influence of the solvent, especially in water.^55−57^ A recent study on *ab initio* molecular dynamics (AIMD) of glucosylation reactions^58^ showed the subtleties defining the position of the substitution type on the S_N_1/S_N_2 scale, especially emphasizing the role of the medium. The calculated barrier heights for the reactions were found to be in the same order as predicted by the thermodynamic analysis above based on hydride affinity values (**1** > **3** > **2**), with **1** being the most reactive toward substitution with formate-ion, with the barriers, however, being close in energy. An important property worth considering is the asynchronicity of the transition state. Since in both attacking and leaving species, the oxygen atoms are sp^2^-hybridized and negatively charged, a rather symmetrical transition state with roughly equal distances from the carbon center to the oxygen atoms of the corresponding groups would be expected, as DNPOH and HCOOH have similar p*K*_a_ values. However, in the case of compounds **1** and **3**, the distance difference was found to be ca. 0.3 Å, and in the case of **2**, this difference was slightly lower (0.18 Å), likely due to the primary nature of the position of the DNPO-group. These values rule out a pure S_N_2 mechanism, but to differentiate between S_N_1 and mixed S_N_1/S_N_2 mechanisms, a proper AIMD study is necessary. The non-S_N_2 nature of the substitution reactions allows the utilization of the hydride affinity values as a good qualitative measure for the reactivity of carbasugars. In the case of similar hydride affinity values, proper positioning in the enzymatic active site will impact the rate of the reaction. To computationally address this question, we used protein homology modeling with substrate binding studies.

We built our homology model using the crystal structure of isomaltase mutant E277A from *S. cerevisiae* in complex with isomaltose based on the literature (PDB: 3AXH).^59−61^ The pairwise identity between the sequence of these enzymes (72%, Figure S20 for sequence alignment) and the involvement of the same active site^59,60^ led us to use this structure for our homology model, as previously done by others.^62^ First, it is important to note that Asp5 in this model appears not to be involved in any enzymatic cavity (Figure S21). This observation supports our hypothesis that the labeling observed on Asp5 is due to a nonspecific interaction with the compounds, which is most likely not responsible for the inhibition. We constructed the structures of our compounds **1**, **2**, and **3** on the basis of the glucose fragment in the active site containing the catalytic residues Asp214 and Glu276 (Figure 8A). All structures were then manually posed and minimized using the MAB force field.^63^ What becomes immediately apparent is that due to the small size of the active site, the carbasugar fragment has to rotate to position the DNP moiety in the space normally occupied by the leaving saccharide. This rotation leads to different hydrogen-bonding networks between the respective derivatives, which impact significantly on binding affinity and reactivity. According to this model, compound **1** retains all interactions of glucose since its hydroxy groups are positioned in the same way (Figure 8B). To position the DNP moiety in its reactive axial orientation, compound **3** must undergo a ring-flip to its disfavored conformer with the OH groups on carbons 2 and 3 also in axial position. Still, **3** may form hydrogen bonds to multiple amino acid residues in the active site, including Asp214 (Figure 8C). An additional advantage of inhibitors based on functionalization of the nonstereogenic 6-position (such as compound **2**) is that any conformation of the cyclitol ring is potentially reactive since free rotation of the CH_2_ODNP group is possible. For inhibitor **2**, our model suggests that to engage in hydrogen bonds with Asp214, Arg212, Asp68, and Arg439, the DNP group possibly points into a different direction than for **1** and **3** (Figure 8A,D). The binding affinities of all derivatives are likely improved by donor–acceptor interactions of the DNP moieties with Phe157 or Phe300. A comparison of the three structures matches the experimentally observed trend in inhibitory activity (**1** ≫ **2** > **3**). Interestingly, these conformational and rotational differences in orientation in the active site raise further ramifications with regard to selectivity. On the one hand, different epimers could improve selectivity for this enzyme, or different glycosidases would yield different selectivity.

**Figure 8:**
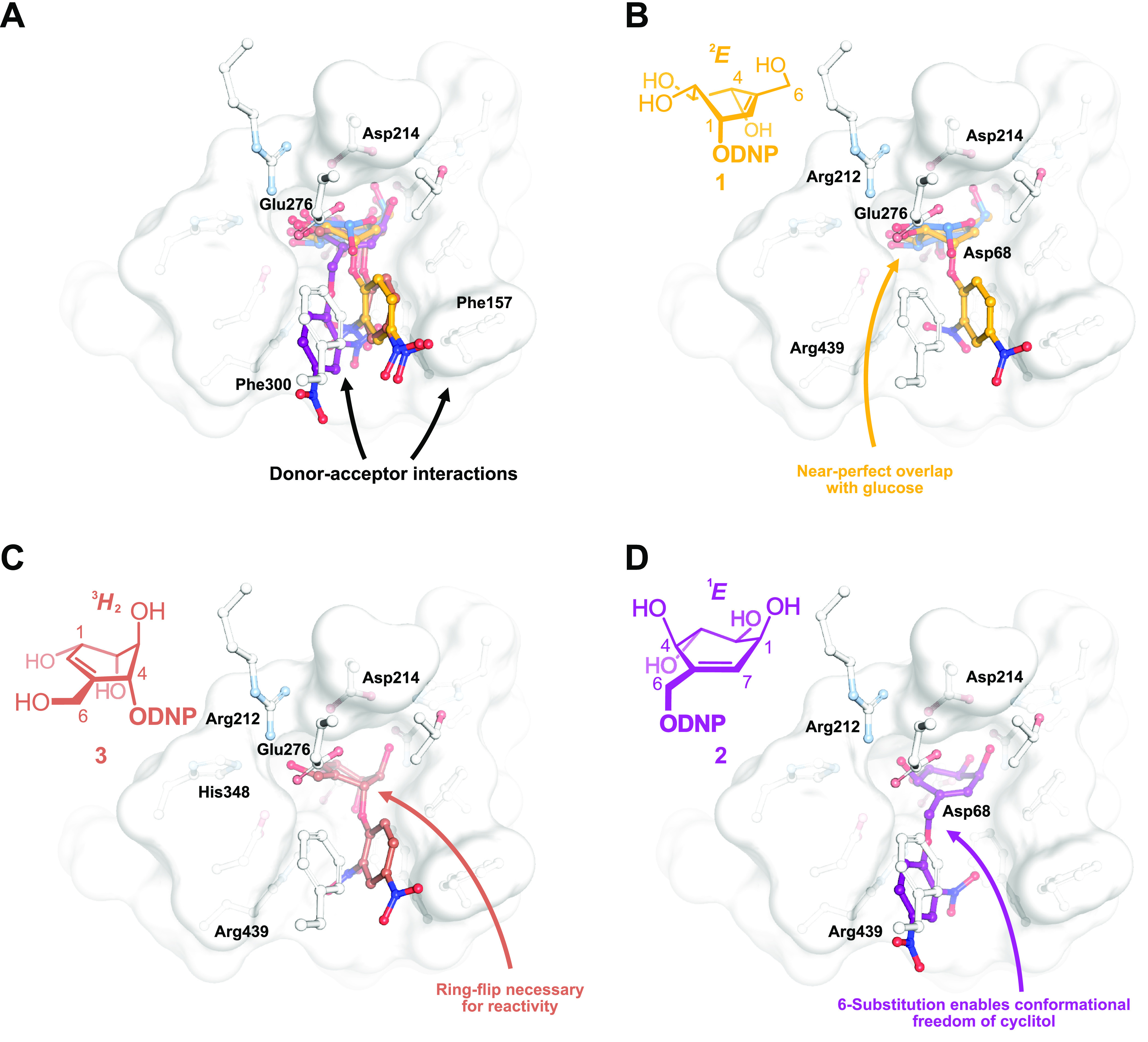
(A) Three-dimensional (3D) representation of minimized structures of glucose (blue), **1** (yellow), **2** (purple), and **3** (brown) in a homology model of maltase constructed from the gene MAL32 of *S. cerevisiae* built using a crystal structure of isomaltase from *S. cerevisiae* as a template. Only the residues and surfaces in the proximity of the active site are shown for clarity. (B) 3D representation of minimized structures of glucose (blue) and **1** (yellow). (C) 3D representation of minimized structures of glucose (blue) and **3** (brown). (D) 3D representation of minimized structures of glucose (blue) and **2** (purple).

In accordance with the work of Bennet,^34,35^ we propose the following mechanism for the inhibition of GH13 yeast α-glucosidase by compound **3**. The ^3^H_2_ conformation of **3** found in the inhibitor modeling study is consistent with a π −> σ*-orbital overlap necessary for the departure of the leaving group in the allylic position. Due to the increasing positive charge along the reaction coordinate, the transition structure **12** must involve five C-atoms in one plane such as E_2_ or ^3^E.^64,65^ Noteworthy, the E_2_ conformation was already observed recently in the X-ray structure of an inhibitor in complex with a GH.^66^ The enzyme-bound carbasugar **13** will then finally relax to a ^2^H_3_ conformation (Scheme 5, top).^67^ However, a nucleophilic attack at the C-7 position cannot be ruled out, for example, via an S_N_2′ reaction.

**Scheme 5:**
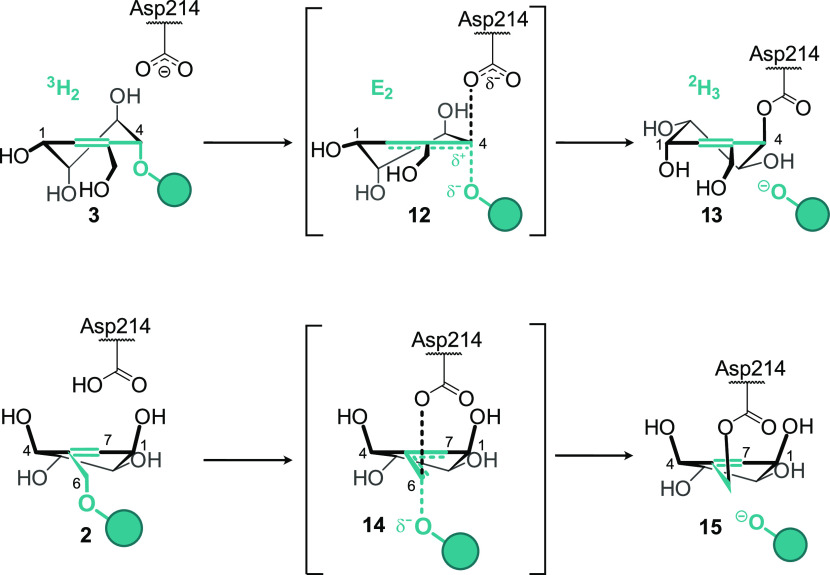
Postulated Mechanism for the Reaction of Streptol Derivative **3** (Top) and **2** (Bottom) with GH13 Yeast α-Glucosidase

Compound **2** features a leaving group in the 6-position not directly attached to the carbocyclic ring, and therefore, its enzymatic activation is hypothesized to be different to compounds **1** and **3**. Covalent enzyme inhibition could result from an attack at the 7- or 6-position. The availability of position 6, together with the computational data suggesting a more similar C–O_Asp_ and C–O_LG_ distance in the transition state, led us to hypothesize a labeling on the 6-position as being more likely than on the 7-position (Scheme 5, bottom).

Recently, the possibility of an epoxide as an intermediate in the cleavage of carbasugar by glycoside hydrolase of family 99 was reported.^68^ The formation of an epoxide in the case of compounds **1**, **2**, or **3** cannot be excluded at present, but no epoxide formation was observed by NMR analysis of these inhibitors dissolved in water after one week.

In summary, we synthesized and evaluated unusual allylic carbocyclic inhibitors of GH13 yeast α-glucosidase based on the natural product streptol glucoside. Leaving groups were installed on the 4- and 6-position of the carbasugar, in contrast to the usually employed C-1 (pseudo)-anomeric position. These novel allylic carbocycles inhibit the GH13 enzyme with kinetics comparable to known covalent inhibitors of GH. Covalent labeling of the catalytic aspartate residue, as evident from proteomics adds further evidence for these allylic carbocycles as covalent inhibitors of GH. Detailed computational studies provided mechanistic insight with regard to conformational requirements and subsequent reactions on the S_N_1/S_N_2 continuum. Further studies will investigate if natural products such as streptol glucoside and kirkamide will exploit this unusual mechanism of GH inactivation. In addition, this work might stimulate studies to overcome the classically used 1-position for covalent inhibitors.

## Supporting Information Available

Experimental procedures, physical characterization, proteomics data, sequence alignment, details to computational studies, and NMR spectra (PDF)
