# Supplementary material for: Allylic Carbocyclic Inhibitors Covalently Bind Glycoside Hydrolases
Source: JACS Au. 2023 Mar 20;3(4):1151–61. doi: 10.1021/jacsau.3c00037 (PMC10131216; doi:10.1021/jacsau.3c00037)

Supplementary Information

Allylic Carbocyclic Inhibitors Covalently Bind Glycoside Hydrolases

Authors: Dr. Tatyana D. Grayfer, Dr. Khalil Yamani, Erik Jung, Gleb A. Chesnokov, Isabella Ferrara, Dr. Chien-Chi Hsiao, Dr. Antri Georgiou, Jeremy Michel, Dr. Aurélien Bailly, Dr. Simon Sieber, Prof. Dr. Leo Eberl, Prof. Dr. Karl Gademann

Table of Contents

GENERAL EXPERIMENTAL METHODS: CHEMISTRY 3

EXPERIMENTAL PROCEDURES AND ANALYTICAL DATA 4

GENERAL EXPERIMENTAL METHODS: BIOLOGY 19

BIOLOGICAL ASSESSMENT OF THE INHIBITORS 19

STUDY OF ENZYME-INHIBITOR ADDUCTS BY MASS-SPECTROMETRY 23

SITE MAPPING OF INHIBITOR ALKYLATION ON THE α-GLUCOSIDASE FROM *SACCHAROMYCES CEREVISIAE*. 29

SEQUENCE ALIGNMENT 34

MODELING AND OPTIMIZATION OF INHIBITORS USING MOLOC 35

COMPUTATIONAL DETAILS FOR THE DFT CALCULATIONS 35

REFERENCES 37

General experimental methods: chemistry

All chemicals were purchased from Acros, Fluka, TCI, Fisher or Sigma-Aldrich and were used without further purification. All reactions were carried out in heat gun-dried glassware (unless aqueous reagents were used) and reactions involving air sensitive compounds were performed under an Ar atmosphere. Solvents used for large-scale chemical transformations were either puriss. quality or HPLC grade. Small-scale reactions were carried out using dry solvents purchased from Acros. Synthetic transformations were monitored by TLC, ^{1}H–NMR spectroscopy and/or UHPLC. Yields refer to purified, dried, and spectroscopically pure compounds, unless stated otherwise. TLC were performed on Merck silica gel 60 F_{254} aluminum plates. Stains were visualized under a UV lamp (254 nm) and with a 4-anisaldehyde stain in ethanol. Concentration under reduced pressure was performed at 20-40°C using a rotary evaporator. Flash chromatography was performed using silica gel 60 (230-400 mesh) from Sigma-Aldrich. Proton (^{1}H) and carbon (^{13}C) NMR spectra were recorded on Bruker Avance spectrometers (300 MHz, 400 MHz, and 500 MHz) at room temperature. The following abbreviations were used for the description of the protocol: CSA: Camphorsulfonic acid. Carbon NMR (^{13}C) spectra were recorded at 126 or 101 MHz. NMR experiments were carried out in the following solvents: deuterochloroform ($CDCl_3$), chemical shifts (δ) are reported in parts per million (ppm) with reference to $CDCl_3$ (^{1}H: 7.26; ^{13}C: 77.16); deuteromethanol (CD_3OD), chemical shifts (δ) ppm with reference to CD_3OD (^{1}H: 3.34; ^{13}C: 49.86); heavy water (D_2O), chemical shifts (δ) are reported in parts per million (ppm) with reference to D_2O (^{1}H: 4.79); deuterated dimethylsulfoxide [$(CD_3)_2SO$], chemical shifts (δ) are reported in parts per million (ppm) with reference to $(CD_3)_2SO$ (^{1}H: 2.50; ^{13}C: 39.52). The following abbreviations are used for the proton spectra multiplicities: s: singlet, bs: broad singlet, d: doublet, t: triplet, q: quartet, m: multiplet, br: broad. Coupling constants (*J*) are reported in Hertz (Hz). Atom numbering is provided in order to facilitate attribution of the NMR peaks but does not necessarily follow nomenclature rules. IR spectra were recorded on a Perkin Elmer Spectrum Two ATR-FTIR. The absorptions are reported in cm^{-1}. Mass spectra (HRMS-ESI) were recorded by the Mass Spectrometric Service of the University of Zürich on a QExactive instrument (Thermo Fisher Scientific, Bremen, Germany) equipped with a heated electrospray (ESI) ionization source and connected to a Dionex Ultimate 3000 UHPLC system. Optical rotations $°[\alpha]_D^T$ were measured at the sodium D line using a 1 mL cell with a 1 dm or 0.1 dm path length on a Jasco P-2000 digital polarimeter. The concentration *c* is given in g/100 mL of solvent.

Experimental procedures and analytical data

(1*S*,4*R*,4a*S*,5*S*,6*R*,7*R*,8a*R*)-8a-(((*tert*-butyldimethylsilyl)oxy)methyl)-6,7-dihydroxy-8-oxo-1,4,4a,5,6,7,8,8a-octahydro-1,4-methanonaphthalen-5-yl acetate (4).

This compound was prepared according to the reported procedure. [1]

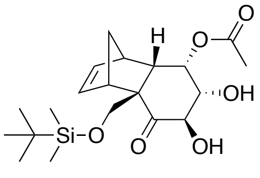

4

$C_{20}H_{32}O_6Si$
M = 396.56 g/mol

NMR ^{1}H (500 MHz, CDCl$_3$) δ 6.27 (dd, J = 5.6, 2.9 Hz, 1H), 5.97 (dd, J = 5.7, 3.2 Hz, 1H), 5.77 (dd, J = 9.3, 4.1 Hz, 1H), 4.28 (d, J = 9.0 Hz, 1H), 4.05 (dd, J = 12.0, 4.1 Hz, 1H), 3.81 (d, J = 12.0 Hz, 1H), 3.33 (d, J = 9.0 Hz, 1H), 3.01 (s, 1H), 2.92 (dd, J = 9.3, 3.2 Hz, 1H), 2.79 (s, 1H), 2.17 (s, 3H), 1.53 (d, J = 8.8 Hz, 1H), 1.45 (dt, J = 8.9, 1.7 Hz, 1H), 0.85 (s, 9H), 0.03 (s, 3H), -0.00 (s, 3H).

NMR ^{13}C (126 MHz, CDCl$_3$) δ 214.1, 170.7, 139.9, 133.4, 75.0, 72.4, 69.5, 69.4, 62.4, 50.7, 48.5, 46.8, 46.6, 26.0 (3CH$_3$), 21.4, 18.4, -5.4, -5.5.

Spectral data matched with those from literature.[1]

(3a*S*,4*S*,4a*S*,5*R*,8*S*,8a*R*,9a*R*)-8a-(((tert-butyldimethylsilyl)oxy)methyl)-2,2-dimethyl-9-oxo-3a,4,4a,5,8,8a,9,9a-octahydro-5,8-methanonaphtho[2,3-d] [1,3]dioxol-4-yl acetate (16).

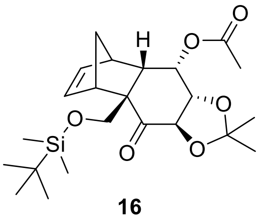

16

$C_{23}H_{36}O_6Si$
M = 436.62 g/mol

To a solution of **4** (2.50 g, 6.30 mmol, 1.0 eq.) in THF (40 ml) were added CSA (29 mg, 0.126 mmol, 0.02 eq.) and 2-methoxypropene (1.05 mL, 10.8 mmol, 1.71 eq.) at rt. After stirring for 1 h, an additional portion of 2-methoxypropene (0.35 mL, 3.60 mmol, 0.57 eq.) was added, followed by CSA (10 mg, 0.043 mmol, 0.007 eq.). After 0.5 h, the reaction mixture was diluted with EtOAc and water was added. The layers were separated, and the aqueous layer was extracted with EtOAc. The combined organic layers were washed with water twice, dried over MgSO$_4$, filtered, and concentrated under reduced pressure. The residue was purified by flash

chromatography on silica gel (pentane/EtOAc gradient, 95:5 to 85:15) to afford acetonide **16** (2.44 g, 89%) as a white powder.

MP = 140 °C.

NMR ^{1}H (500 MHz, $CDCl_3$) δ 6.34 (dd, *J* = 5.6, 2.9 Hz, 1H), 6.06 (dd, *J* = 5.7, 3.2 Hz, 1H), 5.85 (dd, *J* = 8.8, 3.7 Hz, 1H), 4.40–4.31 (m, 2H), 4.08 (d, *J* = 11.8 Hz, 1H), 3.29 (d, *J* = 9.1 Hz, 1H), 3.00 (bs, 1H), 2.92 (dd, *J* = 8.9, 3.1 Hz, 1H), 2.77 (bs, 1H), 2.15 (s, 3H), 1.55 (bd, *J* = 8.9 Hz, 1H), 1.49 (dt, *J* = 8.9, 1.9 Hz, 1H), 1.49 (s, 3H), 1.35 (s, 3H), 0.86 (s, 9H), 0.04 (s, 3H), 0.02 (s, 3H).

NMR ^{13}C (126 MHz, $CDCl_3$) δ 207, 170.2, 139.9, 133.9, 113.1, 79.2, 74.8, 71.7, 66.7, 66.0, 50.4, 49.2, 48.9, 46.4, 27.1, 26.9, 26.0 ($3CH_3$), 21.4, 18.4, -5.4, -5.5.

HR-ESI-MS calcd. for $C_{23}H_{36}O_6SiNa$ [*M*+Na^+]: 459.21734. Found: 459.21754.

IR (neat, cm^{-1}): 2927, 2855, 1728, 1458, 1377, 1340, 1252, 1226, 1170, 1137, 1115, 1092, 1071, 1030, 1001, 956, 916, 863, 835, 815, 776, 759, 719, 695, 623, 504.

$[\alpha]_D^{26}$ = +0.2 (*c* 0.215, $CHCl_3$).

(3a*S*,4*S*,7a*R*)-6-(((tert-butyldimethylsilyl)oxy)methyl)-2,2-dimethyl-7-oxo-3a,4,7,7a-tetrahydrobenzo[d][1,3]dioxol-4-yl acetate (17).

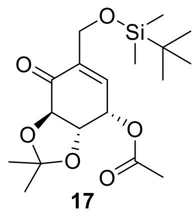

17

$C_{18}H_{30}O_6Si$
M = 370.52 g/mol

A solution of **16** (4.55 g, 10.4 mmol) in Ph_2O (70 mL) was heated to 230 °C for 2 h. After cooling to rt, the solvent was partially distilled off under reduced pressure at ca. 120 °C (0.1 mbar). The residue was purified by flash chromatography on silica gel (pentane/EtOAc gradient, 100:0 to 90:10) to afford α,β-unsaturated ketone **17** (2.65 g, 69%) as a pale-yellow viscous oil.

NMR ^{1}H (500 MHz, $CDCl_3$) δ 6.81 (dt, *J* = 5.6, 2.2 Hz, 1H), 5.91 (m, 1H), 4.69 (d, *J* = 10.6 Hz, 1H), 4.50 (ddd, *J* = 16.6, 2.3, 1.5 Hz, 1H), 4.31 (dd, *J* = 16.6, 2.2 Hz, 1H), 4.00 (dd, *J* = 10.5, 3.5 Hz, 1H), 2.14 (s, 3H), 1.49 (s, 3H), 1.46 (s, 3H), 0.90 (s, 9H), 0.07 (s, 6H).

NMR ^{13}C (126 MHz, $CDCl_3$) δ 192.6, 170.1, 142.9, 133.5, 112.9, 76.8, 75.9, 64.9, 59.7, 26.7, 26.6, 26.0 ($3CH_3$), 20.9, 18.4, -5.34, -5.36.

HR-ESI-MS calcd. for $C_{18}H_{30}O_6SiNa$ [*M*+Na^+]: 393.17039. Found: 393.17060.

IR (neat, cm^{-1}): 2932, 2858, 1749, 1715, 1463, 1374, 1221, 1184, 1152, 1132, 1082, 1044, 1008, 974, 941, 920, 898, 835, 777, 731, 671, 507.

$[\alpha]_D^{26}$ = +161 (*c* 0.112, $CHCl_3$).

(3a*S*,4*S*,7*R*,7aS)-6-(((tert-butyldimethylsilyl)oxy)methyl)-7-hydroxy-2,2-dimethyl-3a,4,7,7a-tetrahydrobenzo[d][1,3]dioxol-4-yl acetate (5).

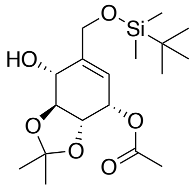

5

$C_{18}H_{32}O_6Si$

M = 372.53 g/mol

To a solution of **17** (350 mg, 0.945 mmol, 1.0 eq.) in dry THF (10 mL) was added $LiAlH_4$ (54 mg, 1.42 mmol, 1.5 eq.) at -78 °C. After stirring for 1.5 h at this temperature, $LiAlH_4$ (10 mg, 0.26 mmol, 0.28 eq.) was added and the stirring was continued for 2.5 h. Then, the reaction mixture was diluted with EtOAc and hydrolyzed with a saturated aqueous solution of NH_4Cl while warming to rt. The aqueous layer was extracted with EtOAc, the organic layers were combined, washed with brine, dried over $MgSO_4$, filtered and concentrated under reduced pressure. The crude residue was purified by flash chromatography on silica gel (CH_2Cl_2/EtOAc gradient, 100:0 to 90:10) to afford alcohol **5** (276 mg, 78%) as a white powder.

MP = 89 °C.

NMR ^{1}H (500 MHz, $CDCl_3$) δ 5.81–5.78 (m, 1H), 5.59 (app. t, *J* = 4.6 Hz, 1H), 4.48–4.37 (m, 2H), 4.32 (d, *J* = 13.9 Hz, 1H), 4.03 (app. t, *J* = 9.2 Hz, 1H), 3.58 (dd, *J* = 10.0, 3.6 Hz, 1H), 2.98 (d, *J* = 4.0 Hz, 1H), 2.08 (s, 3H), 1.48 (s, 3H), 1.45 (s, 3H), 0.91 (s, 9H), 0.09 (s, 6H).

NMR ^{13}C (126 MHz, $CDCl_3$) δ 170.5, 145.8, 119.0, 111.7, 77.4, 77.2, 75.2, 72.2, 64.2, 27.2, 26.6, 26.0 (3CH_3), 21.1, 18.4, -5.26, -5.30.

HR-ESI-MS calcd. for $C_{18}H_{32}O_6SiNa^+$ [*M*+Na^+]: 395.18604. Found: 395.18603.

IR (neat, cm^{-1}): 3464, 2986, 2955, 2930, 2857, 1744, 1472, 1463, 1372, 1229, 1171, 1135, 1085, 1067, 1045, 1008, 942, 836, 815, 776, 748, 673, 510.

$[\alpha]_D^{25}$ = +121 (*c* 0.22, $CHCl_3$).

(3aS,4S,9aR,9bS)-2,2,8,8-tetramethyl-3a,4,9a,9b-tetrahydro-6H-[1,3]dioxolo[4',5':5,6]benzo[1,2-d][1,3]dioxin-4-ol (6).

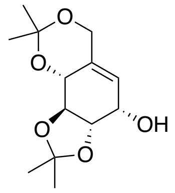

6
$C_{13}H_{20}O_5$
M = 256.30 g/mol

To a solution of **5** (500 mg, 1.34 mmol, 1.0 eq.) in dry THF (14 mL) at 0 °C was added TBAF (1M solution in THF, 1.5 mL, 1.5 mmol, 1.1 eq.). After stirring for 10 min, the solvent was evaporated, and the residue was dissolved in EtOAc. The resulting solution was passed through a pad of silica gel (EtOAc) and concentrated under reduced pressure to afford the desired crude diol**.** The latter (339 mg, 1.3 mmol, 1 eq.) was dissolved in THF (19 mL). 2-methoxypropene (0.38 mL, 3.9 mmol, 3 eq.) and CSA (30 mg, 0.13 mmol, 0.1 eq.) were added to the reaction mixture at rt. After stirring for 20 min, the mixture was diluted with EtOAc and water, the layers were separated, and the aqueous layer was further extracted with EtOAc. The combined organic layers were washed with water then brine, dried over $MgSO_4$, filtered, and concentrated under reduced pressure to afford the diacetonide (quantitative). This compound (393 mg, 1.3 mmol, 1.0 eq.) was dissolved in MeOH (20 mL) and K_2CO_3 (180 mg, 1.3 mmol, 1.0 eq.) was added at rt. After stirring for 20 min, the reaction mixture was diluted with water and EtOAc, the layers were separated, and the aqueous layer was extracted with EtOAc. The combined organic layers were washed with water then with brine, dried over $MgSO_4$, filtered, and concentrated under reduced pressure. The crude residue was purified by flash chromatography on silica gel (pentane/EtOAc gradient, 90:10 to 50:50) to afford alcohol **6** (225 mg, 68%) as a white solid.

MP = 108 °C.

NMR ^{1}H (500 MHz, $CDCl_3$) δ 5.62–5.64 (m, 1H), 4.56–4.49 (m, 2H), 4.45 (bd, *J* = 14.3 Hz, 1H), 4.25 (bd, *J* = 14.3 Hz, 1H), 4.06 (dd, *J* = 9.9, 8.2 Hz, 1H), 3.52 (dd, *J* = 9.9, 3.7 Hz, 1H), 2.26 (s, 1H), 1.55 (s, 3H), 1.49 (s, 3H), 1.49 (s, 3H), 1.42 (s, 3H).

NMR ^{13}C (126 MHz, $CDCl_3$) δ 137.3, 120.2, 111.9, 99.4, 77.0, 74.2, 71.4, 64.4, 62.6, 27.5, 27.3, 26.8, 20.9.

HR-ESI-MS calcd. for $C_{13}H_{20}O_5Na^+$ [M+Na^+]: 279.12029. Found: 279.12030.

IR (neat, cm^{-1}): 3445, 2991, 2927, 2887, 1386, 1373, 1248, 1222, 1201, 1164, 1129, 1102, 1087, 1071, 1034, 1008, 990, 975, 951, 890, 841, 794, 787, 747, 699, 667, 529, 506.

$[\alpha]_D^{25}$ = +60 (*c* 0.215, $CHCl_3$).

(3aS,4S,9aR,9bS)-4-(2,4-dinitrophenoxy)-2,2,8,8-tetramethyl-3a,4,9a,9b-tetrahydro-6H-[1,3]dioxolo[4',5':5,6]benzo[1,2-d][1,3]dioxine (18).

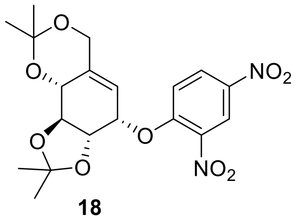

$C_{19}H_{22}N_2O_9$
M = 422.39 g/mol

To a solution of **6** (30 mg, 0.117 mmol, 1.0 eq.) in dry DMF (1.2 mL) were added sequentially 2,4-dinitrofluorobenzene (DNFB) (22 µL, 0.176 mmol, 1.5 eq.) and NaH (60% suspension in mineral oil, 7.0 mg, 0.176 mmol, 1.5 mmol) at rt. After stirring for 4.5 h, DNFB (10 µL, 0.080 mmol, 0.68 eq.) and NaH (2.0 mg, 0.050 mmol, 0.43 eq.) were added again and the stirring was pursued overnight. Additional DNFB (10 µL, 0.080 mmol, 0.68 eq.) and NaH (4 mg, 0.10 mmol, 0.86 eq.) were added and the reaction mixture was stirred for 9 h. It was further diluted with EtOAc and hydrolyzed with a saturated aqueous solution of NH_4Cl. The aqueous layer was extracted with EtOAc, the combined organic layers were washed with water ten times and once with brine, dried over $MgSO_4$, filtered, and concentrated under reduced pressure. The crude residue was purified by flash chromatography on silica gel (pure CH_2Cl_2) to afford ether **18** (45 mg, 91%) as a pale-yellow oil.

NMR ^{1}H (400 MHz, $CDCl_3$) δ 8.70 (d, *J* = 2.8 Hz, 1H), 8.40 (dd, *J* = 9.4, 2.8, Hz, 1H), 7.41 (dd, *J* = 9.4 Hz, 1H), 5.69–5.67 (m, 1H), 5.32 (app. t, *J* = 4.0Hz, 1H), 4.57 (d, *J* = 8.4 Hz, 1H), 4.48 (d, *J* = 14.9 Hz, 1H), 4.36–4.22 (m, 2H), 3.77 (dd, *J* = 10, 3.4 Hz, 1H), 1.57 (s, 3H), 1.46 (s, 6H), 1.33 (s, 3H).

NMR ^{13}C (101 MHz, $CDCl_3$) δ 156.4, 141.9, 140.4, 139.5, 128.6, 121.8, 116.6, 115.1, 112.7, 99.9, 76.0, 74.5, 72.9, 71.1, 62.3, 27.3, 27.1, 26.3, 21.1.

HR-ESI-MS calcd. for $C_{19}H_{23}O_9N_2^+$ [M+H^+]: 423.13981. Found: 423.13994.

IR (neat, cm^{-1}): 2990, 2924, 2855, 1604, 1526, 1486, 1373, 1342, 1274, 1223, 1199, 1156, 1086, 1068, 1044, 1012, 975, 945, 932, 899, 872, 833, 789, 774, 742, 723, 675, 644, 526.

$[\alpha]_D^{25}$ = +77 (*c* 0.45, $CHCl_3$).

(1R,2S,3R,6S)-6-(2,4-dinitrophenoxy)-4-(hydroxymethyl)cyclohex-4-ene-1,2,3-triol (1).

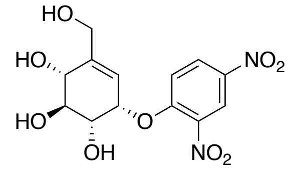

1

$C_{13}H_{14}N_2O_9$
M = 342.26 g/mol

To a solution of **18** (30 mg, 0.071 mmol, 1.0 eq.) in MeCN (1 mL) was added hydrochloric acid (1M aq., 0.03 mL, 0.03 mmol, 0.4 eq.). After stirring for 15 min, the solvent was evaporated. The crude residue was purified by flash chromatography on silica gel (CH_2Cl_2/MeOH gradient, 100:0 to 90:10 followed by EtOAc/MeOH 90:10) to afford an oil, filtered through an SPE column to afford **1** (23.5 mg, 97%) as a solid.

NMR ^{1}H (500 MHz, DMSO) δ 8.69 (d, *J* = 2.9 Hz, 1H), 8.44 (dd, *J* = 9.4, 2.9 Hz, 1H), 7.81 (d, *J* = 9.5 Hz, 1H), 5.86–5.82 (m, 1H), 5.36 (app. t, *J* = 4.5 Hz, 1H), 5.21 (d, *J* = 6.3 Hz, 1H), 5.07 (dd, *J* = 7.7, 4.7 Hz, 2H), 4.84 (app. t, *J* = 5.5 Hz, 1H), 4.13 (dd, *J* = 16.0, 5.5 Hz, 1H), 3.95 (dd, *J* = 16.0, 5.7 Hz, 1H), 3.81 (app. t, *J* = 6.9 Hz, 1H), 3.65 (ddd, *J* = 12.0, 7.5, 4.6 Hz, 1H), 3.55 (dt, *J* = 9.7, 4.2 Hz, 1H).

NMR ^{13}C (126 MHz, DMSO) δ 156.7, 149.2, 139.0, 139.0, 128. 7, 121.1, 117.2, 114.0, 76.5, 72.5, 72.4, 70.4, 60.5.

HR-ESI-MS calcd. for $C_{13}H_{14}N_2O_9Na^+$ [*M*+Na^+]: 365.05915. Found: 365.05915.

IR (neat, cm^{-1}): 3326, 1606, 1527, 1485, 1346, 1313, 1278, 1101, 1068, 1045, 1025, 989, 929, 835, 743.

$[\alpha]_D^{25}$ = +177 (*c* 0.145, MeOH)

(3aS,4S,9aR,9bS)-2,2,8,8-tetramethyl-4-(4-nitrophenoxy)-3a,4,9a,9b-tetrahydro-6H-[1,3]dioxolo[4',5':5,6]benzo[1,2-d][1,3]dioxine (19).

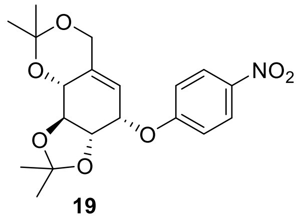

19

$C_{19}H_{23}NO_7$
M =: 377.39 g/mol

To a solution of **6** (20.5 mg, 0.080 mmol, 1.0 eq.) in dry DMF (1 mL) was added NaH (60% suspension in mineral oil, 4.8 mg, 0.12 mmol, 1.5 eq.) at 0 °C. After stirring for 10 min, 4-fluoronitrobenzene (13 µL, 0.12 mmol, 1.5 eq.) was added at this temperature and the reaction mixture was stirred for 25 min. The reaction mixture was hydrolyzed with a saturated aqueous

solution of NH_4Cl and diluted with EtOAc. The aqueous layer was extracted with EtOAc and the combined organic layers were washed with brine five times, dried over $MgSO_4$, filtered, and concentrated under reduced pressure. The residue was purified by flash chromatography on silica gel (pentane/EtOAc gradient, 95:5 to 80:20) to afford ether **19** (30 mg, 99%) as a pale-yellow solid.

NMR ^{1}H (400 MHz, $CDCl_3$) δ = 8.16 (d, *J* = 9.3 Hz, 2H), 7.06 (d, *J* = 9.3 Hz, 2H), 5.67 (d, *J* = 4.4 Hz, 1H), 5.16 (app. t, *J* = 4.2 Hz, 1H), 4.57 (d, *J* = 8.3 Hz, 1H), 4.49 (d, *J* = 14.5, 1H), 4.39–4.19 (m, 2H), 3.74 (dd, *J* = 10.0, 3.4 Hz, 1H), 1.58 (s, 3H), 1.45 (s, 6H), 1.30 (s, 3H).

NMR ^{13}C (101 MHz, $CDCl_3$) δ = 163.9, 139.1, 133.2, 125.8 (2C), 117.0, 116.0 (2C), 112.3, 99.6, 76.4, 74.7, 71.3, 71.0, 62.5, 27.5, 27.2, 26.4, 20.7.

HR-ESI-MS calcd. for $C_{19}H_{23}NO_7Na^+$ [*M*+Na^+]: 400.13667. Found: 400.13675

IR (neat, cm^{-1}): 2989, 2927, 2855, 1592, 1513, 1494, 1455, 1374, 1341, 1298, 1252, 1224, 1200, 1172, 1089, 1050, 1015, 946, 930, 902, 847, 791, 752, 704, 690, 523.

$[\alpha]_D^{24}$ = +67 (*c* 0.091, $CHCl_3$).

(1*R*,2*S*,3*R*,6*S*)-4-(hydroxymethyl)-6-(4-nitrophenoxy)cyclohex-4-ene-1,2,3-triol (1’).

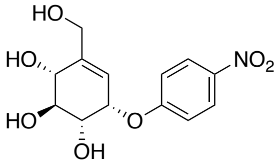

1’
$C_{13}H_{15}NO_7$
M = 297.26 g/mol

To a solution of **19** (32 mg, 0.085 mmol, 1.0 eq.) in MeCN (1 mL) was added hydrochloric acid (1M aq., 0.5 mL, 0.5 mmol, 5.9 eq.). After 10 min stirring, the solvent was evaporated. The crude residue was purified by flash chromatography on silica gel (EtOAc/MeOH gradient, 100:0 to 90:10) to afford **1’** (16 mg, 64%) as a pale-beige solid.

NMR ^{1}H (400 MHz, MeOD) δ 8.23–8.13 (m, 2H), 7.22–7.13 (m, 2H), 6.03 (app. dq, *J* = 5.2, 1.8 Hz, 1H), 5.11 (app. t, *J* = 4.6 Hz, 1H), 4.23 (d, *J* = 15.8 Hz, 1H), 4.18 (d, *J* = 15.8 Hz, 1H), 4.05–3.96 (m, 2H), 3.75 (dd, *J* = 10.0, 3.9 Hz, 1H).

NMR ^{13}C (101 MHz, MeOD) δ 165.3, 147.7, 142.7, 126.7 (2CH), 118.0, 116.8 (2CH), 75.2, 74.5, 74.1, 72.0, 62.7.

Spectral data matched those from literature.[2]

HR-ESI-MS calcd. for $C_{13}H_{15}NO_7Na^+$ [*M*+Na^+]: 320.07407. Found: 320.07382.

IR (neat, cm^{-1}): 3361, 2928, 1590, 1496, 1340, 1257, 1087, 1046, 991, 910, 888, 854, 753, 728, 694, 598.

$[\alpha]_D^{24}$ = +250 (*c* 0.8, MeOH).

(1*S*,2*S*,3*S*,6*R*)-5-(((*tert*-butyldimethylsilyl)oxy)methyl)-6-hydroxycyclohex-4-ene-1,2,3-triyl triacetate (7). Prepared according to reported procedure. [1]

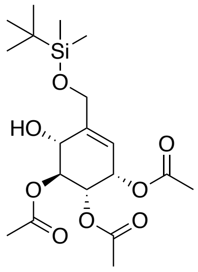

7
$C_{19}H_{32}O_8Si$
M = 416.54 g/mol

NMR ^{1}H (400 MHz, CDCl$_3$) δ 5.78 (app. dd, *J* = 5.6, 1.6 Hz, 1H), 5.58 (app. t, *J* = 4.7 Hz, 1H), 5.37 (dd, *J* = 10.8, 7.1 Hz, 1H), 5.10 (dd, *J* = 10.8, 3.8 Hz, 1H), 4.36–4.27 (m, 3H), 3.11 (bs, *J* = 4.6 Hz, 1H), 2.12 (s, 3H), 2.08 (s, 3H), 2.02 (s, 3H), 0.90 (s, 9H), 0.08 (s, 6H).
^{13}C NMR (101 MHz, CDCl$_3$) δ 171.5, 170.5, 170.1, 144.6, 117.7, 73.4, 71.8, 68.7, 66.2, 63.6, 26.0 3CH$_3$, 21.1 2xCH$_3$, 20.8, 18.4, -5.30, -5.32.
Spectral data matched with those from literature.[1]

(1*S*,2*S*,3*S*,6*R*)-5-(((*tert*-butyldimethylsilyl)oxy)methyl)-6-(2,4-dinitrophenoxy) cyclohex-4-ene-1,2,3-triyl triacetate (8).

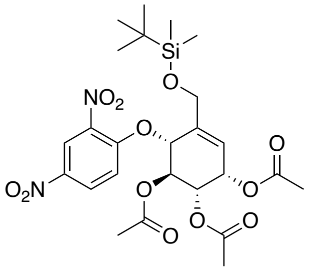

8
$C_{25}H_{34}N_2O_{12}Si$
M = 582.63 g/mol

To a solution of **7** (96 mg, 0.23 mmol, 1.0 eq.) in dry DMF (1 mL) were added successively 3Å molecular sieves (100 mg), 2,4-dinitrofluorobenzene (116 µL, 0.92 mmol, 4.0 eq.) and quinuclidine (211 mg, 1.9 mmol, 8.0 eq.). After stirring for 2 h, the reaction mixture was diluted with EtOAc and hydrolyzed with a saturated aqueous solution of NH_4Cl, the layers were separated and the aqueous layer was extracted with EtOAc. The combined organic layers were washed with water six times then with brine, dried over $MgSO_4$, filtered and concentrated under vacuum. The crude residue was purified by flash chromatography on silica gel (CH_2Cl_2/pentane gradient, 50:50 to 100:0, then CH_2Cl_2/EtOAc gradient, 100:0 to 95:5) to afford **8** (126 mg, 93%) as a pale-yellow solid.
MP = 65 °C.

NMR ^{1}H (400 MHz, $CDCl_3$) δ 8.67 (d, J = 2.8 Hz, 1H), 8.40 (dd, J = 9.3, 2.8 Hz, 1H), 7.62 (d, J = 9.5 Hz, 1H), 5.93 (dd, J = 4.6, 1.7 Hz, 1H), 5.71–5.61 (m, 2H), 5.32–5.22 (m, 2H), 4.35 (d, J = 12.9 Hz, 1H), 4.13 (d, J = 13.0 Hz, 1H), 2.14 (s, 3H), 2.03 (s, 3H), 1.99 (s, 3H), 0.78 (s, 9H), -0.01 (s, 3H), -0.11 (s, 3H).

NMR ^{13}C (101 MHz, $CDCl_3$) δ 170.4, 170.2, 169.4, 156.0, 140.8, 139.9, 139.6, 128.5, 122.6, 121.7, 115.9, 76.4, 69.5, 67.5, 65.7, 63.1, 25.8 ($3CH_3$), 21.2, 20.8, 20.7, 18.3, -5.4, -5.5.

HR-ESI-MS calcd. for $C_{25}H_{38}O_{12}N_3Si^+$ [M+NH_4^+]: 600.22193. Found: 600.22233.

IR (neat, cm^{-1}): 2927, 2855, 1745, 1605, 1531, 1471, 1367, 1344, 1282, 1238, 1214, 1150, 1067, 1044, 1008, 977, 940, 924, 904, 834, 779, 743, 719, 701, 672, 599.

$[\alpha]_D^{26}$ = +11 (c 0.071, $CHCl_3$).

(1*S*,2*S*,3*S*,4*R*)-5-((2,4-dinitrophenoxy)methyl)cyclohex-5-ene-1,2,3,4-tetraol (2).

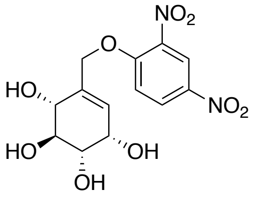

2

$C_{13}H_{14}N_2O_9$

M = 342.26 g/ mol

To a solution of **8** (420 mg, 0.721 mmol, 1.0 eq.) in dry THF (20 mL) at 0 °C was added *n*-Bu_4NF (1M in THF, 0.80 mL, 0.80 mmol, 1.1 eq.). After stirring for 10 min, the reaction mixture was diluted with EtOAc and hydrolyzed with a saturated aqueous solution of $NaHCO_3$. The layers were separated, and the aqueous phase was extracted with EtOAc. The combined organic layers were washed with brine, dried over $MgSO_4$, filtered, and concentrated under vacuum to afford the corresponding triacetate as a yellow oil. The resulting triacetate (337 mg, 0.72 mmol, 1 eq.) was diluted in MeOH (10 mL) and sodium methoxide (57.5 mg, 1.06 mmol, 1.50 eq.) was added at rt. After 1.5 h stirring, the reaction mixture was diluted with EtOAc and Amberlyst-15® was added. The mixture was then filtered through celite (MeOH) and concentrated under vacuum. Purification by flash chromatography on silica gel (CH_2Cl_2/MeOH gradient, 98:2 to 90:10) afforded **2** (52 mg, 21% over two steps) as a yellow solid.

Decomposition observed above 140°C.

NMR ^{1}H (400 MHz, MeOD) δ 8.75 (d, J = 2.8 Hz, 1H), 8.49 (dd, J = 9.3, 2.8 Hz, 1H), 7.52 (d, J = 9.3 Hz, 1H), 6.02 (dd, J = 5.1, 1.6 Hz, 1H), 5.03 (d, J = 12.6 Hz, 1H), 4.91 (d, J = 12.6 Hz, 1H), 4.25 (app. t, J = 4.6 Hz, 1H), 4.09 (d, J = 6.8 Hz, 1H), 3.78 (dd, J = 10.0, 7.2 Hz, 1H), 3.52 (dd, J = 10.0, 4.2 Hz, 1H).

NMR ^{13}C (101 MHz, MeOD) δ 157.6, 141.6, 140.4, 138.3, 130.2, 126.8, 122.5, 116.6, 74.1, 73.3, 72.4, 71.2, 67.5.

HR-ESI-MS calcd. for $C_{13}H_{14}N_2O_9Na^+$ [*M*+Na$^+$]: 365.05915. Found: 365.05920.

IR (neat, cm^{-1}): 3356, 2926, 1604, 1522, 1457, 1346, 1314, 1279, 1162, 1127, 1108, 1092, 1063, 1011, 983, 926, 915, 893, 830, 743, 705, 648, 615.

$[\alpha]_D^{25}$ = +38 (*c* 0.12, MeOH).

(1*R*,2*S*,3*S*,6*R*)-6-(2,4-dinitrophenoxy)-5-(hydroxymethyl)cyclohex-4-ene-1,2,3-triol (3).

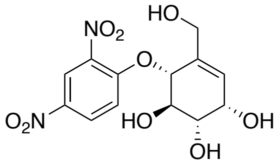

3

$C_{13}H_{14}N_2O_9$

M = 342.26 g/mol

To a solution of silyl ether **8** (472 mg, 0.772 mmol, 1 eq.) in acetonitrile (20 mL) was added aqueous hydrochloric acid (0.25 M aq., 3.4 mL, 0.72 mmol, 1 eq.) a rt. After stirring for 1 h, complete conversion of the starting material was assessed by TLC (Pentane/EtOAc: 60/40), and then dioxane (7 mL) and hydrochloric acid (1M aq., 2.5 mL, 2.5 mmol, 3.2 eq.) were added and the solution was warmed to reflux. After stirring for 23 h, the reaction mixture was brought to rt, diluted with EtOAc and hydrolyzed with a saturated aqueous solution of $NaHCO_3$. The phases were separated, and the aqueous phase was extracted with EtOAc. The combined organic layers were washed with brine, dried over Na_2SO_4, filtered, and concentrated under vacuum. Purification by flash chromatography on silica gel (CH_2Cl_2/MeOH gradient, 98:2 to 90:10) afforded **3** (79 mg, 30%) as a yellow solid.

Decomposition observed above 140°C.

NMR ^{1}H (400 MHz, MeOD) δ 8.69 (d, *J* = 2.8 Hz, 1H), 8.45 (dd, *J* = 9.4, 2.9 Hz, 1H), 7.87 (d, *J* = 9.5 Hz, 1H), 6.03 (app. dd, *J* = 5.3, 1.6 Hz, 1H), 5.39 (d, *J* = 7.4 Hz, 1H), 4.38 (app. t, *J* = 4.8 Hz, 1H), 4.22 (d, *J* = 13.5 Hz), 4.15–4.08 (m, 2H), 3.73 (dd, *J* = 10.3, 4.2 Hz, 1H).

NMR ^{13}C (101 MHz, MeOD) δ 158.4, 141.5, 141.2, 140.9, 129.4, 125.7, 122.1, 118.6, 82.7, 72.7, 72.4, 67.5, 62.6.

HR-ESI-MS calcd. for $C_{13}H_{14}N_2O_9Na^+$ [*M*+Na$^+$]: 365.05915. Found: 365.05914.

IR (neat, cm^{-1}): 3255, 1603, 1523, 1485, 1344, 1277, 1065, 973, 915, 834, 743.

$[\alpha]_D^{25}$ = +39 (*c* 0.095, MeOH).

(1S,2S,3S,4R)-5-(hydroxymethyl)cyclohex-5-ene-1,2,3,4-tetrayl tetraacetate (10).

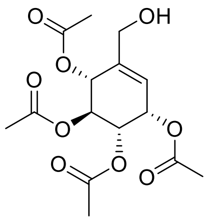

10

$C_{15}H_{20}O_9$
M = 344.32 g/mol

In a microwave vial alcohol **7** (410 mg, 0.984 mmol, 1.0 eq.) and sodium acetate (371 mg, 5.53 mmol, 4.6 eq.) were suspended in acetic anhydride (4 mL). The vial was capped and placed in an aluminium block pre-heated to 95 °C. The reaction mixture was heated to 95 °C under stirring for 1 h. The mixture was allowed to warm to RT and TLC indicated full conversion. The reaction mixture was diluted with EtOAc (4 mL) and quenched by careful addition of a sodium bicarbonate solution (sat. aq., 8 mL). The mixture was stirred vigorously until gas evolution ceased and the layers separated (ca. 30 min). The organic layer was separated, and the aqueous layer was extracted with Et_2O (2 × 15 mL). The combined organic layers were washed with brine (30 mL), dried over sodium sulfate, filtered, and concentrated *in vacuo* to yield the crude tetraacetate (crude yield: 96%).This latter was transferred to a falcon tube using THF and concentrated in vacuo. The falcon tube was capped with a septum and placed under nitrogen. Dry THF (2 mL) and dry pyridine (1.19 mL, 14.8 mmol, 15 eq.) were added and the stirred mixture was cooled to 0 °C. HF (70% in pyridine, 0.26 mL, 9.8 mmol, 10 eq.) was added dropwise at 0 °C. The reaction mixture was kept at 0 °C for 4 h and was then allowed to slowly warm to RT overnight. TLC indicated full conversion, therefore a KH_2PO_4 solution (1 M aq., 8 mL) was added dropwise, followed by Et_2O (10 mL). The organic layer was separated, and the aqueous layer was extracted with Et_2O (3 × 15 mL). The combined organic layers were washed with brine (30 mL), dried over sodium sulfate, filtered, and concentrated *in vacuo* to yield crude alcohol **10** (382 mg). The residue was purified by flash column chromatography (silica gel, Et_2O) to yield the alcohol **10** (271 mg, 0.787 mmol, 80% over two steps) as a colorless oil.

$\mathbf{R_f}$ = 0.36 (Et_2O).

^{1}H NMR (500 MHz, CDCl$_3$) δ 5.98 (dd, *J* = 5.7, 1.6 Hz, 1H), 5.68 (d, *J* = 7.3 Hz, 1H), 5.63 (app. t, *J* = 4.9 Hz, 1H), 5.59 (dd, *J* = 10.8, 7.2 Hz, 1H), 5.15 (dd, *J* = 10.8, 3.9 Hz, 1H), 4.12 – 4.02 (m, 2H), 2.11 (s, 6H), 2.05 (s, 3H), 2.02 (s, 3H).

^{13}C NMR (126 MHz, CDCl$_3$) δ 171.1, 170.4, 170.2, 169.9, 141.7, 121.7, 71.4, 69.9, 68.5, 65.6, 62.2, 21.0, 20.9, 20.9, 20.8.

HR-ESI-MS calcd for $C_{15}H_{20}O_9Na$ [M+Na]$^+$: 367.09995. Found: 367.09972.

IR (thin film, cm^{-1}): ν 3495w, 2938w, 1744s, 1433w, 1371m, 1222s, 1147w, 1114w, 1045m, 973w, 942w, 918w, 868w, 748w, 602w.

$[\alpha]_D^{25}$ = +81 (c 0.53, $CHCl_3$).

(1S,2S,3S,4R)-5-((2,6-difluorophenoxy)methyl)cyclohex-5-ene-1,2,3,4-tetrayl tetraacetate (11)

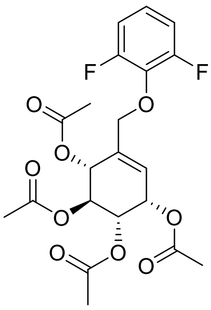

11

$C_{21}H_{22}F_2O_9$

M = 456.39 g/mol

Alcohol **10** (40.0 mg, 0.116 mmol, 1.0 eq.), 2,6-difluorophenol (30.2 mg, 0.232 mmol, 2.0 eq.), and triphenylphosphine (60.9 mg, 0.232 mmol, 2.0 eq.) were placed in a 5 mL flask equipped with a Schlenk adapter and septum. The flask was evacuated and backfilled with nitrogen (repeated three times). Dry THF (1.6 mL) was added and the stirred mixture cooled to 0 °C. DEAD (40% in toluene, 91 µL, 0.23 mmol, 2.0 eq.) was added dropwise and the reaction mixture was allowed to slowly warm up to RT overnight. TLC indicated full conversion and the reaction mixture was concentrated *in vacuo*. The resulting crude was suspended in cold Et_2O (2 mL). The suspension was filtered through a 0.22 µM filter to partially remove diethyl hydrazine-1,2-dicarboxylate. The filtrate was concentrated *in vacuo* and the resulting residue was purified using flash column chromatography (silica gel, 3:2 pentane/Et_2O) to yield the ether **11** (32.3 mg, 70.8 µmol, 61%) as a colorless oil.

$\mathbf{R_f}$ = 0.53 (pentane/Et_2O 1:2).

^{1}H NMR (500 MHz, $CDCl_3$) δ 7.02 – 6.95 (m, 1H), 6.93 – 6.85 (m, 2H), 6.10 – 6.06 (m, 1H), 5.86 – 5.82 (m, 1H), 5.64 (t, J = 4.8 Hz, 1H), 5.56 (dd, J = 10.8, 7.1 Hz, 1H), 5.19 (dd, J = 10.8, 3.9 Hz, 1H), 4.65, 4.46 (ABq, 2H, J_{AB} = 12.3 Hz), 2.10 (d, J = 1.0 Hz, 3H), 2.08 (s, 3H), 2.05 (s, 3H), 2.02 (s, 3H) ppm;

^{13}C NMR (126 MHz, $CDCl_3$) δ 170.4, 170.3, 170.1, 169.9, 156.2 (dd, J = 248.7, 5.3 Hz), 138.1, 134.8 (t, J = 14.2 Hz), 124.0, 123.8 (t, J = 9.3 Hz), 112.4 (dd, J = 17.5, 5.3 Hz), 72.8 (t, J = 3.2 Hz), 70.7, 70.1, 68.3, 65.5, 21.0, 20.9, 20.7 (2C) ppm;

^{19}F NMR (471 MHz, $CDCl_3$) δ -127.7 – -127.8 (m) ppm;

HR-ESI-MS calcd. for $C_{21}H_{22}O_9F_2Na$ $[M+Na]^+$: 479.11241; found: 479.11217;

IR (thin film): *v* 1747s, 1590w, 1498m, 1476m, 1433w, 1371m, 1293m, 1221s, 1152w, 1047m, 1008m, 944w, 919w, 783w, 727w, 601w cm^{-1};

$[\alpha]_D^{24}$ = +105 (c = 0.42, $CHCl_3$).

(1S,2S,3S,4R)-5-((2,6-difluorophenoxy)methyl)cyclohex-5-ene-1,2,3,4-tetraol (9).

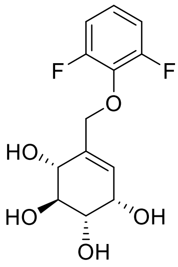

9

$C_{13}H_{14}F_2O_5$
M = 288.25 g/mol

In a 5 mL flask under nitrogen, to a solution of tetraacetate **11** (19.1 mg, 41.8 µmol, 1.0 eq.) in dry methanol (1 mL) was added a catalytic amount of potassium carbonate (ca. 25 mol%). The reaction mixture was stirred at RT for 4 h and TLC indicated full conversion.
The reaction mixture was concentrated *in vacuo* and was purified directly by flash column chromatography (silica gel, 6-10% MeOH in DCM) to yield streptol derivative **9** (8.0 mg, 27.8 µmol, 66%) as a colorless solid.

$\mathbf{R_f}$ = 0.19 (DCM/MeOH 9:1);

^{1}H NMR (500 MHz, MeOD-*d4*) δ 7.08 – 7.01 (m, 1H), 7.01 – 6.93 (m, 2H), 5.90 (dd, *J* = 5.2, 1.5 Hz, 1H), 4.91, 4.57 (ABq, 2H, J_{AB} = 12.3 Hz), 4.16 (t, *J* = 4.7 Hz, 1H), 4.13 – 4.10 (m, 1H), 3.74 (dd, *J* = 10.1, 7.3 Hz, 1H), 3.44 (dd, *J* = 10.1, 4.2 Hz, 1H).ppm;

^{13}C NMR (126 MHz, MeOD-*d4*) δ 157.7 (dd, *J* = 247.0, 5.5 Hz), 140.1, 136.4 (t, *J* = 14.3 Hz), 126.9, 124.6 (t, *J* = 9.4 Hz), 113.2 (dd, *J* = 17.5, 5.5 Hz), 74.9 (t, *J* = 3.3 Hz), 74.1, 73.2, 72.5, 67.6 ppm;

^{19}F NMR (471 MHz, MeOD-*d4*) δ -129.7 – -129.8 (m) ppm;

IR (thin film): *v* 3328m, 2911w, 1592w, 1498s, 1475s, 1293m, 1239m, 1103m, 1060m, 1006s, 910w, 882w, 838w, 778m, 720w, 673w, 622w, 577w, 503w cm^{-1};

HRMS (ESI) for $C_{13}H_{14}O_5F_2Na$ $[M+Na]^+$: calculated: 311.07015; found: 311.07005;

$[\alpha]_D^{25}$ = +114 (c = 0.16, MeOH).

(1S,2S,3S,4R)-5-((3,5-difluorophenoxy)methyl)cyclohex-5-ene-1,2,3,4-tetrayl tetraacetate (11').

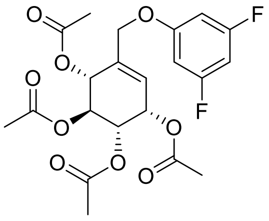

11'

$C_{21}H_{22}F_2O_9$
M = 456.39 g/mol

Alcohol **10** (31.0 mg, 90.1 µmol, 1.0 eq.), 3,5-difluorophenol (23.4 mg, 0.182 mmol, 2.0 eq.), and triphenylphosphine (47.2 mg, 0.182 mmol, 2.0 eq.) were placed in a 5 mL flask equipped with a Schlenk adapter and septum. The flask was evacuated and backfilled with nitrogen (repeated three times). Dry THF (0.5 mL) was added and the stirred mixture cooled to 0 °C. DEAD (40% in toluene, 71 µL, 0.18 mmol, 2.0 eq.) was added dropwise and the reaction mixture was stirred at 0 °C for 2 h. TLC indicated full conversion and the reaction mixture was concentrated *in vacuo*. The resulting crude was suspended in cold Et_2O (2 mL). The suspension was filtered through a 0.22 µM filter to remove diethyl hydrazine-1,2-dicarboxylate. The filtrate was concentrated *in vacuo* and the resulting residue was purified using flash column chromatography (silica gel, 3:1 to 3:2 pentane/Et_2O) to yield ether **11'** (38.0 mg, 83.3 µmol, 93%) as a colorless oil.

R_f = 0.38 (pentane/Et_2O 3:2);

^{1}H NMR (500 MHz, $CDCl_3$) δ 6.48 – 6.41 (m, 1H), 6.42 – 6.36 (m, 2H), 6.11 – 6.01 (m, 1H), 5.79 – 5.74 (m, 1H), 5.67 – 5.63 (m, 1H), 5.57 (dd, *J* = 10.8, 7.2 Hz, 1H), 5.18 (dd, *J* = 10.8, 3.9 Hz, 1H), 4.48, 4.41 (ABq, 2H, J_{AB} = 12.7 Hz), 2.11 (s, 3H), 2.05 (s, 3H), 2.03 (s, 3H), 2.03 (s, 3H) ppm;

^{13}C NMR (126 MHz, $CDCl_3$) δ 170.3, 170.3, 170.1, 169.9, 163.8 (dd, *J* = 247.1, 15.6 Hz), 159.9 (t, *J* = 13.5 Hz), 137.5, 123.5, 98.9 – 98.4 (m), 97.4 (t, *J* = 25.9 Hz), 70.8, 70.0, 68.2, 67.5, 65.4, 21.0, 20.9, 20.7 (2C) ppm;

^{19}F NMR (471 MHz, $CDCl_3$) δ -108.6 – -108.7 (m) ppm;

IR (thin film): *ν* 2926w, 2854w, 1743s, 1622m, 1599m, 1471m, 1370m, 1251m, 1215s, 1151m, 1118m, 1045m, 1023m, 1004m, 996m, 943m, 919m, 841m, 767w, 671w, 601w, 541w, 524w, 508w, 485w cm^{-1};

HRMS (ESI) for $C_{21}H_{22}O_9F_2Na$ $[M+Na]^+$: calculated: 479.11241; found: 479.11229;

$[\alpha]_D^{24}$ = +87 (c = 0.76, $CHCl_3$).

(1S,2S,3S,4R)-5-((3,5-difluorophenoxy)methyl)cyclohex-5-ene-1,2,3,4-tetraol (9').

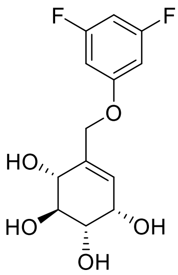

9'

$C_{13}H_{14}F_2O_5$
M = 288.25 g/mol

In a 5 mL flask under nitrogen, to a solution of tetraacetate **11** (28.0 mg, 61.4 μmol, 1.0 eq.) in dry methanol (1 mL) was added a catalytic amount of potassium carbonate (ca. 25 mol%). The reaction mixture was stirred at RT for 4 h and TLC indicated full conversion. The reaction mixture was concentrated *in vacuo* onto celite and was purified directly by flash column chromatography (silica gel, 10% MeOH in DCM) to yield streptol derivative **9'** (10.9 mg, 28.4 μmol, 47% (ca. 75% purity) as a colorless solid.

$\mathbf{R_f}$ = 0.22 (DCM/MeOH 9:1);
^{1}H NMR (500 MHz, MeOD-d_4) δ 6.59 (td, J = 9.1, 8.3, 2.3 Hz, 2H), 6.50 (tt, J = 9.2, 2.3 Hz, 1H), 5.92 (dd, J = 5.2, 1.6 Hz, 1H), 4.72, 4.58 (ABq, 2H, J_{AB} = 12.8 Hz), 4.21 (t, J = 4.7 Hz, 1H), 4.04 – 3.99 (m, 1H), 3.77 (dd, J = 10.0, 7.2 Hz, 1H), 3.48 (dd, J = 10.0, 4.2 Hz, 1H) ppm;
^{13}C NMR (126 MHz, MeOD-d_4) δ 165.2 (dd, J = 244.9, 15.9 Hz), 162.4 (t, J = 13.7 Hz), 139.4, 126.0, 99.8 – 99.2 (m), 97.0 (t, J = 26.5 Hz), 74.1, 73.4, 72.5, 69.4, 67.5 ppm;
^{19}F NMR (471 MHz, MeOD-d_4) δ -111.4 – -111.6 (m) ppm;
IR (thin film): ν 3332m, 2920w, 1625s, 1599s, 1468m, 1411w, 1349w, 1149s, 1117s, 1057m, 1039m, 995m, 911w, 880w, 835m, 670w, 531w, 508w cm^{-1};
HRMS (ESI) for $C_{13}H_{14}O_5F_2Na$ $[M+Na]^+$: calculated: 311.07015; found: 311.07002;
$[\alpha]_D^{24}$ = *not measured due to low solubility.*

Note: The compound is nearly insoluble in DCM, MeOH, H_2O, and DMSO.

General experimental methods: biology

Yeast α-glucosidase was purchased from Sigma Aldrich (GH13, G0660, activity 213 U/mgP or 42 U/mgS and GH13, G5003, activity 41 U/mgP or 24.2 U/mgS). Stock solutions for inactivation and reactivation assays were prepared using sodium phosphate buffer (50 mM, pH 6.84) containing BSA (1 mg/ml). In both types of tests, the residual enzymatic activity was measured using a 50 μM 4-nitrophenyl α-D-glucopyranoside solution in sodium phosphate buffer (50 mM, pH 6.84) containing BSA (1 mg/ml). Round-bottomed 96 well plates (Thermo Scientific) were used for all the measurements. The rate of production of 4-nitrophenol was monitored at 405 nm using a plate reader Synergy H1 (BioTek). The measured absorption data was analyzed using Prism 6.0.

Biological assessment of the inhibitors[2]

Standard procedure for inactivation kinetics with G0660 and G5003

Preparation of the "incubation plate":

A 0.2 mg/ml stock solution of the enzyme in phosphate buffer (50 mM, 1 mg/mL BSA; pH 6.84) was prepared freshly, stored at 4 °C and used within one day. The "incubation well-plate" was prepared by diluting 20 μL of the stock solution of the enzyme into 140 μL of pre-dispensed phosphate buffer. The phosphate buffer was used at RT, stored at 4 °C and used within two months. Classical dilution method was applied to prepare 5-fold solutions of the inhibitor in deionized water. The inhibitor solutions were prepared freshly and used within one day. 40 μL of the respective inhibitor solution (spanning a concentration range from 5 – 1000 μM (**2**)/ 25 – 1000 μM (**3**)/ 125 – 3000 μM (**9**) in the incubation plate V_{final}: 200 μL) was added to the solution of enzyme in buffer. For the control in the absence of inhibitor, the procedure was identical as described above but deionized water was used instead of inhibitor solution.
The enzyme was incubated with the inhibitor at RT for the indicated period of time, before an aliquot of 10 μL of each well was diluted into 90 μL of 4-nitrophenyl α-D-glucopyranoside solution (50 μM in phosphate buffer) (see kinetic plates and absorbance reading).

Preparation of the "kinetic plates" and absorbance reading:

A 50 μM solution of 4-nitrophenyl α-D-glucopyranoside in phosphate buffer was prepared freshly, stored at RT and used within one day. At the indicated time points $t_{incubation}$, the residual enzymatic activity was measured: 10 μL of the respective wells of the "incubation plate" was added to 90 μL of pre-dispensed solution of 4-nitrophenyl α-D-glucopyranoside in phosphate buffer (50 μM) in the "kinetic plates" and the absorbance at 405 nm was monitored over 30 to

40 min (t_{read}) in 16 or 18 s intervals. This step was repeated at the indicated time points $t_{incubation}$ (7 min 19 s, 53 min 1 s, 1 h 58 min 8 s, 2 h 56 min 27 s in the case of **2** with enzyme lot G5003) using new kinetic plates for each time points $t_{incubation}$.

The experiment was performed in technical triplicate.

The curves corresponding to the absorbance as a function of t_{read} for the wells containing 5 µM, 50 µM and 250 µM of **2** (concentration in the incubation plate) are shown in Figure S1 as an example. The different time points ($t_{incubation}$ on the legend on the right) refer to the data obtained for the different kinetic plates. The control refers to the data obtained in the absence of inhibitor and is shown as mean ± SD combining the data collected throughout all kinetic plates.

A

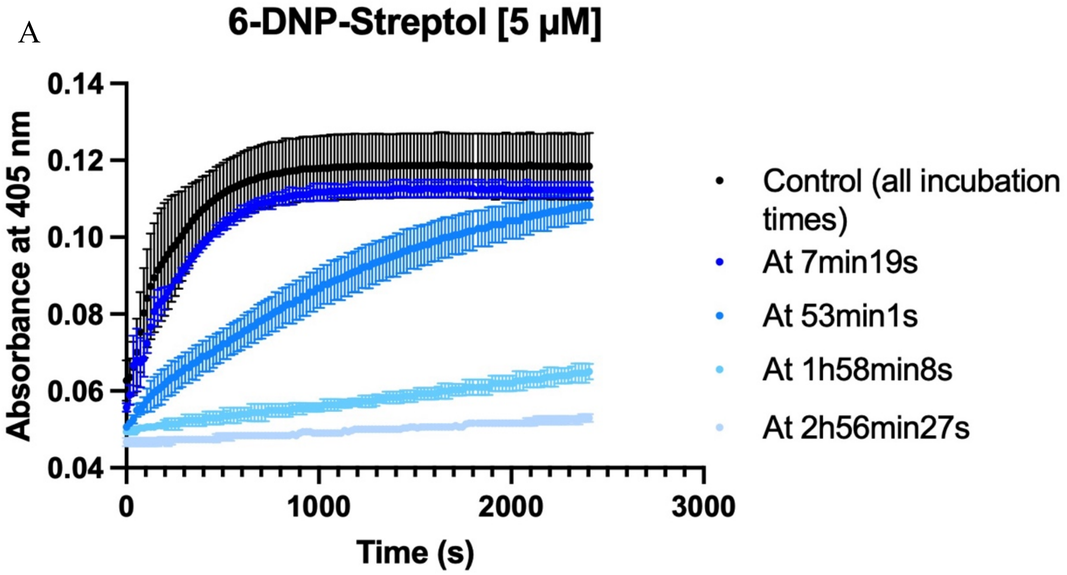

B

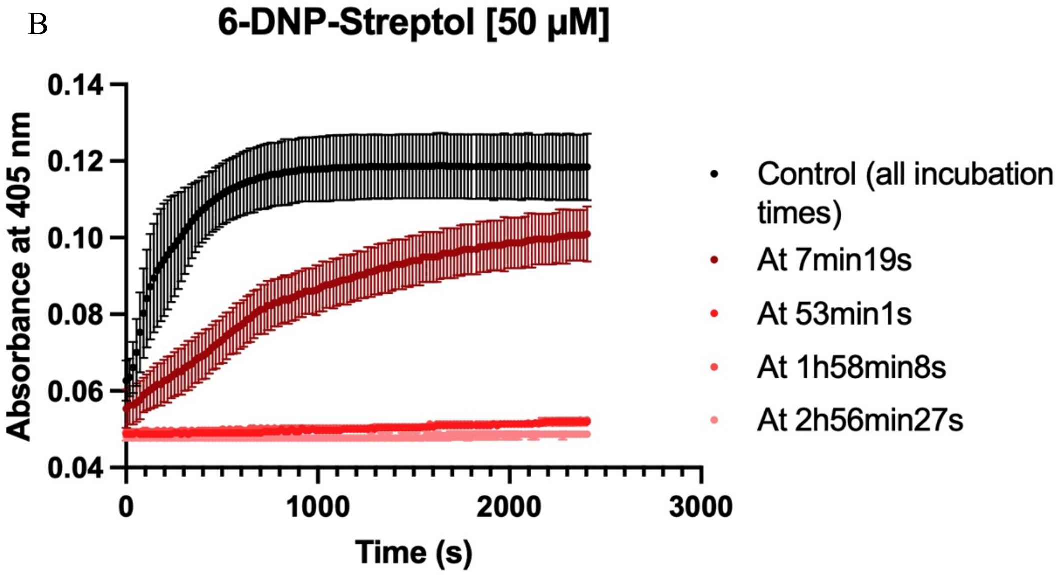

C

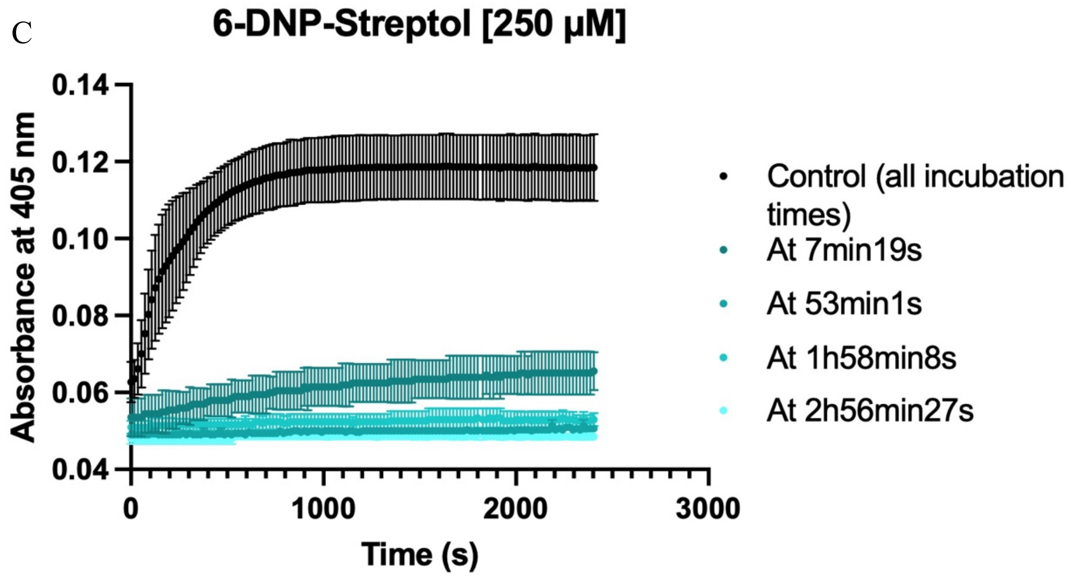

Figure S1: Enzyme was incubated with **2** (A: 5 µM; B: 50 µM; C: 250 µM) for the indicated incubation time before dilution (legend on the right) into PNP-Glc solution. The absorbance at 405 nm was monitored for 40 min in 18 s intervals. Each data point refers to mean±SD (n=2-3)

The initial velocities of each enzymatic reaction were normalized and plotted against the incubation time for each inhibitor concentration (Figure 2, S2 and S3). These curves were fitted to a standard one phase exponential decay equation to access k_{obs} by using Prism 6.0. The k_{obs} values were plotted against the corresponding inhibitor concentrations and fitted to a standard Michaelis-Menten equation to access k_{inact} and K_i and the respective standard deviation ±SD (Figures 3 and S4).

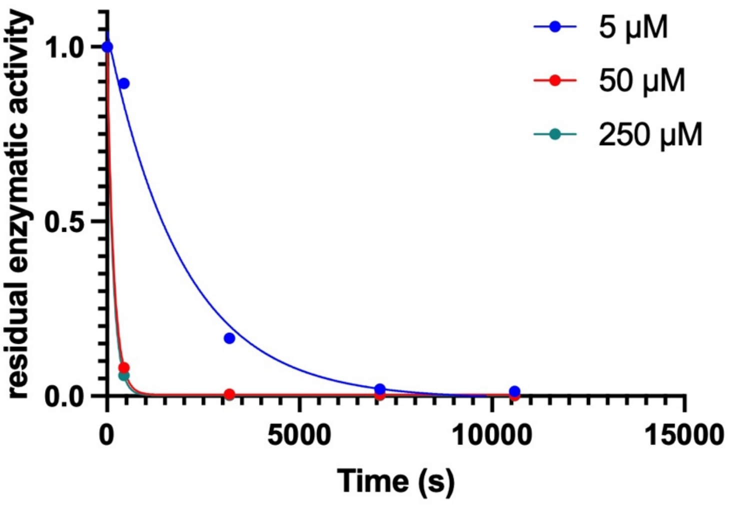

Figure S2 Residual enzymatic activity evolution over time of GH13 yeast α-glucosidase (G5003) incubated with **2** at concentration indicated on the legend on the right

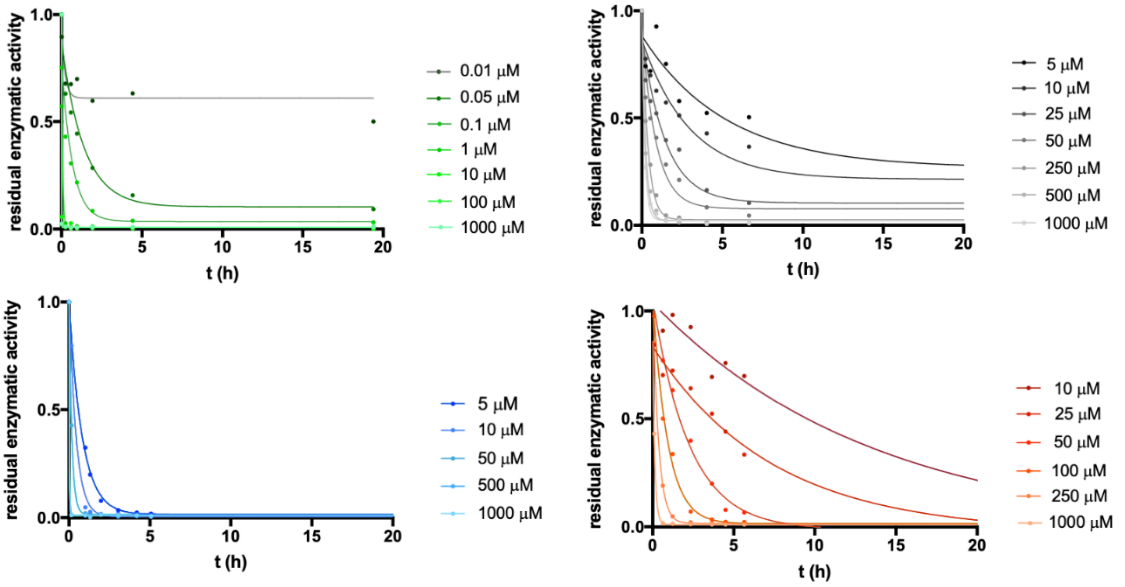

Figure S3. Residual enzymatic activity evolution over time of GH13 yeast α-glucosidase (G0660) incubated with **1** (top-left, green), **1’** (top-right, grey), **2** (bottom-left, blue), and **3** (bottom-right, red) at different concentrations.

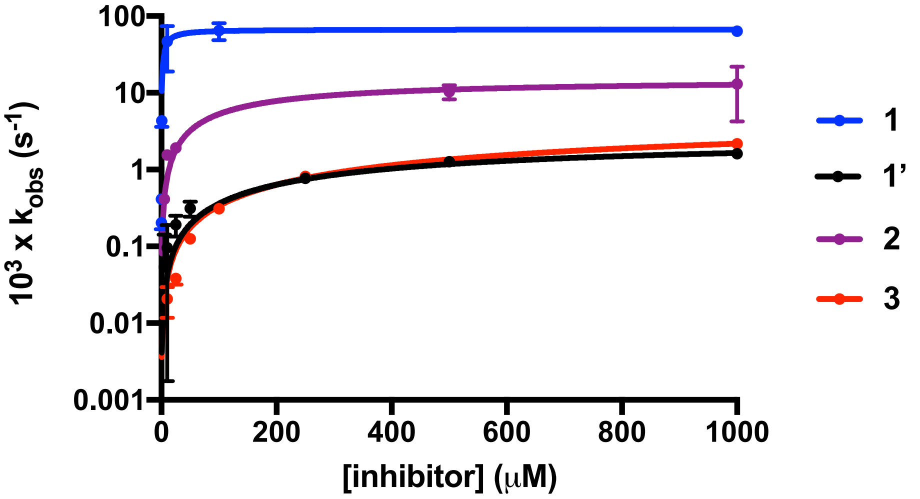

Figure S4. Pseudo-first order of reaction with GH13 yeast α-glucosidase (G0660) in function of concentration of inhibitor **1** (blue), **1’** (black), **2** (purple), and **3** (red). The lines correspond to best non-linear fit to a standard Michaelis-Menten equation. (The non-visible error bars are encompassed within the data point symbol).

Standard procedure for reactivation kinetics

A 1.0 mg/ml stock solution of the enzyme was prepared. 10 μL of this solution were placed in several Eppendorf tubes, followed by 10 μL of 10 mM solutions of the studied inhibitors in deionized water or 10 μL of deionised water for the control sample. The mixtures were incubated for 60 min at 25 °C. They were then transferred into molecular weight cutoff filters (5k, Vivaspin 2). The Eppendorf tubes were washed with 2 x 45 μL of a 1 mg/mL solution of BSA in a sodium phosphate buffer (pH 6.84). The filters were centrifuged at 4 °C and 4350 rpm for 15 min. 500 μL of the 1 mg/mL BSA solution were added to the filter and the centrifugation was repeated. The filters were washed twice in a similar manner using 500 and 250 μL of the same BSA solution. For each sample, the liquid remaining on the filter was

recovered and placed in an Eppendorf tube. The filters were washed with 3 x 70 µL of the BSA solution and the washes were combined. The solutions were incubated at 25 °C. The residual enzymatic activities were measured as described above in duplicates.

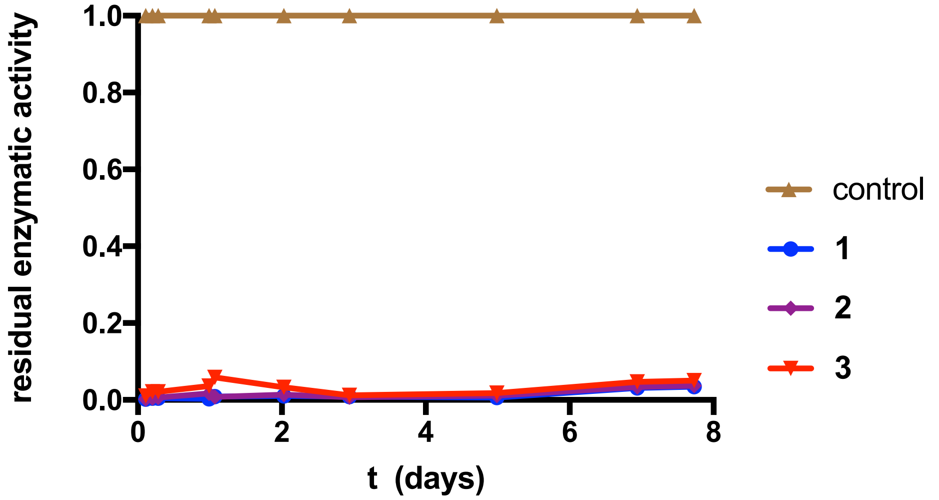

Figure S5. Residual enzymatic evolution of GH13 yeast α-glucosidase (G5003) incubated with inhibitors **1** (blue), **2** (purple) and **3** (red).

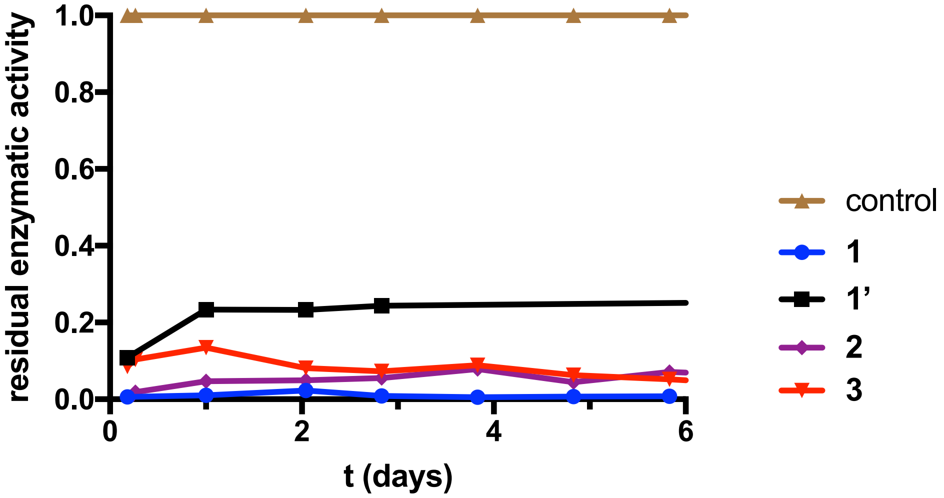

Figure S6. Residual enzymatic evolution of GH13 yeast α-glucosidase (G0660) incubated with inhibitors **1** (blue), **1'** (black), **2** (purple) and **3** (red)

Study of enzyme-inhibitor adducts by mass-spectrometry

Standard procedure for the adduct preparation

Each GH13 yeast α-glucosidase G0660 and G5003 (1 mg/ml, 10 µL) were incubated with the inhibitor (10 mM, 10 µL) in deionised water for 1 hour. Prior ESI-MS analysis, samples were desalted using 7K SEC cartridges (Thermo, USA) and analyzed in MeOH:2-PrOH:0.2% FA (30:20:50). The solution was infused through a fused silica capillary (ID75um) at a flow rate of 1 µL/min and sprayed through a PicoTips (ID30um). The latter were obtained from New Objective (Woburn, MA). Nano ESI-MS analyses of the samples were performed on a Synapt

G2_Si mass spectrometer and the data were recorded with the MassLynx 4.2 Software (both Waters, UK). Mass spectra were acquired in the positive-ion mode by scanning an m/z range from 100 to 5000 Da with a scan duration of 1 s and an interscan delay of 0.1s. The spray voltage was set to 3 kV, the cone voltage to 100 V, and source temperature 80 °C. The recorded m/z data were then deconvoluted into mass spectra by applying the maximum entropy algorithm MaxEnt1 (MaxLynx) with a resolution of the output mass 0.5 Da/channel and Uniform Gaussian Damage Model at the half height of 0.5 Da.

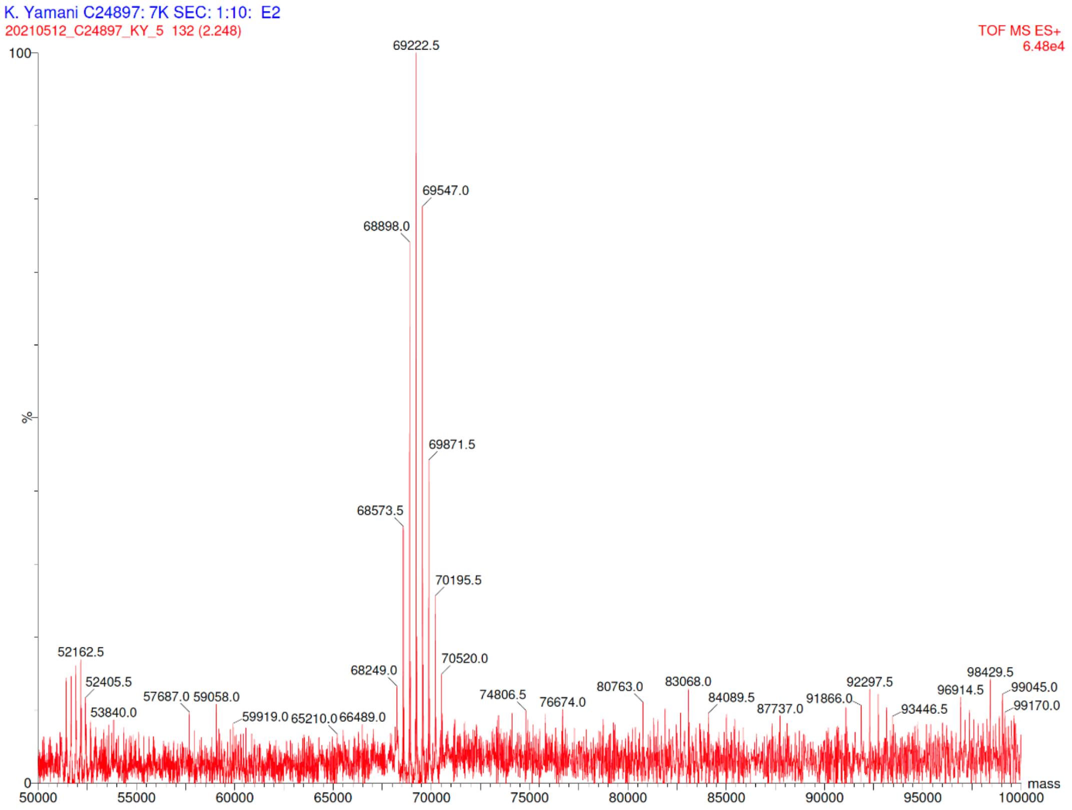

Figure S7. Reconstructed mass spectrum of the free enzyme G0660

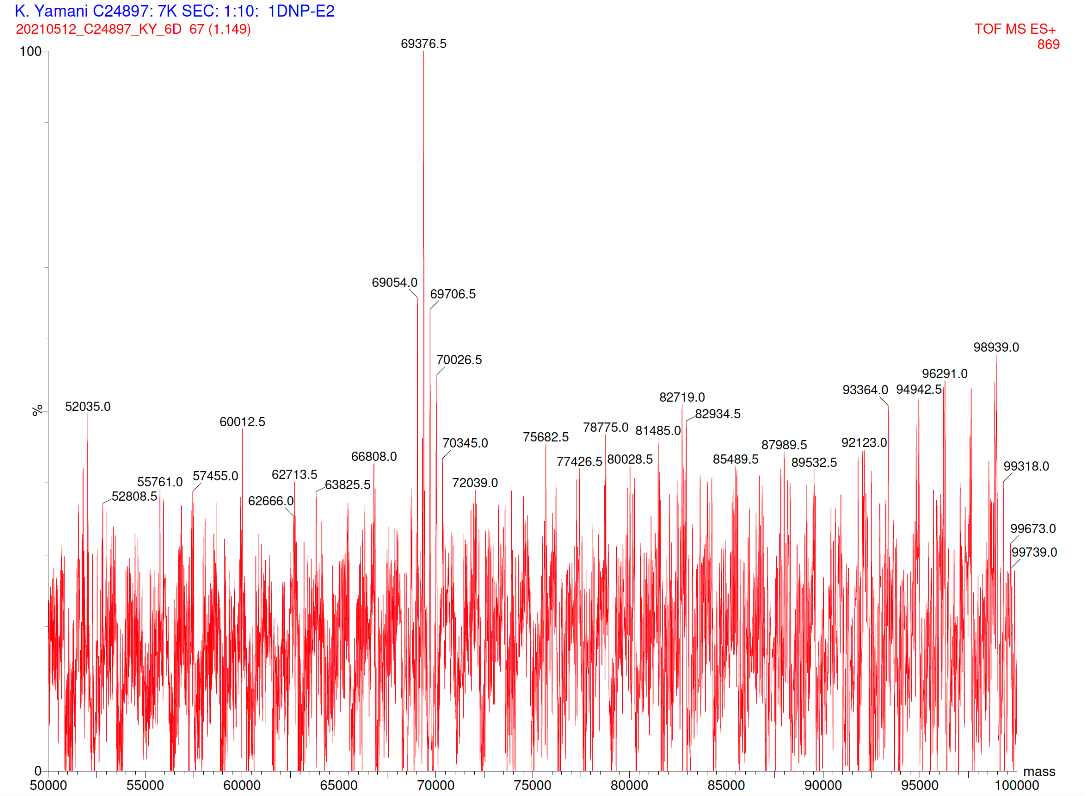

Figure S8. Reconstructed mass spectrum of adduct of enzyme G0660 with **1**

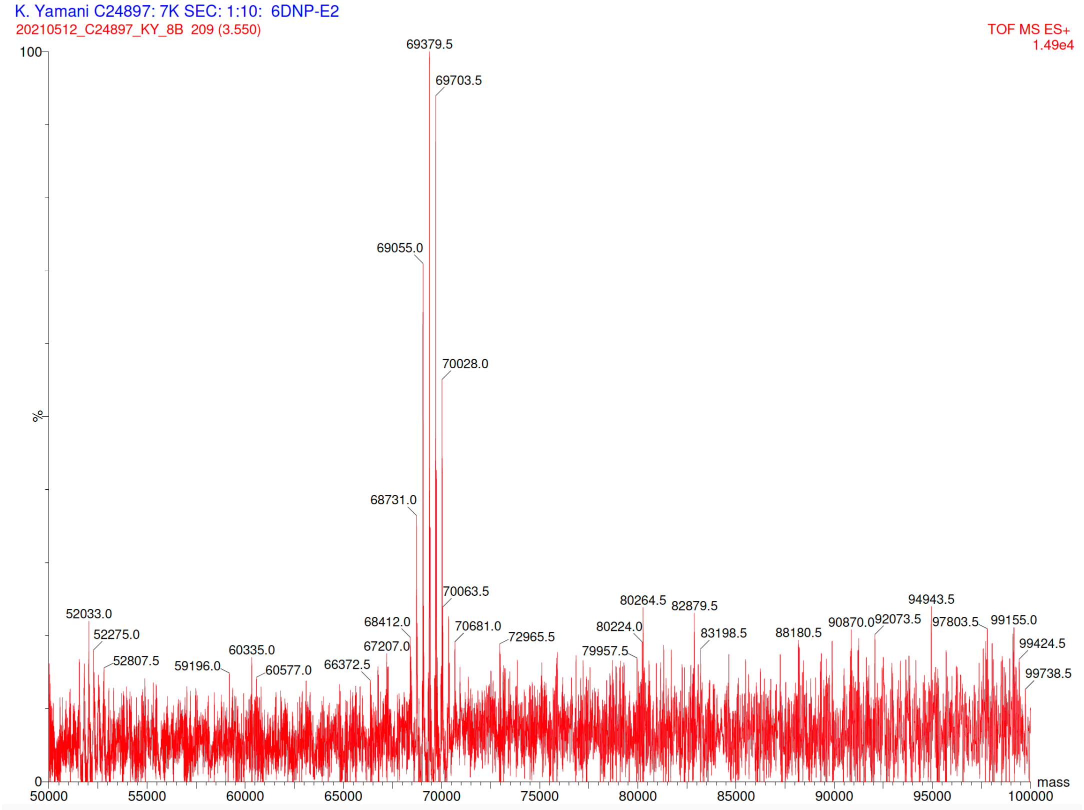

Figure S9. Reconstructed mass spectrum of adduct of enzyme G0660 with **2**

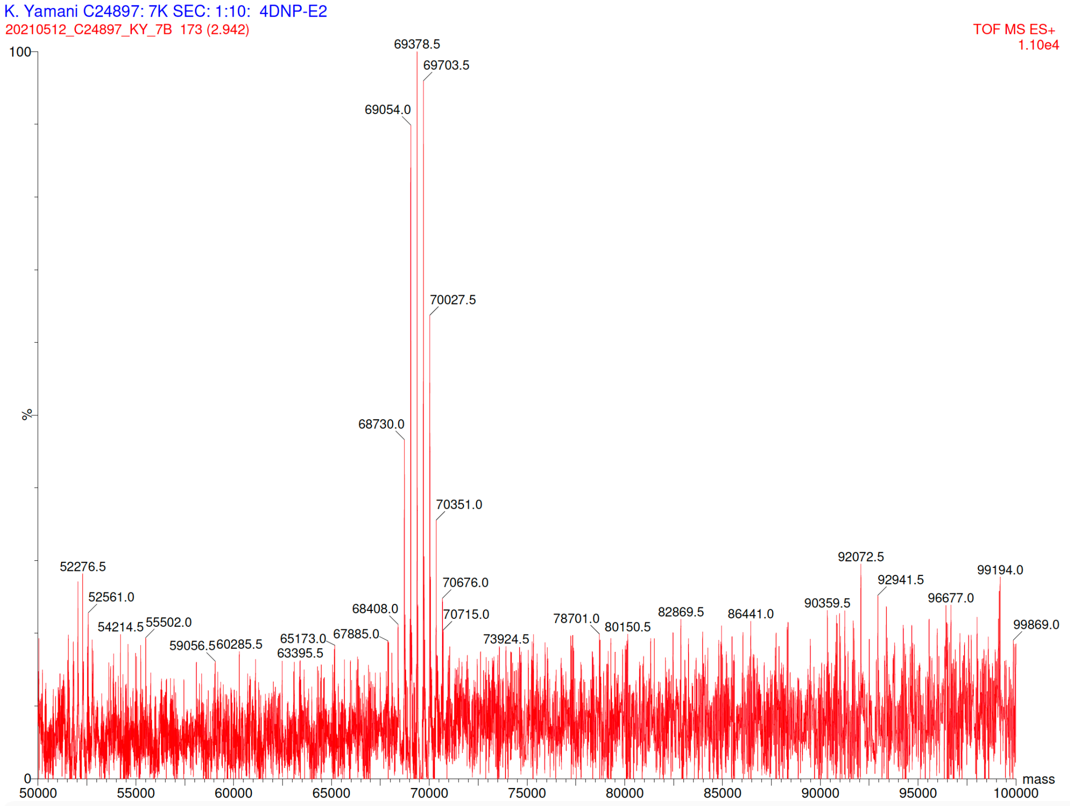

Figure S10. Reconstructed mass spectrum of adduct of the enzyme G0660 with **3**

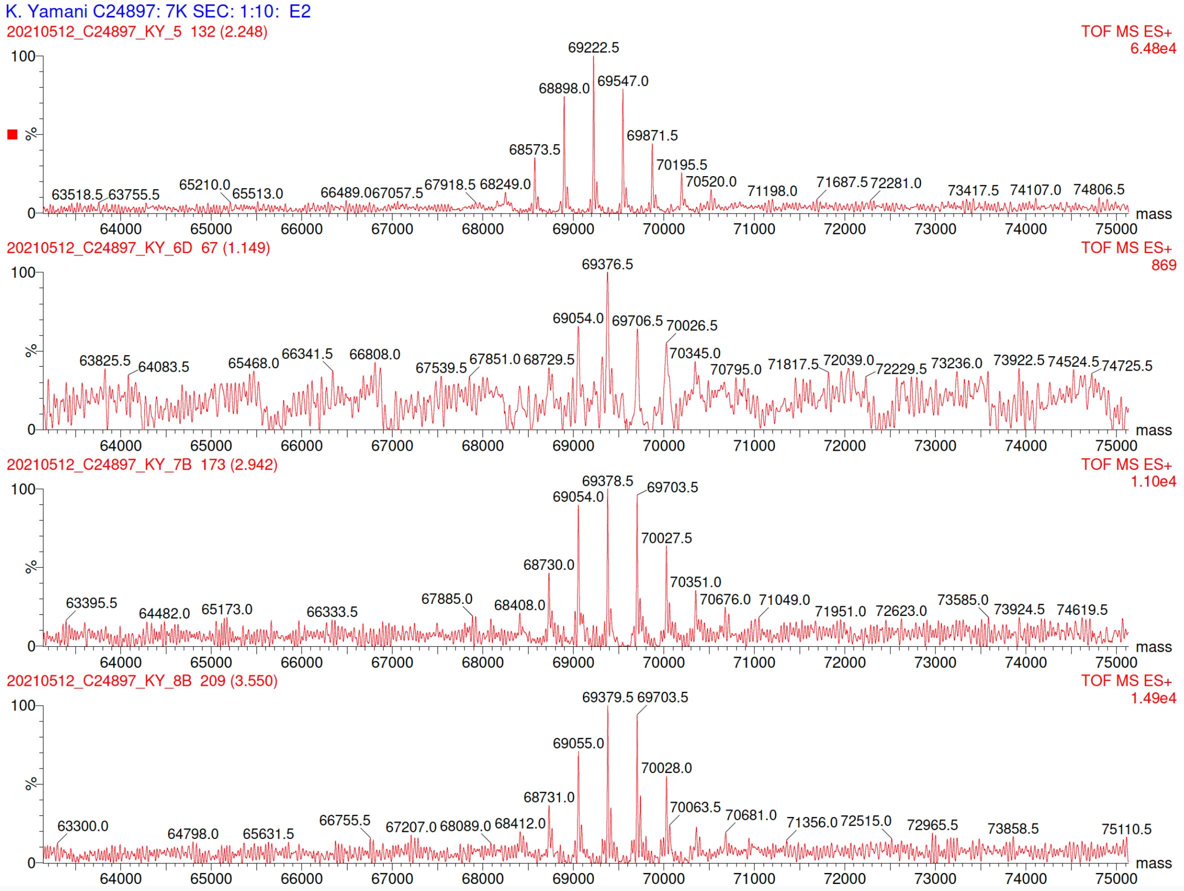

Figure S11. Zoom on the 63200-75100 Da region of the G0660 mass spectra

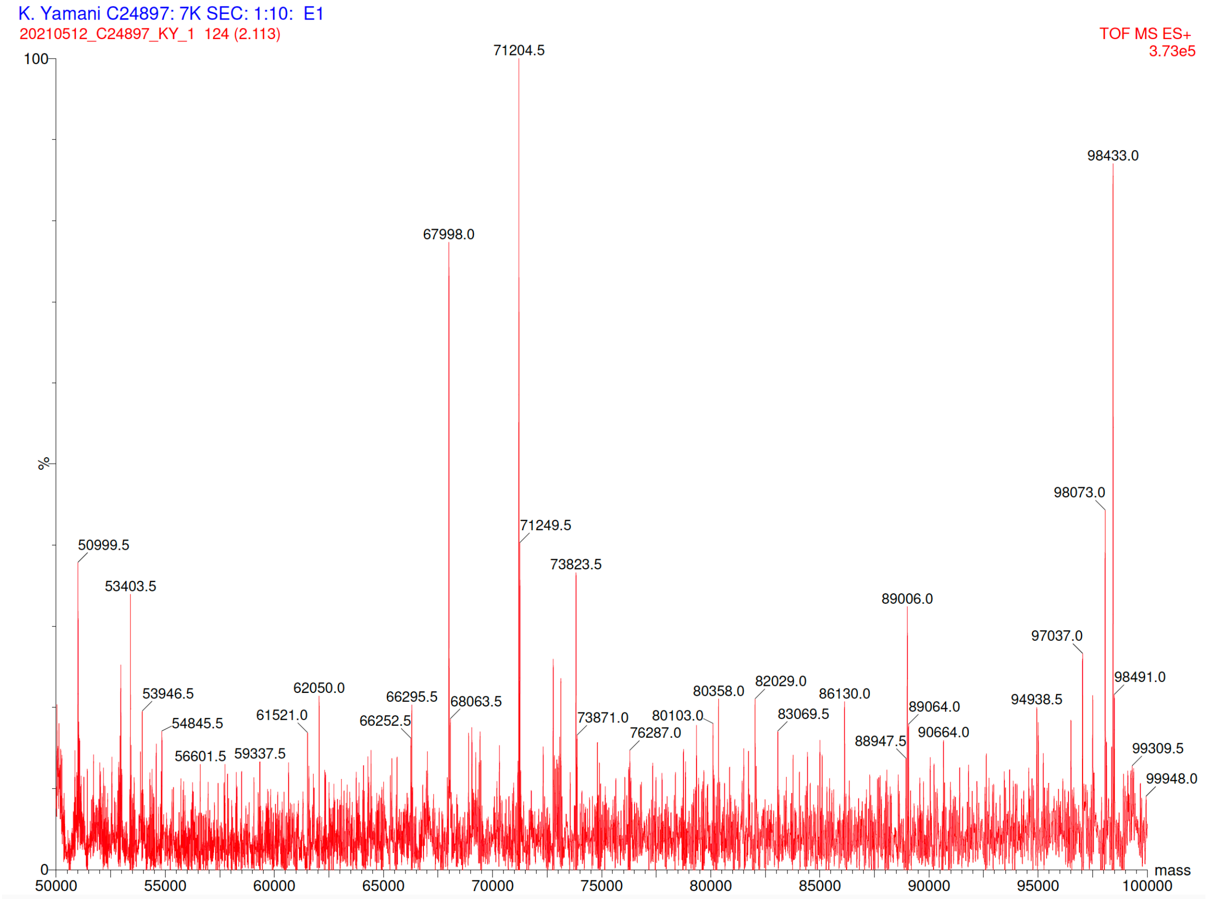

Figure S12. Reconstructed mass spectrum of the free enzyme G5003

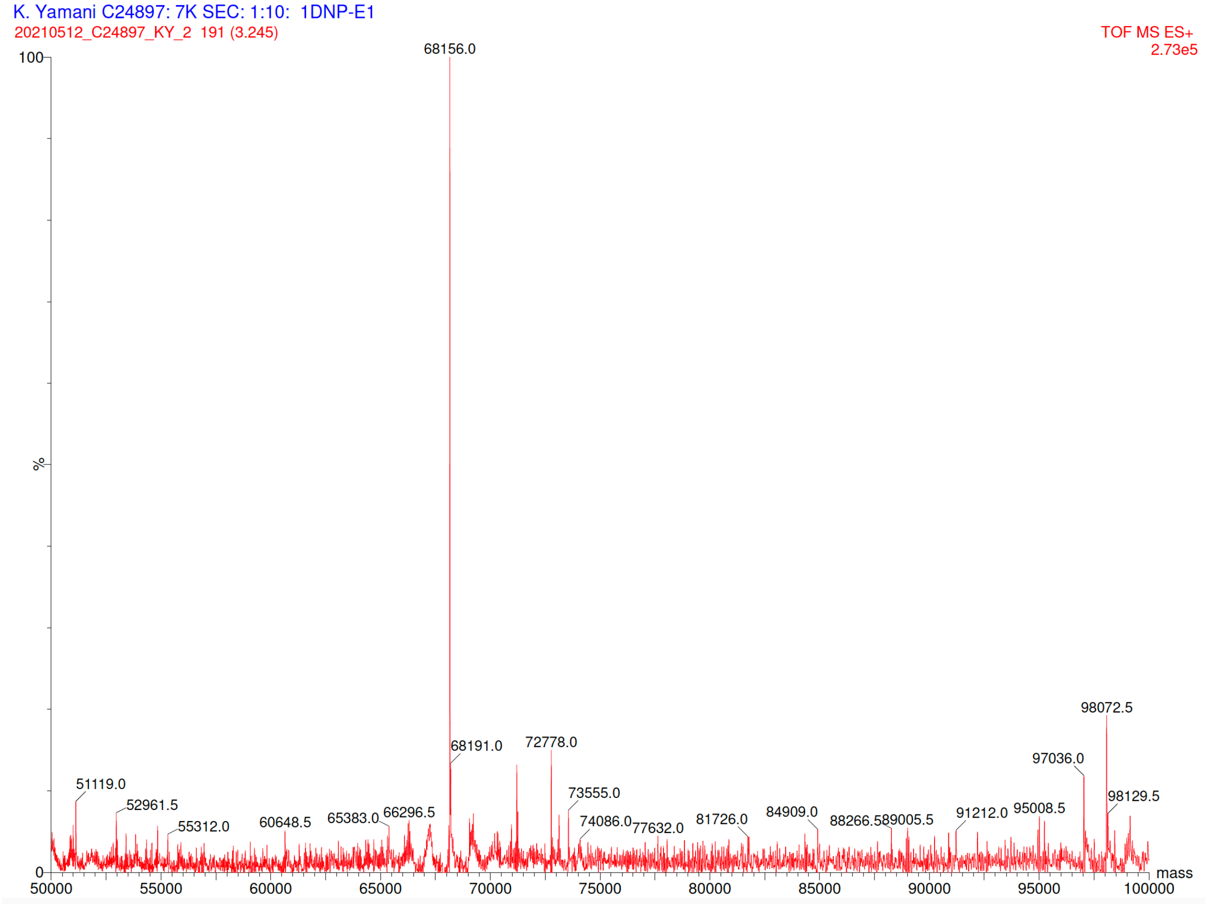

Figure S13. Reconstructed mass spectrum of adduct of enzyme G5003 with **1**

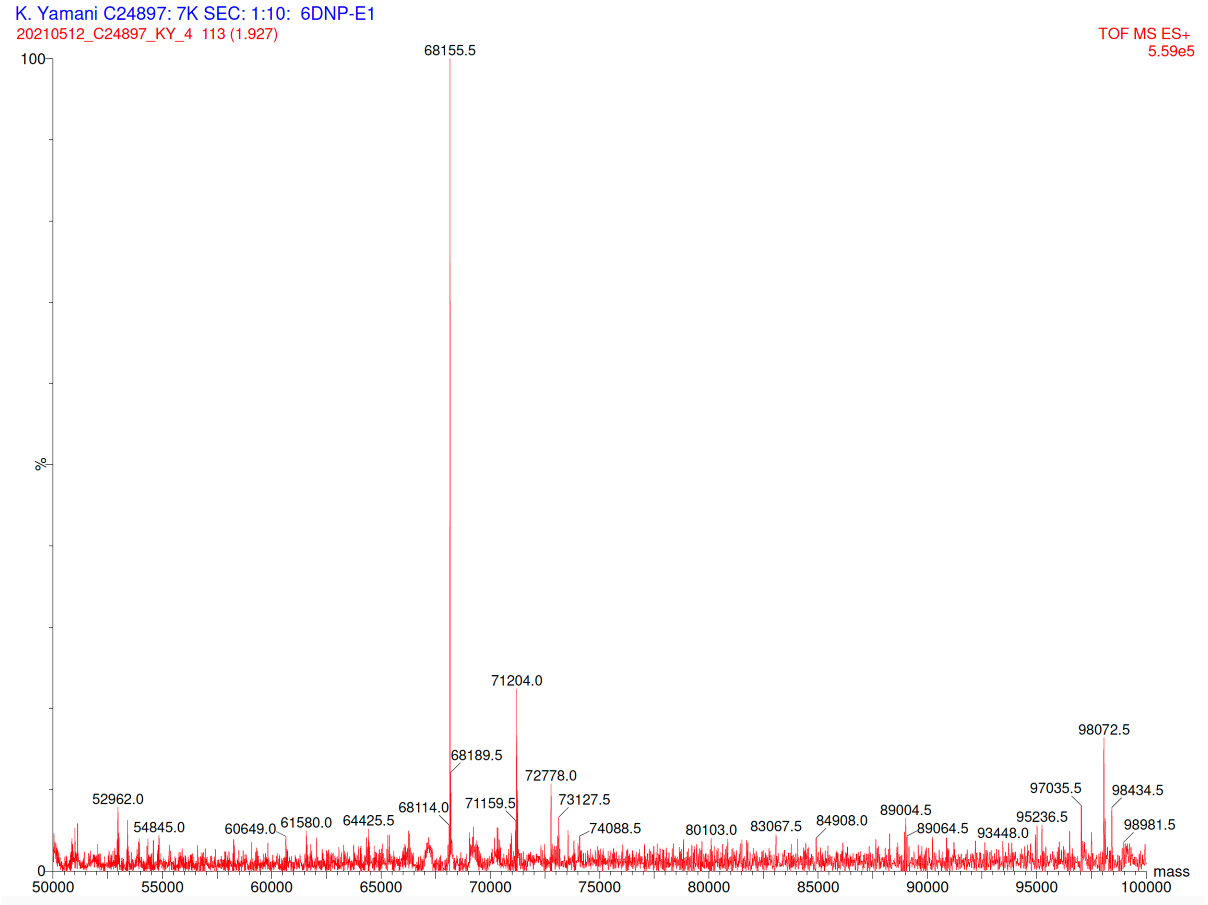

Figure S14. Reconstructed mass spectrum of adduct of enzyme G5003 with **2**

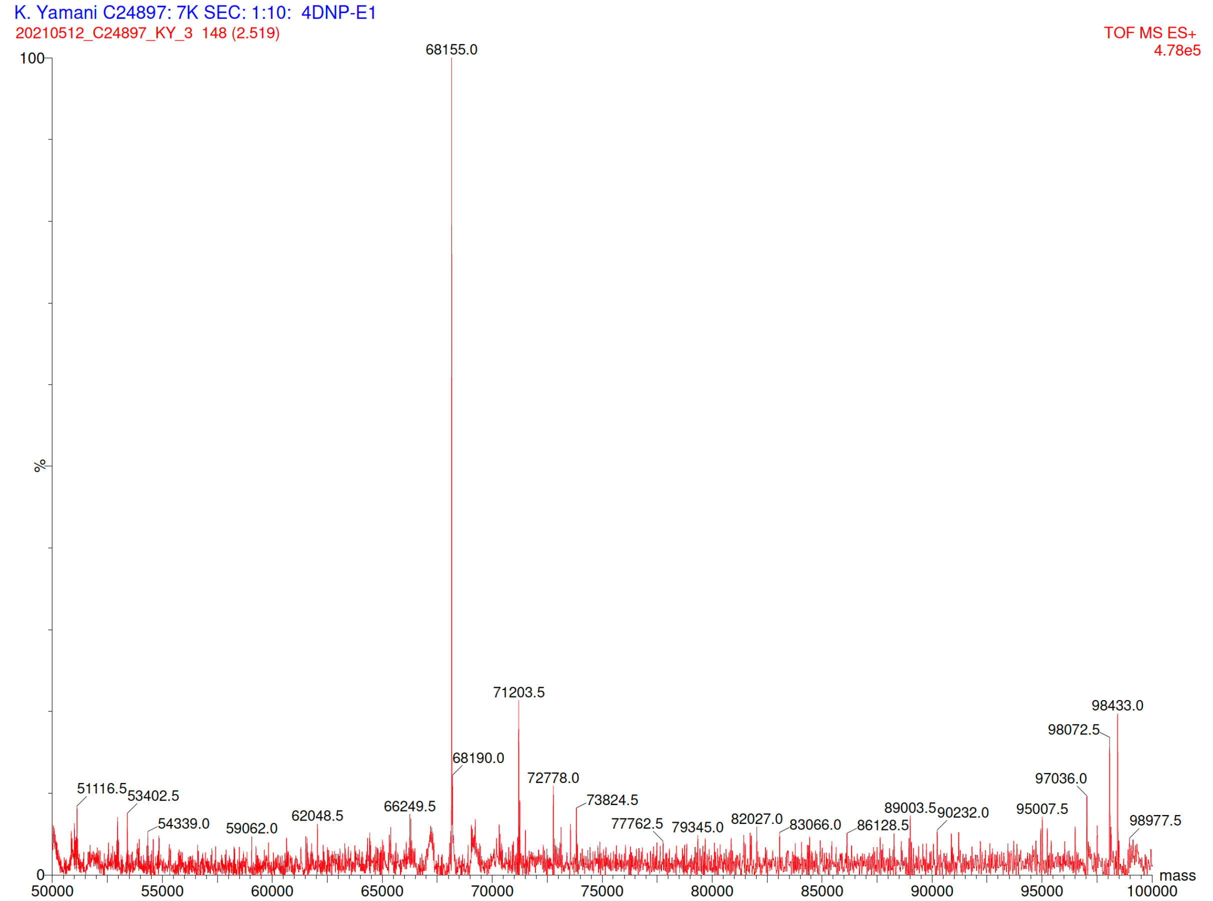

Figure S15. Reconstructed mass spectrum of adduct of enzyme (G5003) with **3**

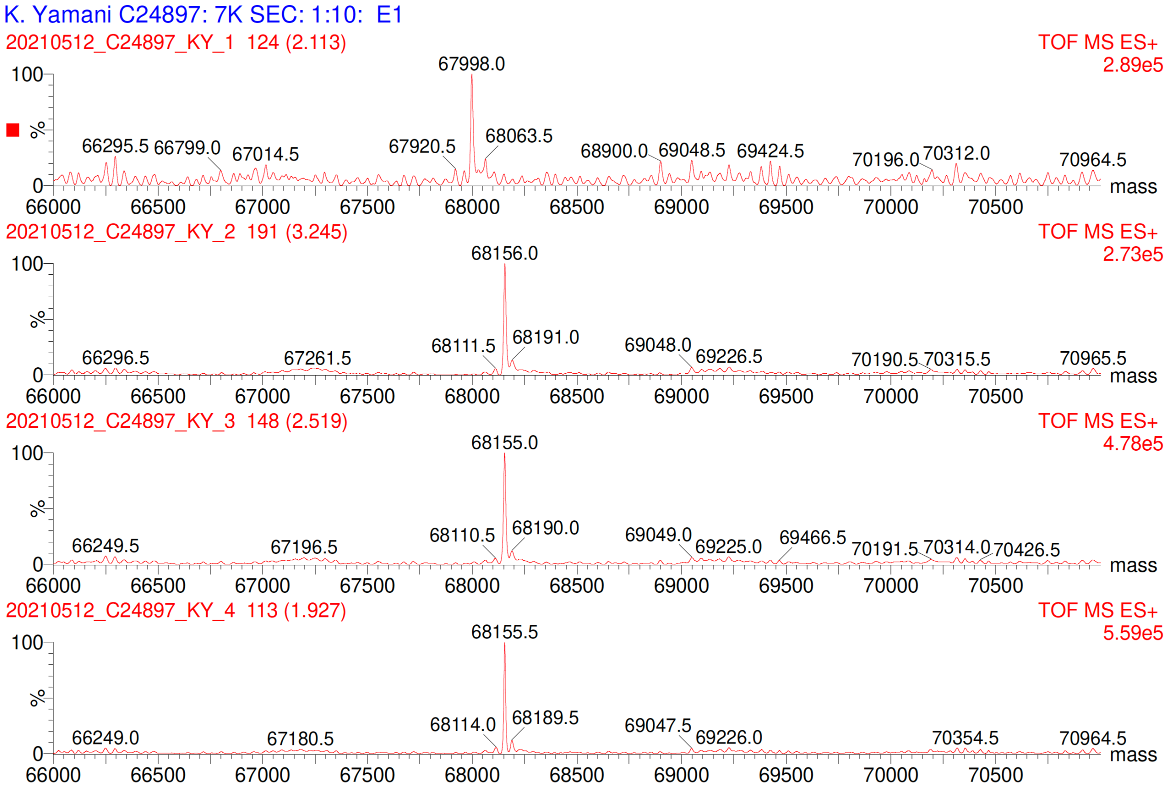

Figure S16. Zoom on the 66000-71000 Da region of the G5003 mass spectra

Site Mapping of Inhibitor Alkylation on the α-glucosidase from *Saccharomyces cerevisiae.*

Sample preparation

Each GH13 yeast α-glucosidase G0660 and G5003 (1 mg/ml, 10 µL) were incubated with inhibitor **1**, **2**, and **3** (10 mM, 10 µL) in deionised water for 1 hour.

In-solution protein digestion

To 5 µl of each sample were added 40 µl of 10 mM Tris/2 mM $CaCl_2$ (pH 8.2 buffer), 0.9 µl TCEP (100 mM), 1.4µl IAA (500mM), and 1.2 µl trypsin (400 ng/µl in 10 mM HCl). If needed pH was adjusted to pH 8. Then, the digestion was assisted by microwave (60° C, 30 min) and the samples were dried.

Data acquisition for LC-MS/MS analysis

The digested samples were dried and dissolved in 20 µl of distilled water with 0.1% formic acid. They were then transferred to the autosampler vials for Liquid chromatography-mass spectrometry analysis (LCMS/MS). 4 µl were injected on a nanoAcquity UPLC coupled to a Q-Exactive mass spectrometer (Thermo).

Data analysis on Mascot

The acquired Mass Spectroscopy data were converted to a Mascot Generic File format (.mgf files) and were processed for identification using the Mascot search engine (Matrixscience). The spectra were searched against the Yeast database

The following modifications were set:

- Variable modifications: Oxidation (M)
- Fixed modifications: None

The data were further searched with Error-tolerance option.

Data analysis on Byonic

The acquired Mass Spectrometry data were loaded into Byonic 3.4 (PMI) and searched against yeast database with consideration of oxidation at methionine, carbamidomethylation at cysteine, acetylation at the N-terminal of proteins, streptol (+158.06) modification at aspartic acid, and hexose at serine and threonine.

These data clearly show an alkylation on D214 and D5 of both batches of α-glucosidase.

Table S1: Results from trypsin-mediated digestion and analysis by Mascot and Byonic software

Incubated inhibitor	Enzyme lot	Sequence coverage of MAL32 from Mascot	Labeled amino acid	Byonic Score
**1**	G5003	77%	Asp214	248.8
**2**	G5003	84%	Asp214	333.5
**3**	G5003	83%	Asp5	69.8
**1**	G0660	65%	Asp214	255.7
**2**	G0660	53%	Asp5<br>Asp214	511.5<br>66.1
**3**	G0660	65%	Asp5<br>Asp214	465.8<br>228.2

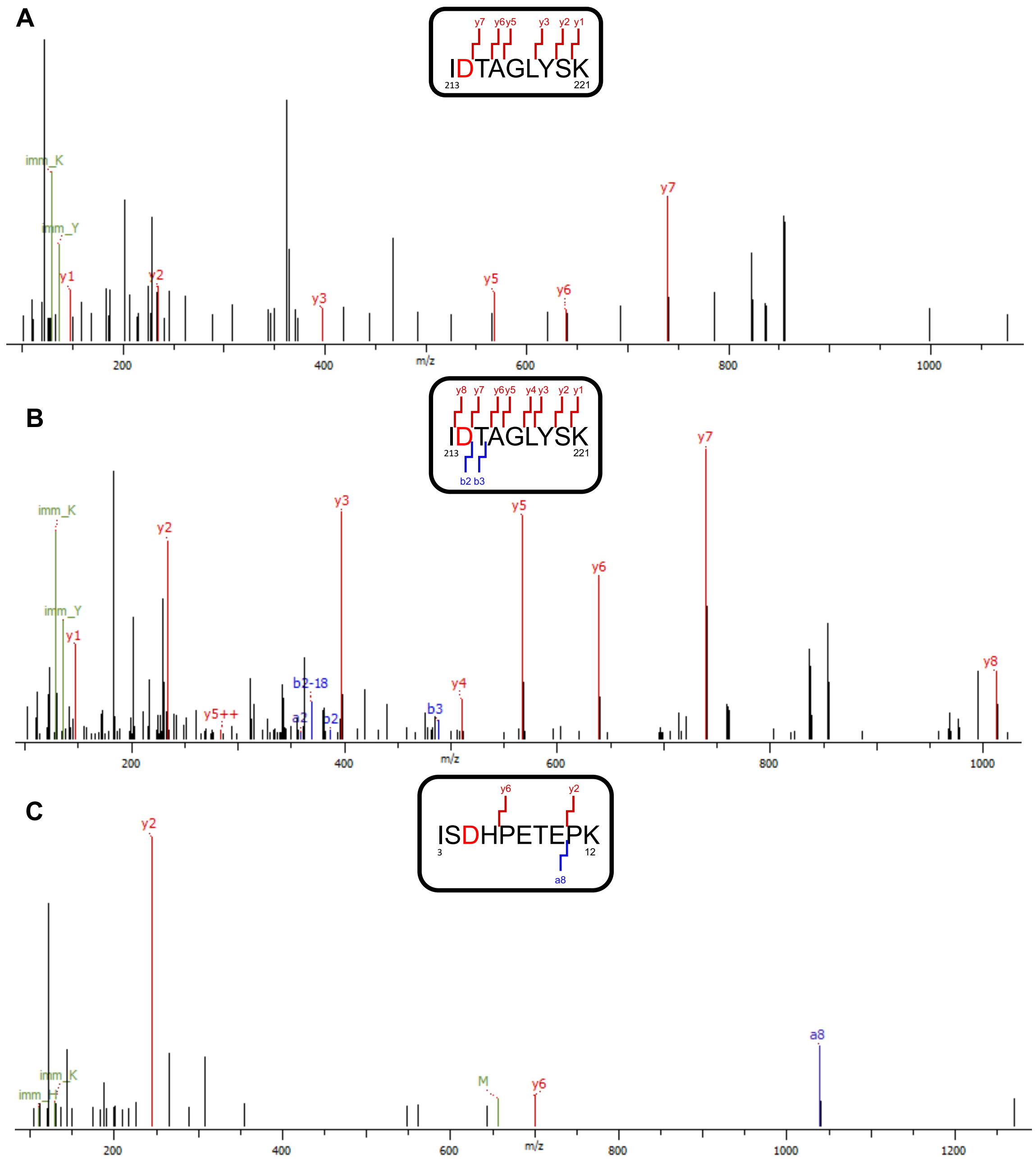

Figure S17. Electrospray ionization tandem spectrometry (MS/MS) daughter ion spectrum of streptol-labelled peptide after incubation of GH13 yeast α-glucosidase (G5003) with A) compound **1**, B) compound **2**, and C) compound **3** and trypsin mediated digestion. The peptide sequence is annotated with the modified amino acid highlighted in red and the demarcation of the daughter ion y and b.

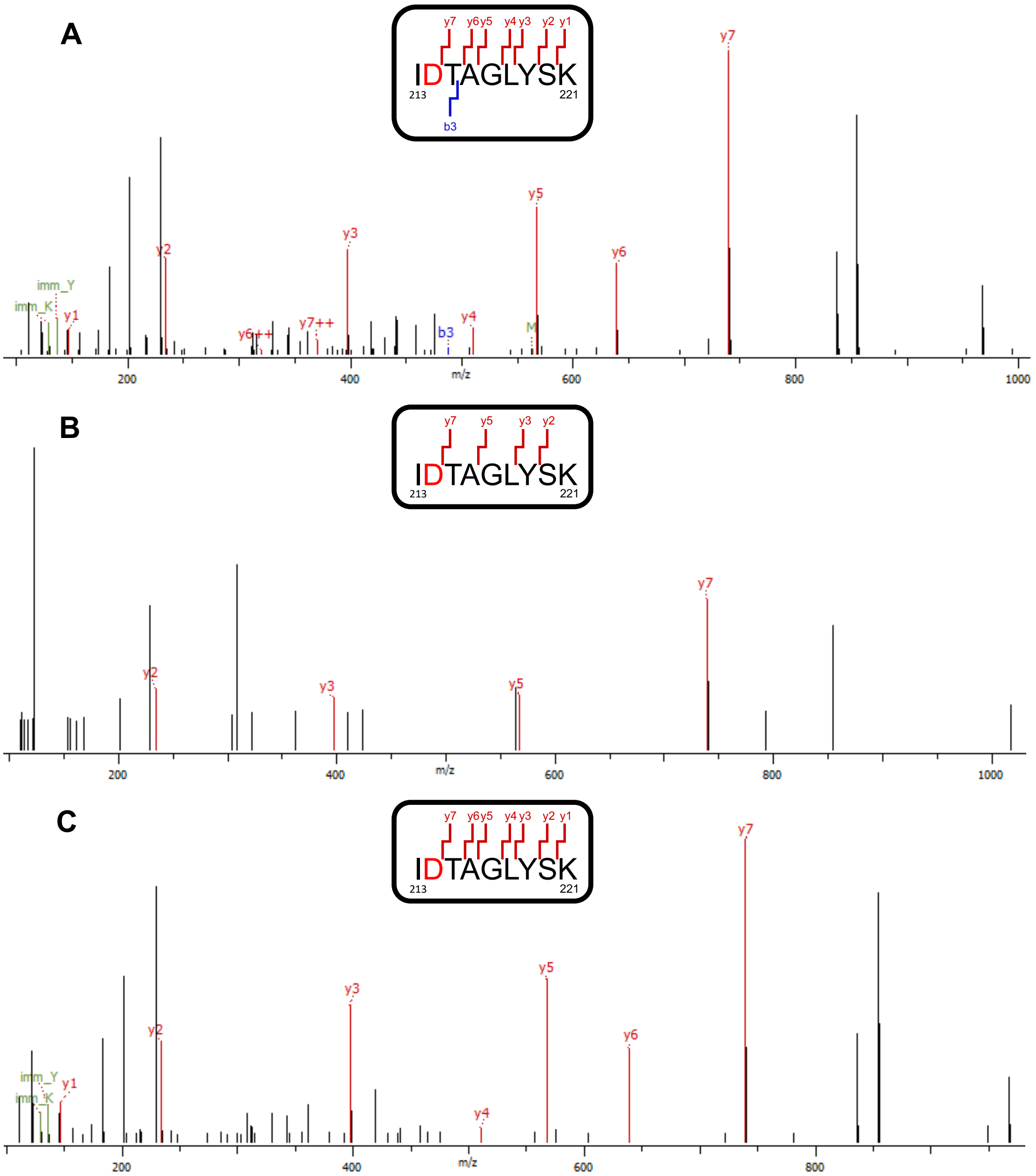

Figure S18. Electrospray ionization tandem spectrometry (MS/MS) daughter ion spectrum of streptol-labelled peptide 213-221 after incubation of GH13 yeast α-glucosidase (G0660) with A) compound **1**, B) compound **2**, and C) compound **3** and trypsin mediated digestion. The peptide sequence is annotated with the modified amino acid highlighted in red and the demarcation of the daughter ion y and b.

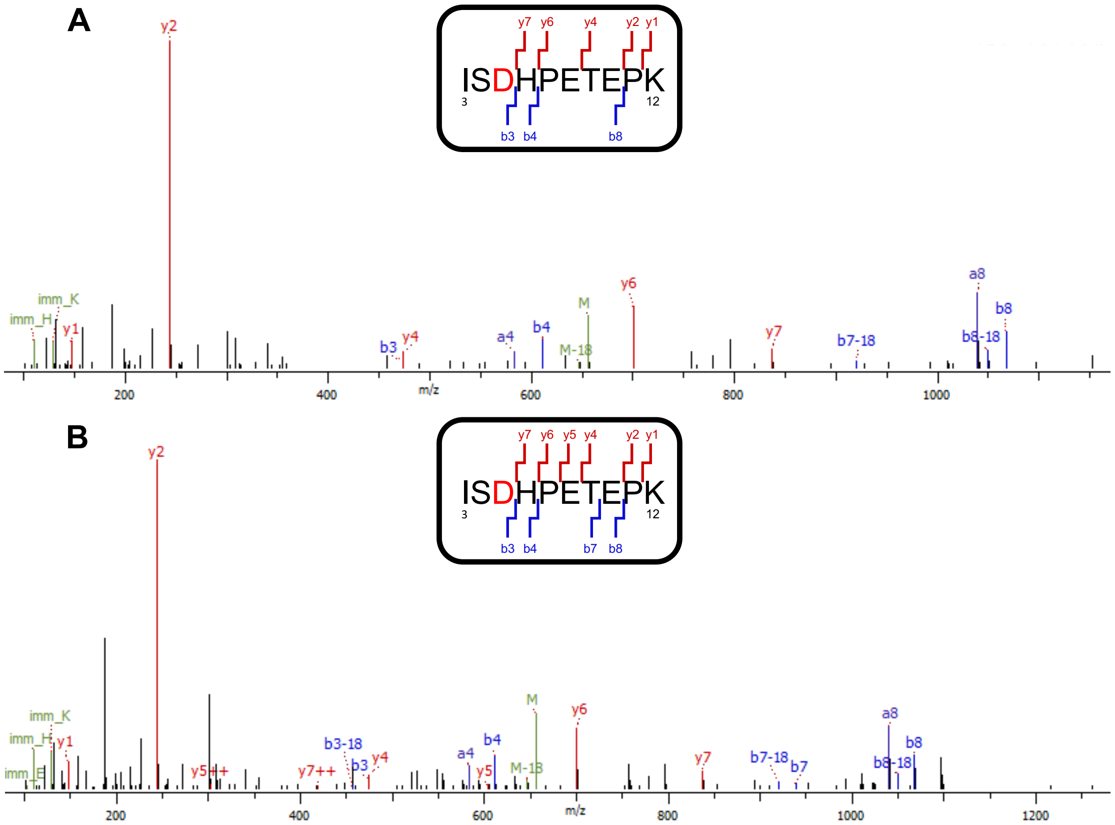

Figure S19. Electrospray ionization tandem spectrometry (MS/MS) daughter ion spectrum of streptol-labelled peptide 3-12 after incubation of GH13 yeast α-glucosidase (G0660) with A) compound **2**, B) compound **3** and trypsin mediated digestion. The peptide sequence is annotated with the modified amino acid highlighted in red and the demarcation of the daughter ion y and b.

Sequence alignment

Sequence alignment of isomaltase from *Saccharomyces cerevisiae* (Ima1p) and maltase from gene MAL32 from the same yeast (Mal32). The pairwise identity between these two sequences is 72%. The sequence is highlighted in brown, when it is the same amino-acid and not highlighted when different.

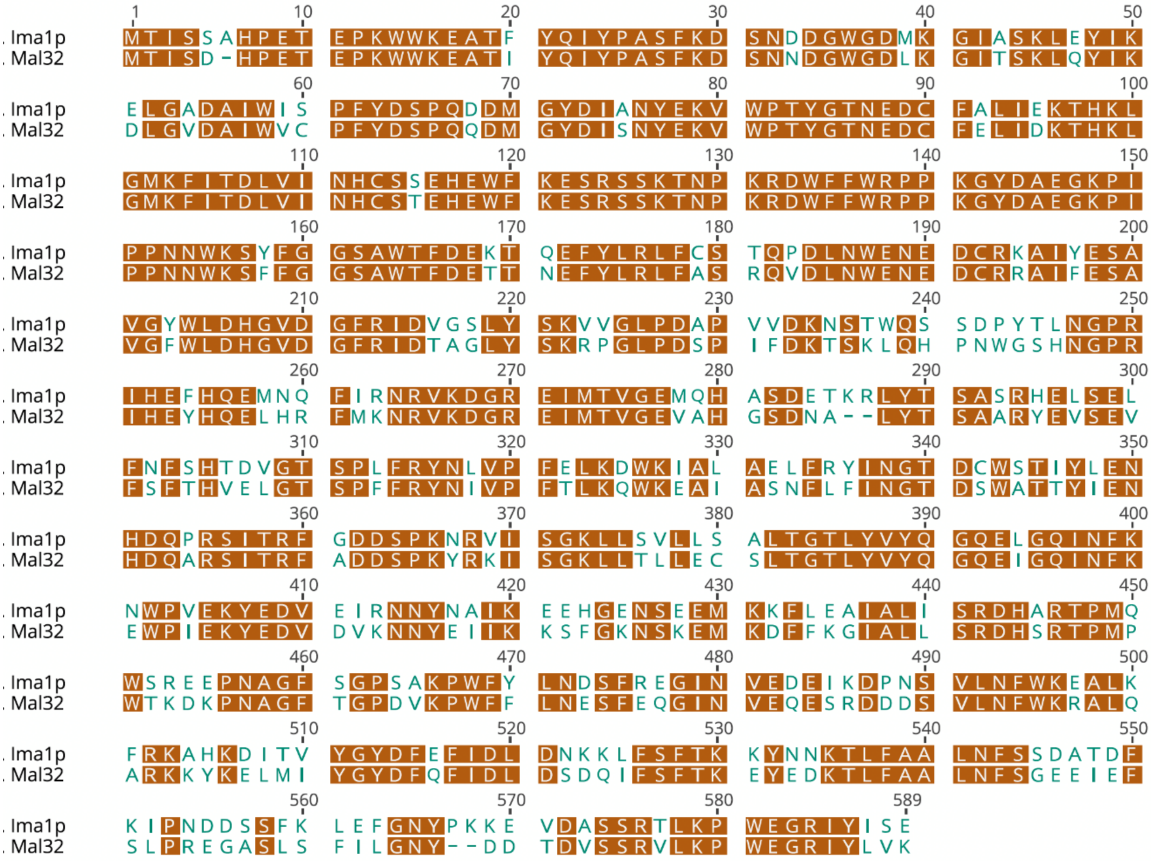

Figure S20. Alignment of the sequence of isomaltase and maltase of family GH13 from *Saccharomyces cerevisiae*.

Modeling and optimization of inhibitors using Moloc

Homology models were constructed from the α-glucosidase MAL32 sequence (Uniprot: P38158) using the SWISS-MODEL server,[3] supplying the structure of E277A isomaltase reported by Yamamoto *et al.* (PDB: 3AXH) as a template.[4] Phe157 was manually adjusted in Moloc (Gerber Molecular Design)[5] to match TYR157 of known hydrolase crystal structures. Streptol and its derivatives were modelled in Moloc on the basis of the glucose fragment from E277A mutant isomaltase reported by Yamamoto *et al.* (PDB: 3AXH). The structures were posed manually and then minimised using the MAB force field. Visualizations were created in PyMOL 2.3.2 (Schrödinger LLC.).

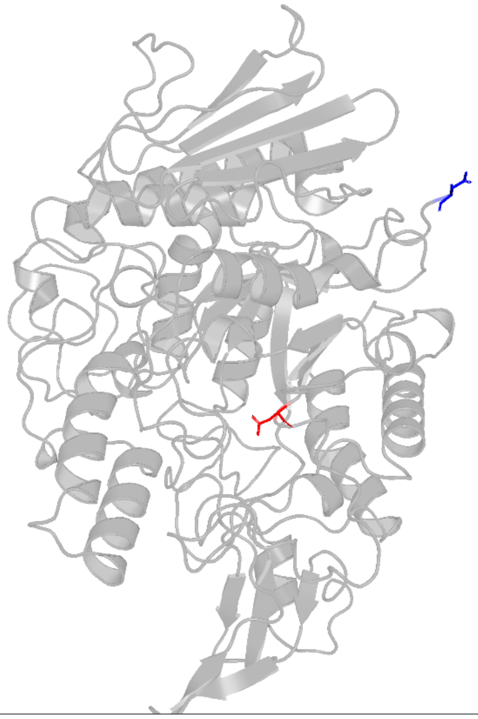

Figure S21. Homology model of maltase issued from gene MAL32 of *S. cerevisiae* built from the crystal structure of mutant isomaltase of *S. cerevisiae* with the aspartic acid 5 highlighted in blue and aspartic acid 214 in red.

Computational details for the DFT calculations

All DFT calculations were performed with the ORCA 5.0.1 package.[6,7] Prior to geometry optimization, all the putative structures were subjected to conformational search using the GFN2-xTB method[8] implemented in the CREST/xTB software package.[9] Geometry optimizations were performed at the r^2SCAN-3c level of theory.[10] Numerical frequency calculations were performed at the same level of theory to characterize all stationary points as either intermediates (no imaginary frequencies) or transition states (exactly one imaginary frequency). The electronic energy was further refined through single point calculations at the ωB97M-V level of theory.[11] All the atoms were described with def2-TZVPP basis set. Solvent

effects were taken into consideration through the use of the CPCM solvation model for water, as implemented in ORCA.[12] Gibbs energies were corrected to the standard state (298 K, 1 M). The optimized structures were visualized with the CYLview software.

Table S2. Computational results

Geometry	E(r^2SCAN-3c)	G_{corr}(r^2SCAN-3c)	E(ωB97M-V)	G(ωB97M-V)	IF
1	-1289.9678	0.2311	-1290.2808	-1290.0467	-
1cc	-573.9480	0.1417	-574.0755	-573.9308	-
1cc-H	-574.7626	0.1554	-574.8942	-574.7357	-
1TS	-1479.2411	0.2446	-1479.5886	-1479.3410	-293.05
1f	-763.3077	0.1658	-763.4944	-763.3257	-
2	-1289.9652	0.2308	-1290.2781	-1290.0442	-
2cc	-573.9288	0.1425	-574.0606	-573.9151	-
2cc-H	-574.7640	0.1546	-574.8957	-574.7381	-
2cc-H'	-574.7625	0.1560	-574.8946	-574.7356	-
2TS	-1479.2362	0.2444	-1479.5841	-1479.3367	-416.70
2f	-763.3067	0.1653	-763.4934	-763.3251	-
3	-1289.9620	0.2302	-1290.2754	-1290.0421	-
3cc	-573.9351	0.1427	-574.0614	-573.9157	-
3cc-H	-574.7631	0.1546	-574.8945	-574.7369	-
3cc-H'	-574.7624	0.1548	-574.8931	-574.7353	-
3TS	-1479.2353	0.2455	-1479.5838	-1479.3353	-323.82
3f	-763.3068	0.1653	-763.4934	-763.3252	-
H-	-0.6182	-0.0100	-0.6468	-0.6538	-
DNPO-	-715.9654	0.0595	-716.1338	-716.0713	-
HCOO-	-189.2898	-0.0036	-189.3378	-189.3384	-

References

(1) Mehta, G.; Pujar, S. R.; Ramesh, S. S.; Islam, K. Enantioselective Total Synthesis of Polyoxygenated Cyclohexanoids: (+)-Streptol, Ent-RKTS-33 and Putative '(+)-Parasitenone'. Identity of Parasitenone with (+)-Epoxydon. *Tetrahedron Lett.* **2005**, *46* (19), 3373–3376. https://doi.org/10.1016/j.tetlet.2005.03.087.

(2) Abadi, S. S. K.; Tran, M.; Yadav, A. K.; Adabala, P. J. P.; Chakladar, S.; Bennet, A. J. New Class of Glycoside Hydrolase Mechanism-Based Covalent Inhibitors: Glycosylation Transition State Conformations. *J. Am. Chem. Soc.* **2017**, *139* (4), 10625–10628. https://doi.org/10.1021/jacs.7b05065

(3) Waterhouse, A.; Bertoni, M.; Bienert, S.; Studer, G.; Tauriello, G.; Gumienny, R.; Heer, F. T.; de Beer, T. A. P.; Rempfer, C.; Bordoli, L.; Lepore, R.; Schwede, T. SWISS-MODEL: Homology Modelling of Protein Structures and Complexes. *Nucleic Acids Res.* **2018**, *46* (W1), W296–W303. https://doi.org/10.1093/nar/gky427.

(4) Yamamoto, K.; Miyake, H.; Kusunoki, M.; Osaki, S. Steric Hindrance by 2 Amino Acid Residues Determines the Substrate Specificity of Isomaltase from Saccharomyces Cerevisiae. *J. Biosci. Bioeng.* **2011**, *112* (6), 545–550. https://doi.org/10.1016/j.jbiosc.2011.08.016.

(5) Gerber, P. R.; Müller, K. MAB, a Generally Applicable Molecular Force Field for Structure Modelling in Medicinal Chemistry. *J. Comput. Aided Mol. Des.* **1995**, *9* (3), 251–268. https://doi.org/10.1007/BF00124456.

(6) Neese, F. The ORCA Program System. *WIREs Comput. Mol. Sci.* **2012**, *2* (1), 73–78. https://doi.org/10.1002/wcms.81.

(7) Neese, F. Software Update: The ORCA Program System, Version 4.0. *WIREs Comput. Mol. Sci.* **2018**, *8* (1), e1327. https://doi.org/10.1002/wcms.1327.

(8) Grimme, S.; Bannwarth, C.; Shushkov, P. A Robust and Accurate Tight-Binding Quantum Chemical Method for Structures, Vibrational Frequencies, and Noncovalent Interactions of Large Molecular Systems Parametrized for All Spd-Block Elements (Z = 1–86). *J. Chem. Theory Comput.* **2017**, *13* (5), 1989–2009. https://doi.org/10.1021/acs.jctc.7b00118.

(9) Pracht, P.; Bohle, F.; Grimme, S. Automated Exploration of the Low-Energy Chemical Space with Fast Quantum Chemical Methods. *Phys. Chem. Chem. Phys.* **2020**, *22* (14), 7169–7192. https://doi.org/10.1039/C9CP06869D.

(10) Grimme, S.; Hansen, A.; Ehlert, S.; Mewes, J.-M. r^2 SCAN-3c: A "Swiss Army Knife" Composite Electronic-Structure Method. *J. Chem. Phys.* **2021**, *154* (6), 064103. https://doi.org/10.1063/5.0040021.

(11) Mardirossian, N.; Head-Gordon, M. ωB97M-V: A Combinatorially Optimized, Range-Separated Hybrid, Meta-GGA Density Functional with VV10 Nonlocal Correlation. *J. Chem. Phys.* **2016**, *144* (21), 214110. https://doi.org/10.1063/1.4952647.

(12) Barone, V.; Cossi, M. Quantum Calculation of Molecular Energies and Energy Gradients in Solution by a Conductor Solvent Model. *J. Phys. Chem. A* **1998**, *102* (11), 1995–2001. https://doi.org/10.1021/jp9716997.

NMR spectra

Synthesis of inhibitors **1** and **1'**

^{1}H NMR spectrum of compound **4**

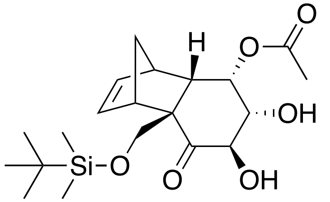

4

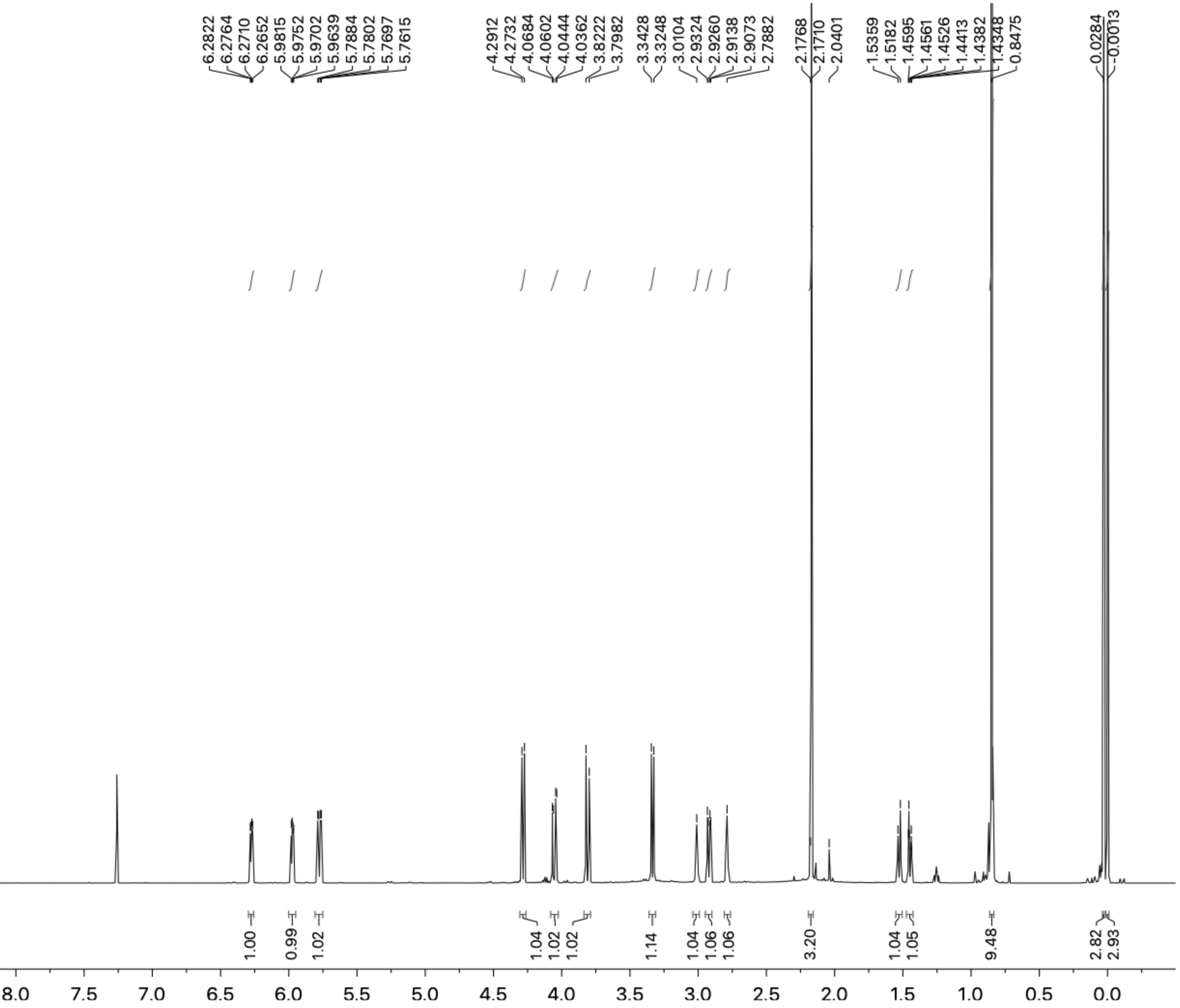

^{13}C NMR spectrum of compound **4**

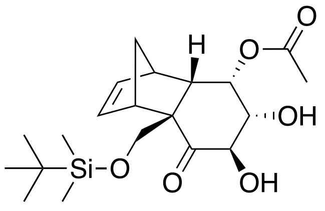

4

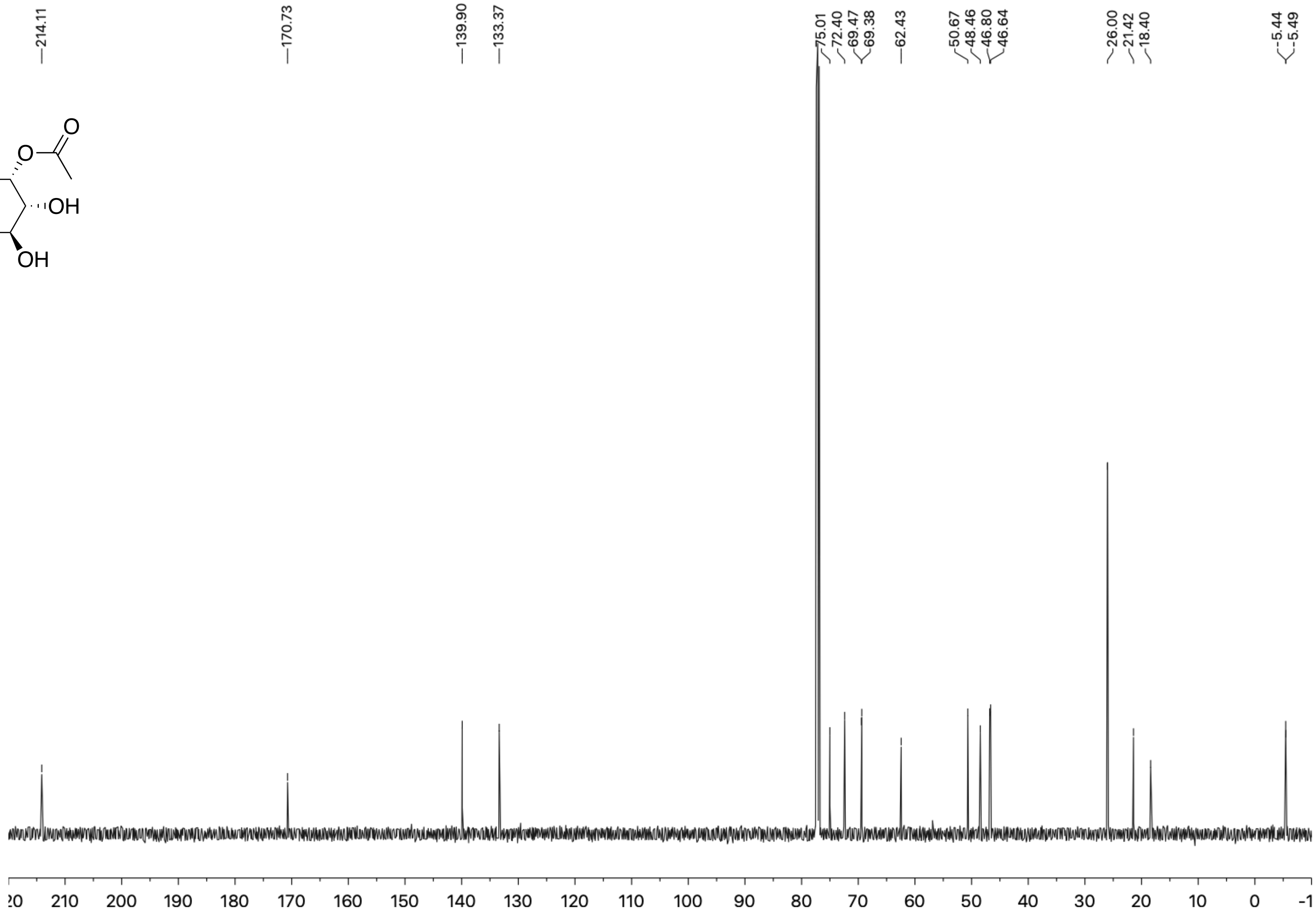

^{1}H NMR spectrum of compound **16**

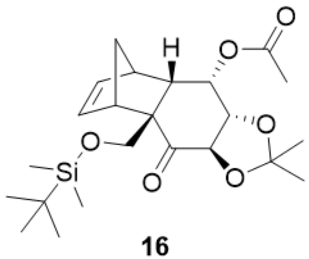

16

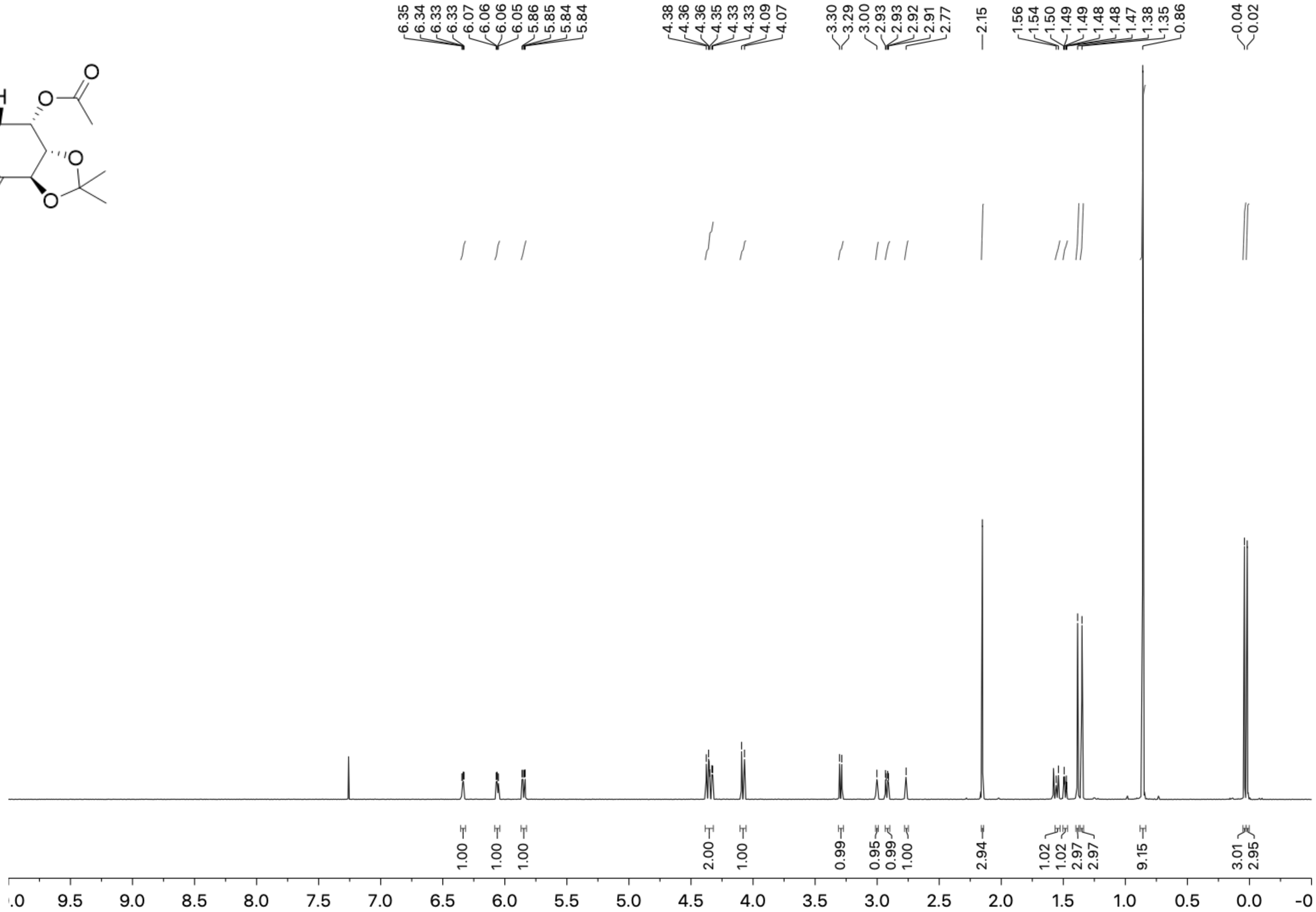

^{13}C NMR spectrum of compound **16**

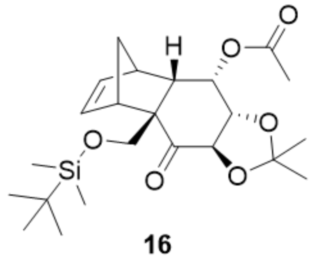

16

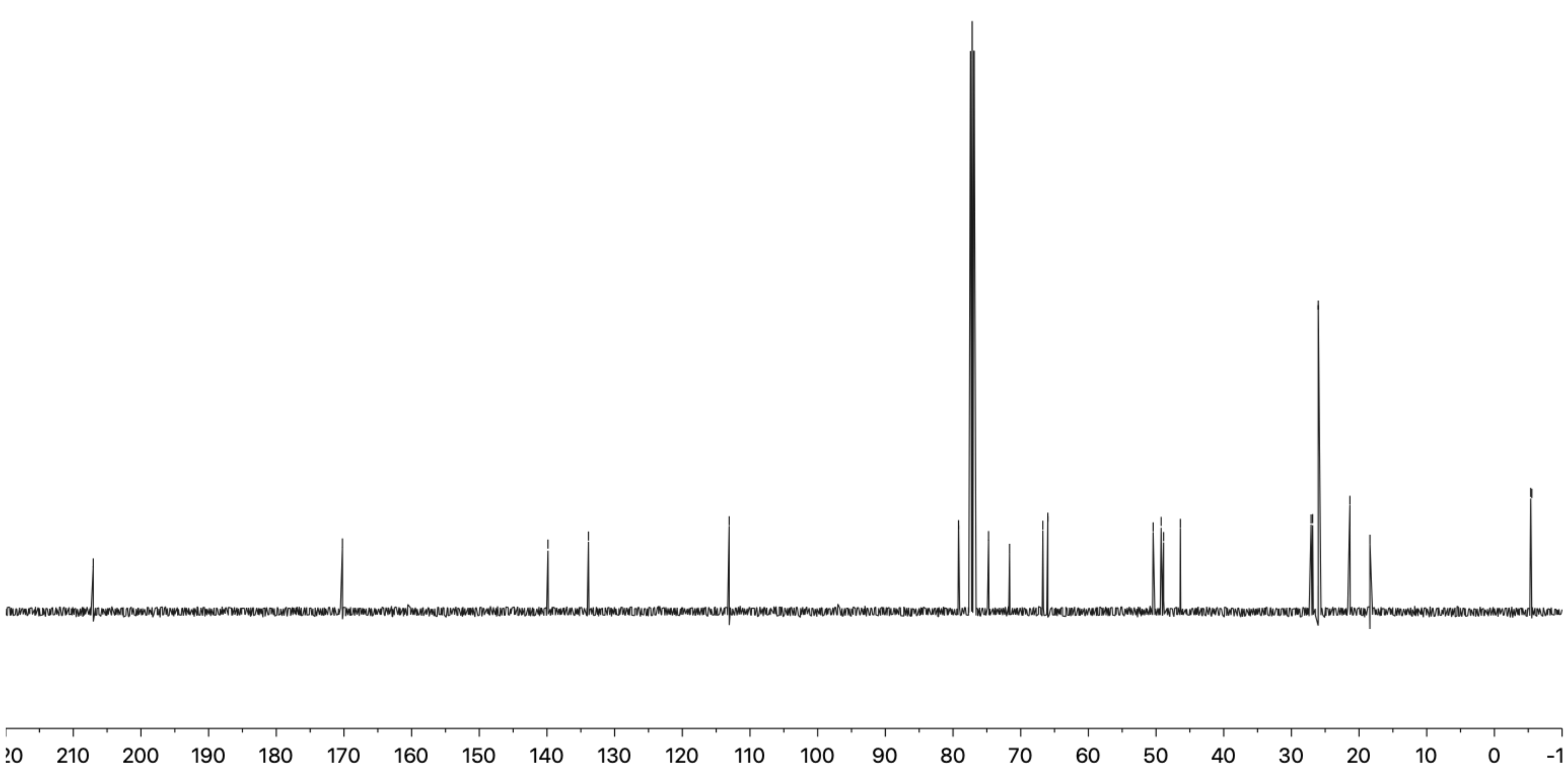

^{1}H NMR spectrum of compound **17**

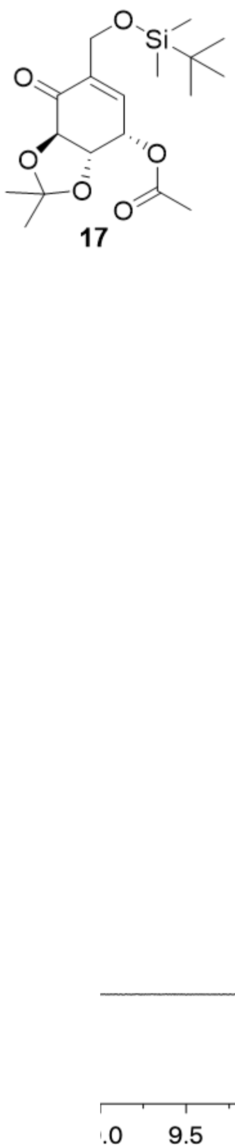

17

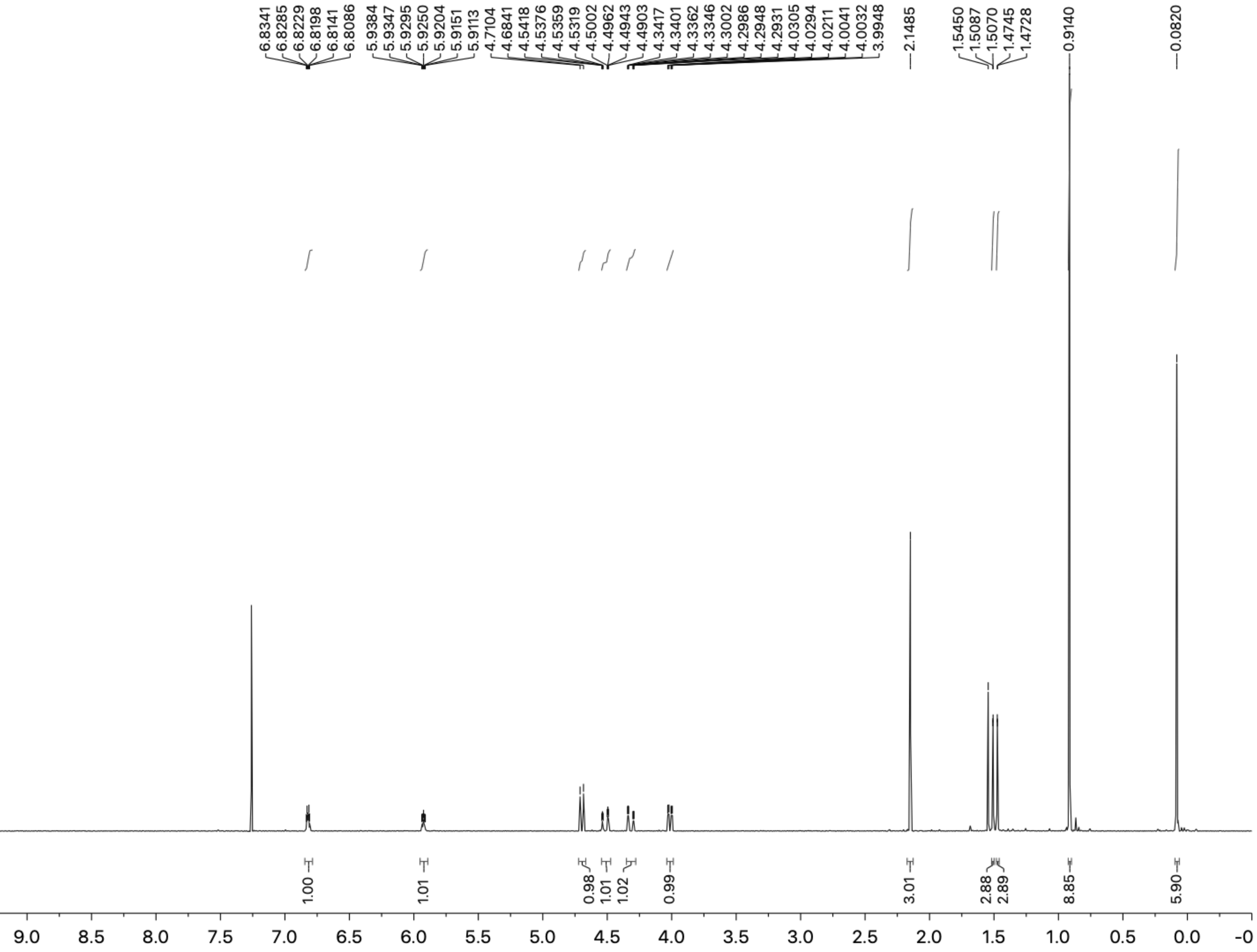

^{13}C NMR spectrum of compound **17**

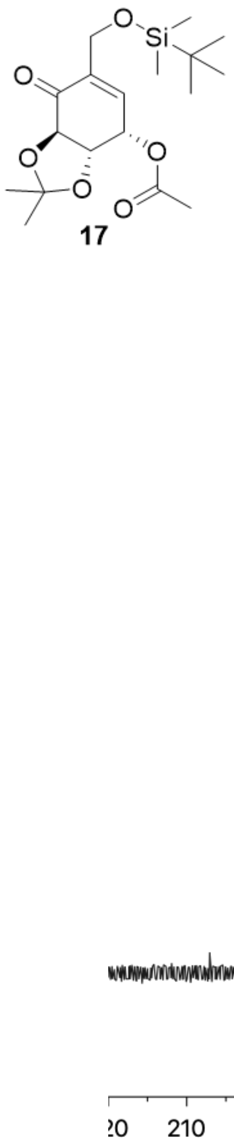

17

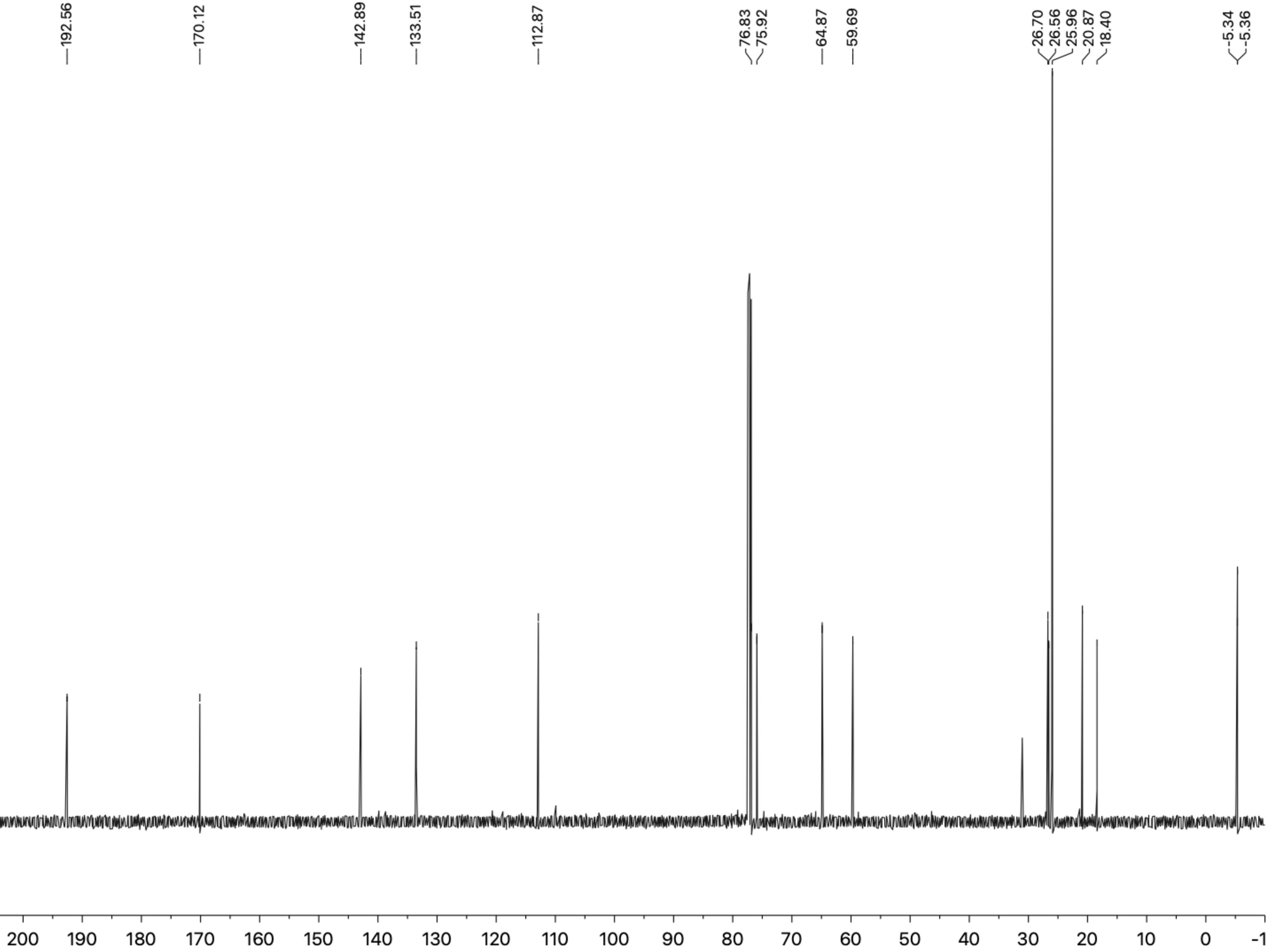

^{1}H NMR spectrum of compound **5**

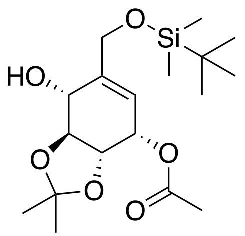

5

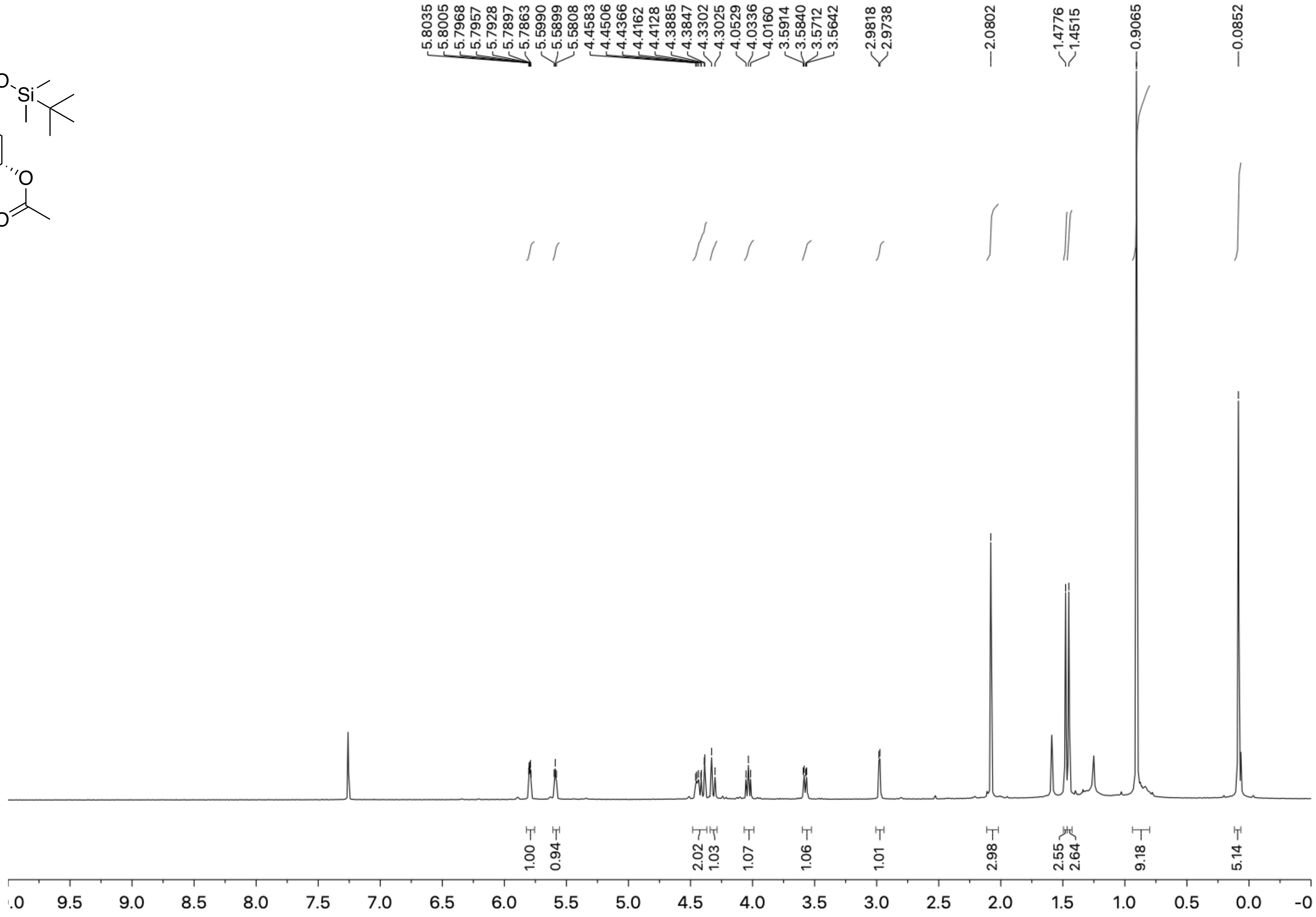

^{13}C NMR Spectrum of compound **5**

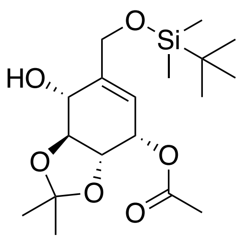

5

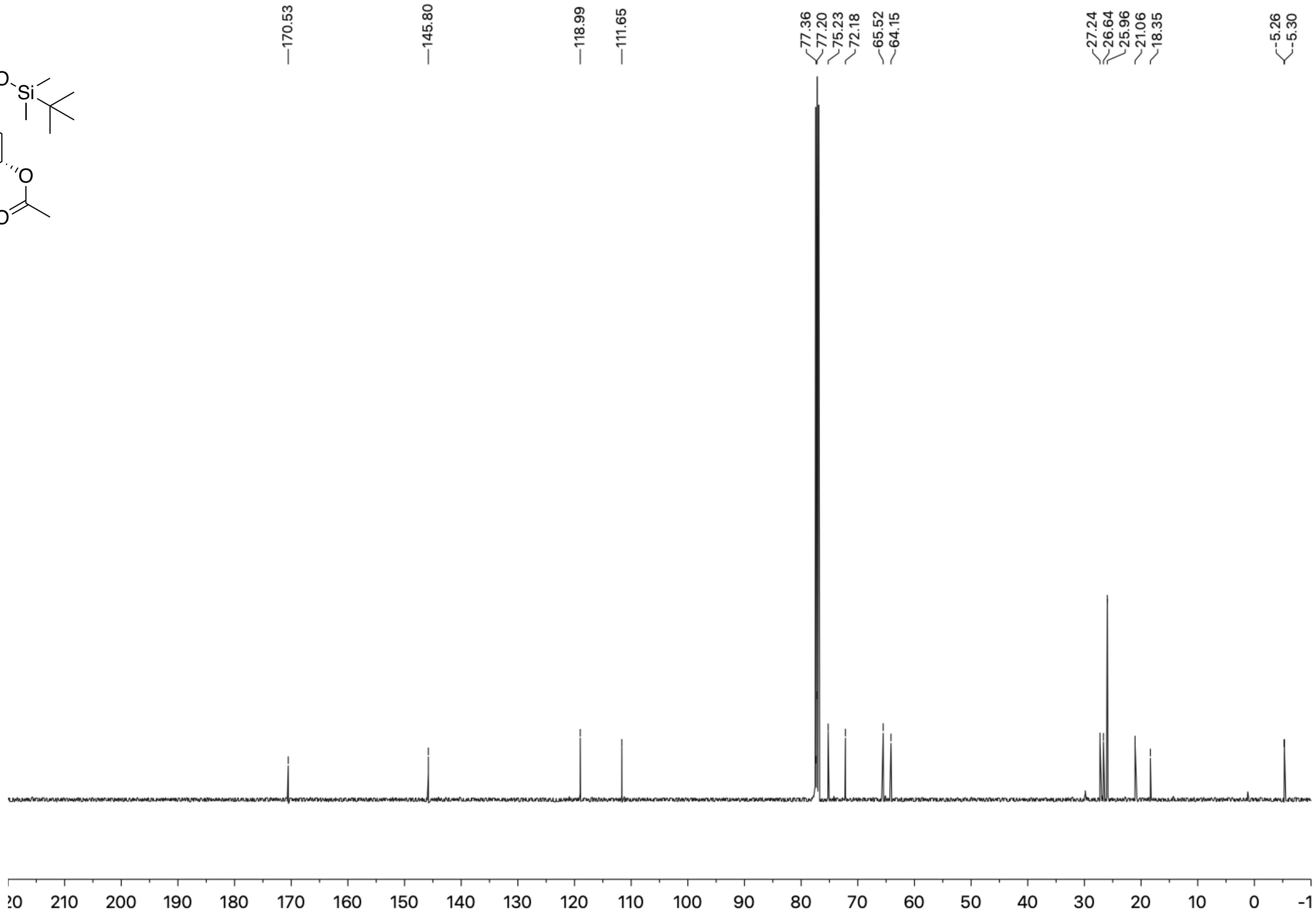

^{1}H NMR spectum of compound **6**

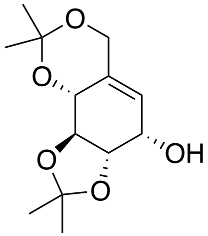

6

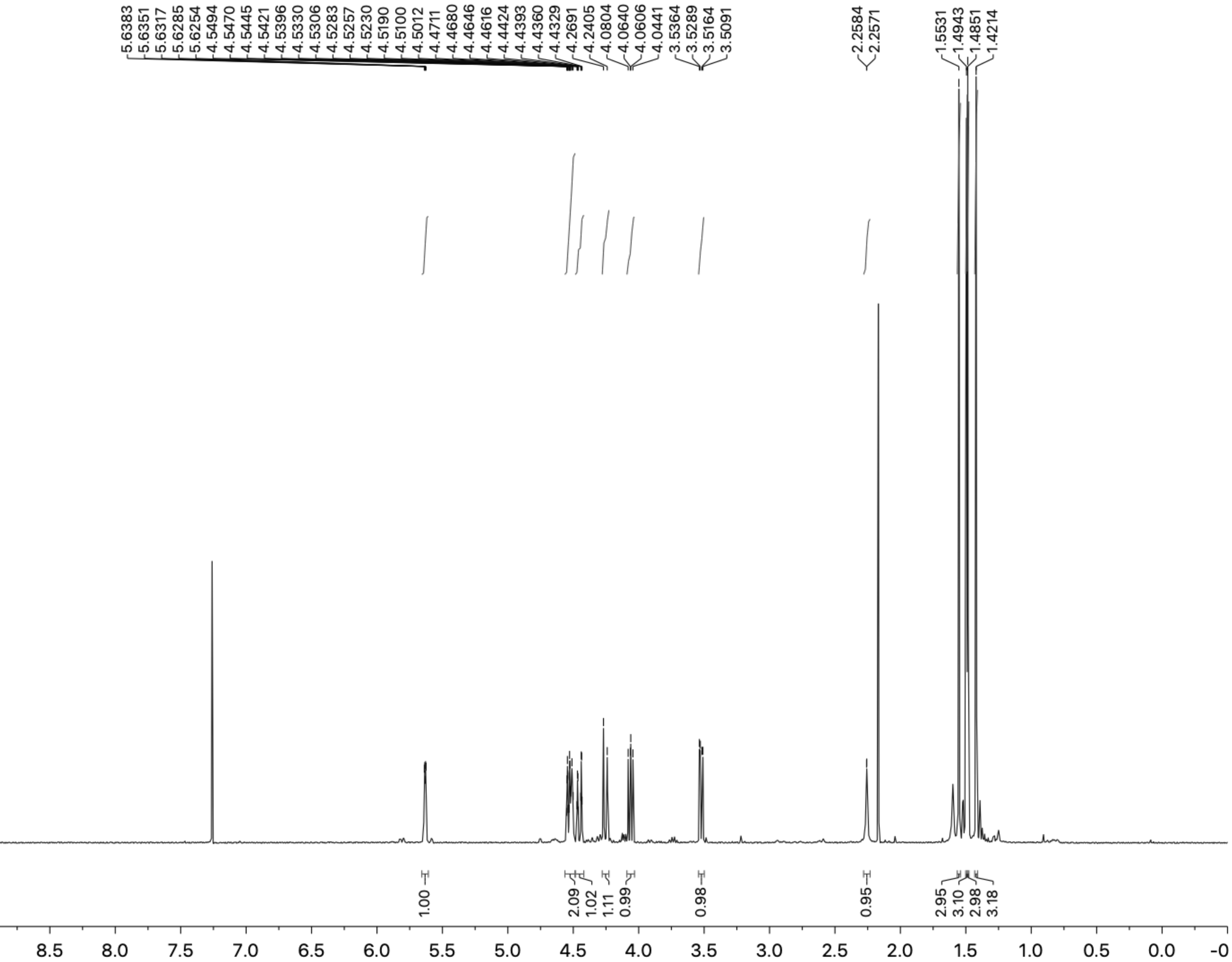

^{13}C NMR spectrum of compound **6**

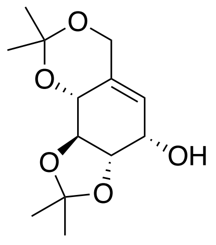

6

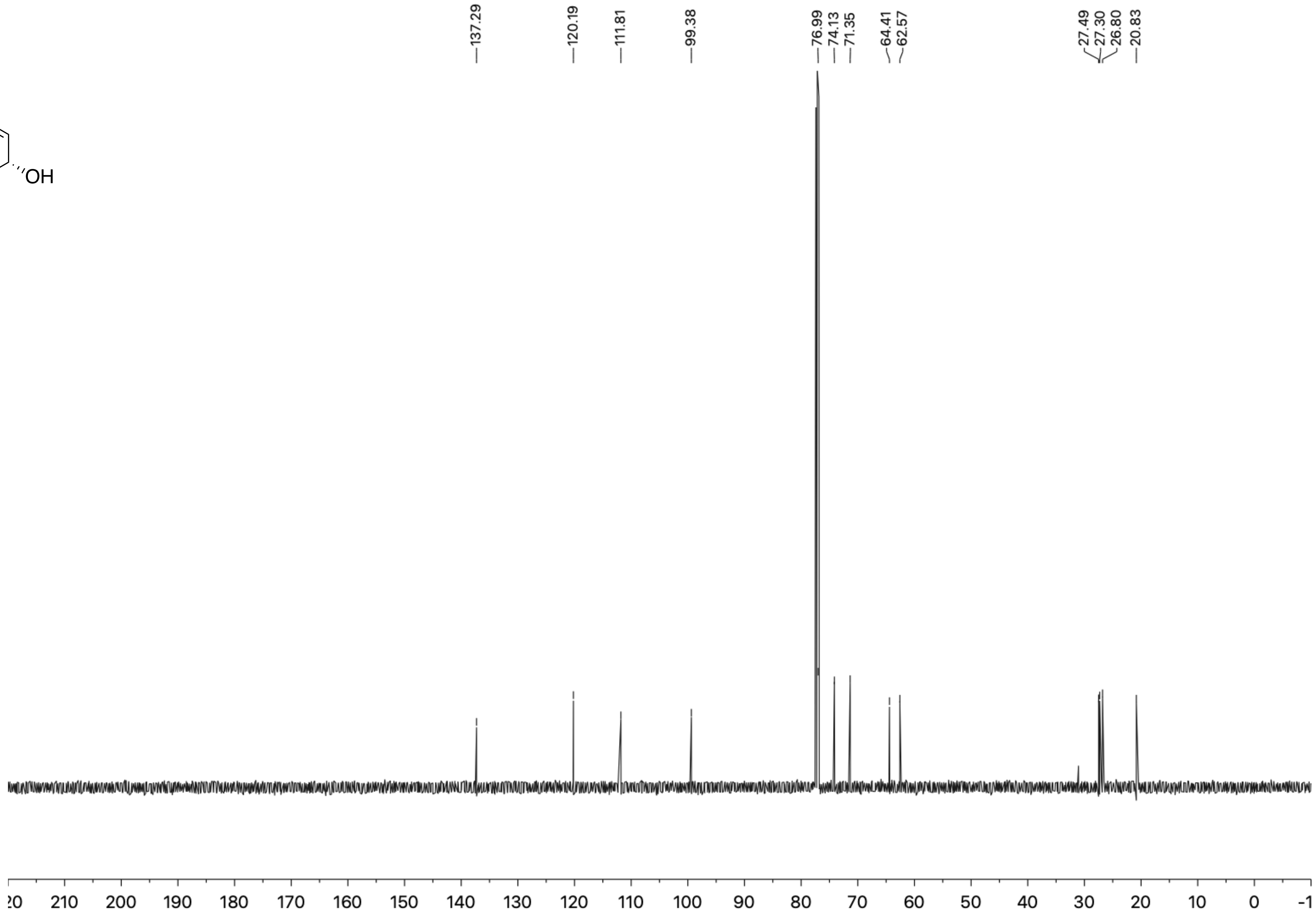

^{1}H NMR spectrum of compound **18**

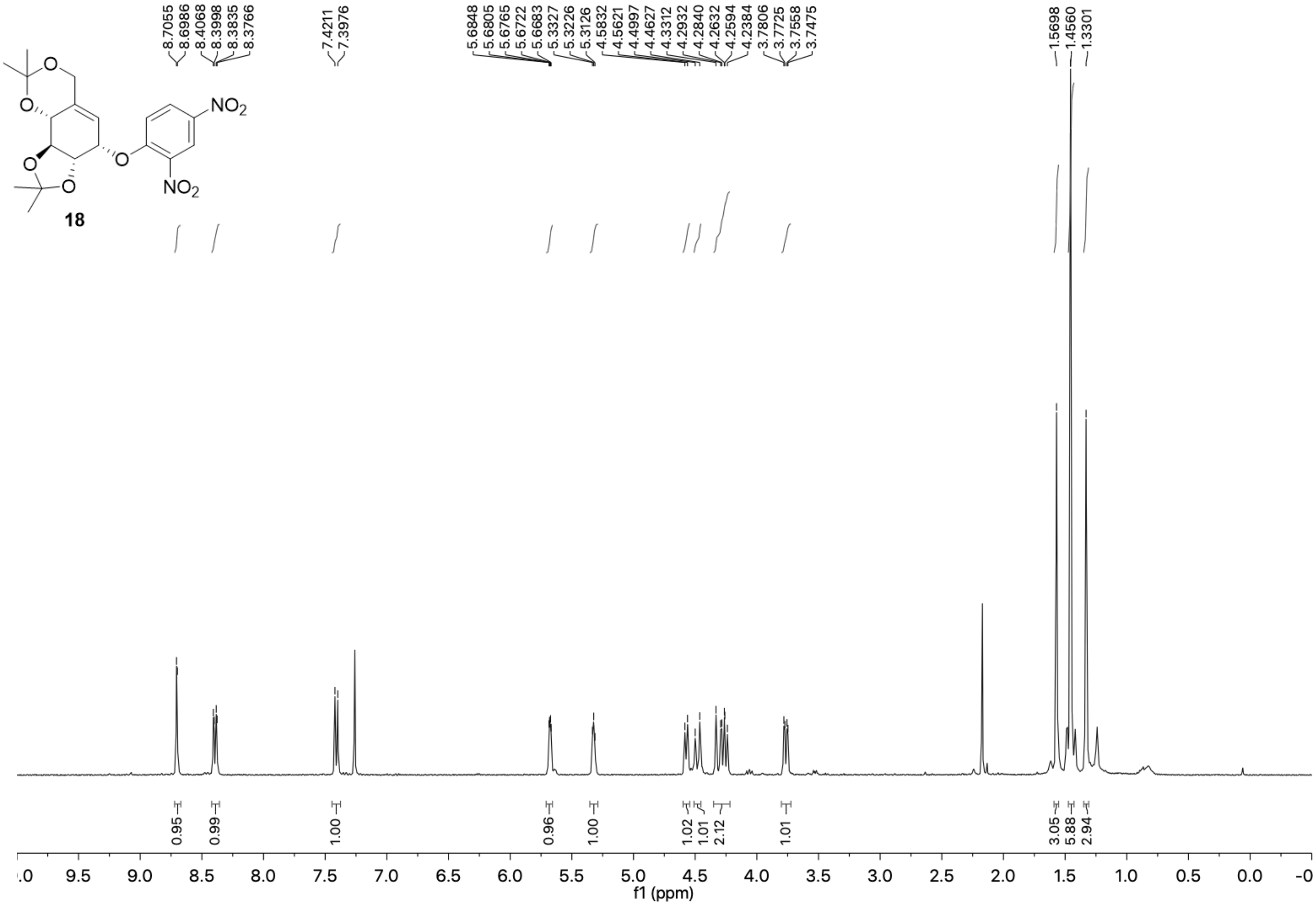

^{13}C NMR Spectrum of compound **18**

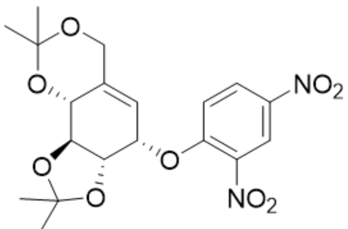

18

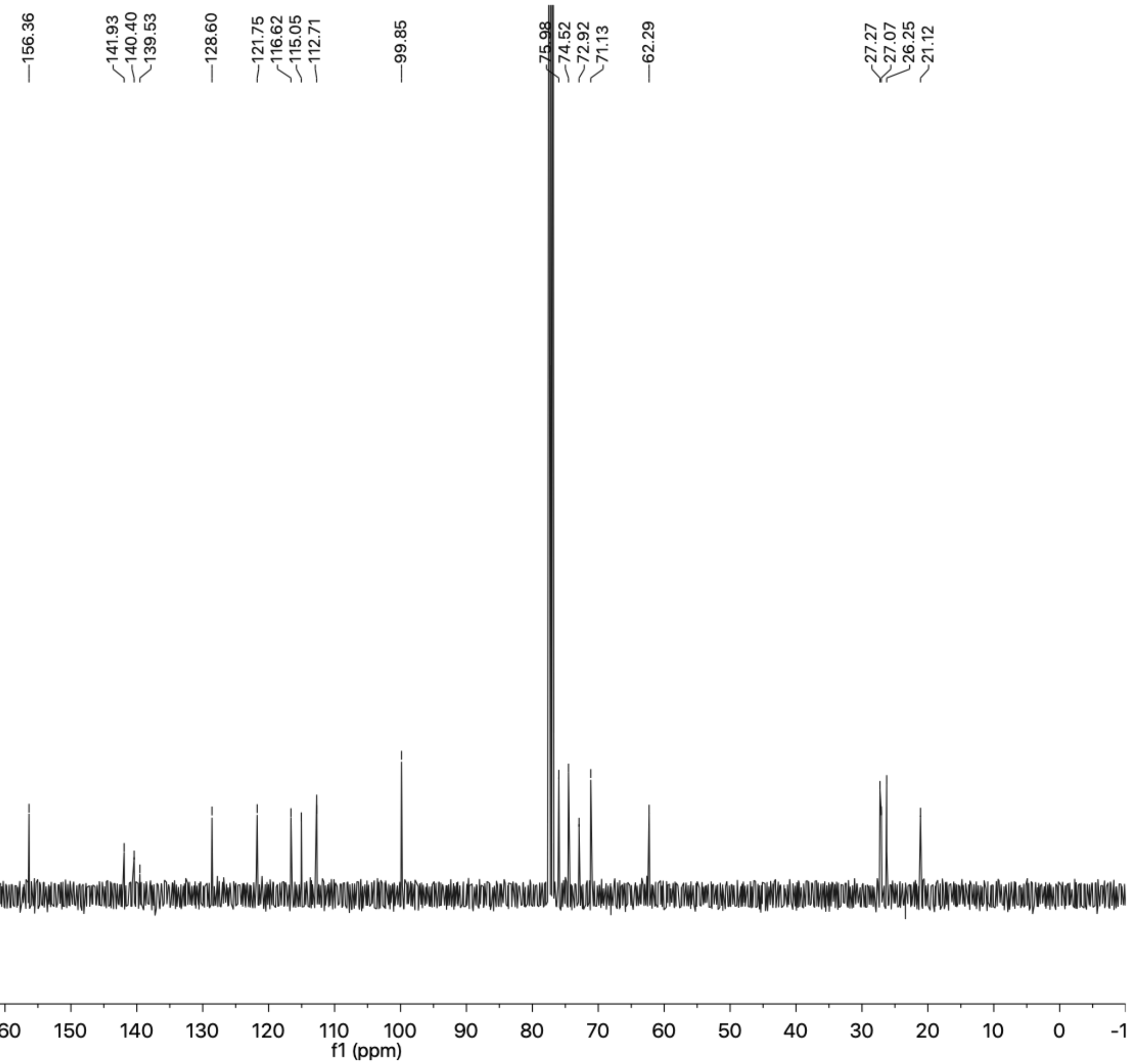

^{1}H NMR Spectrum of compound **1**

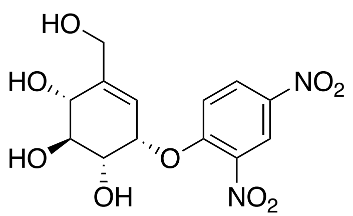

1

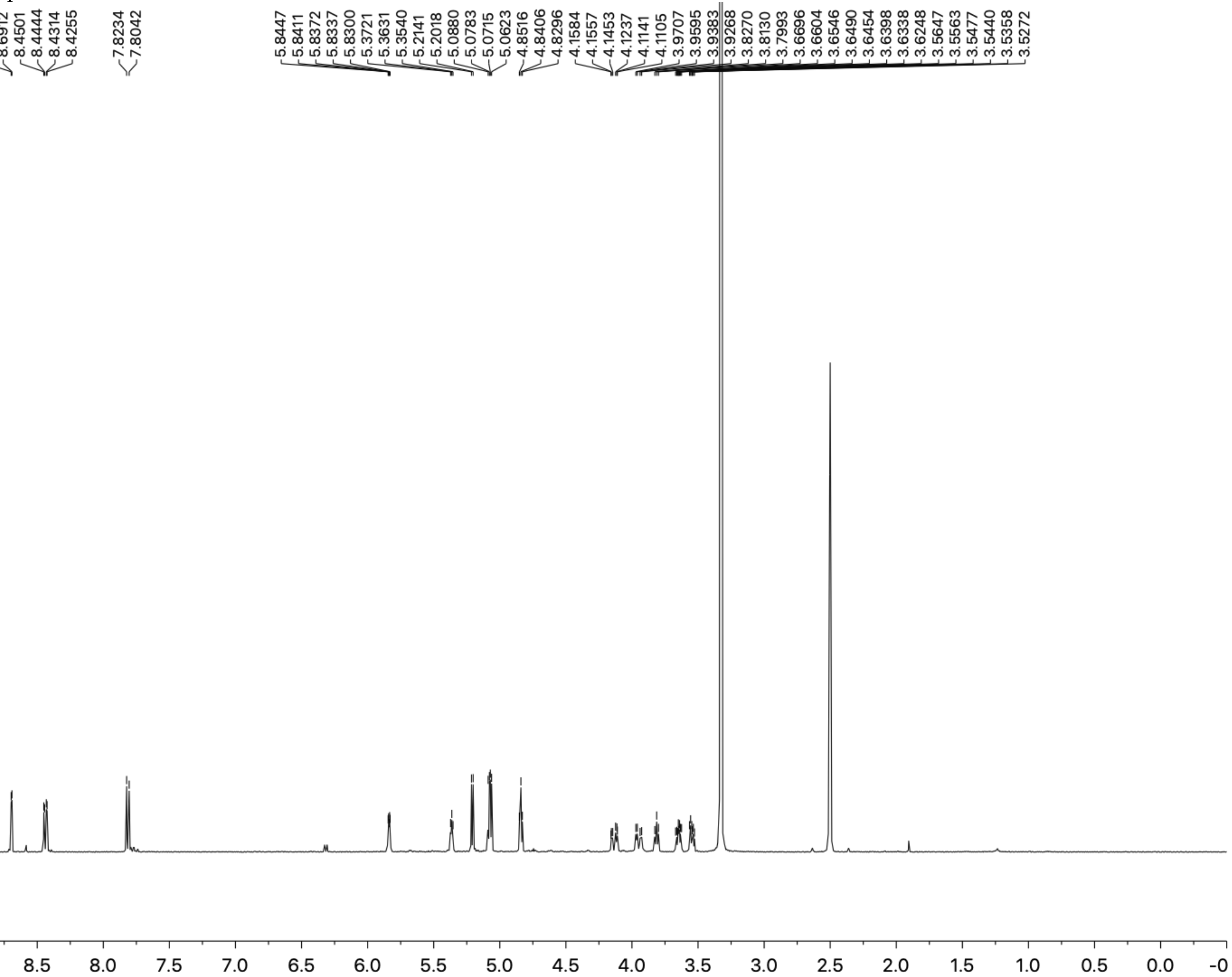

^{13}C spectrum of compound **1**

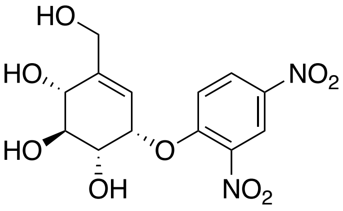

1

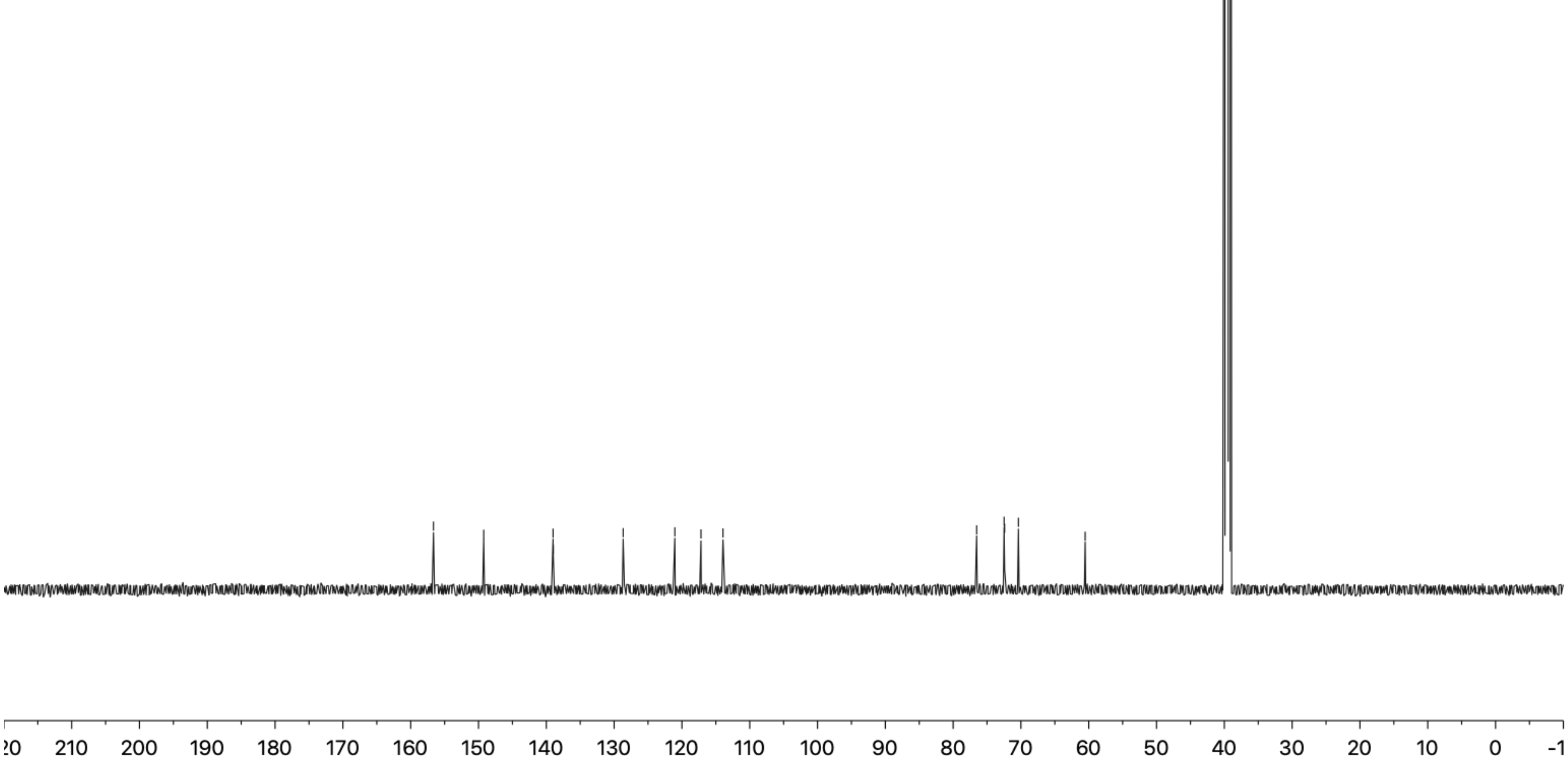

^{1}H NMR spectrum of compound **19**

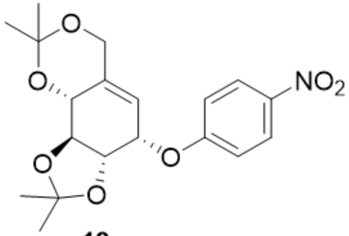

19

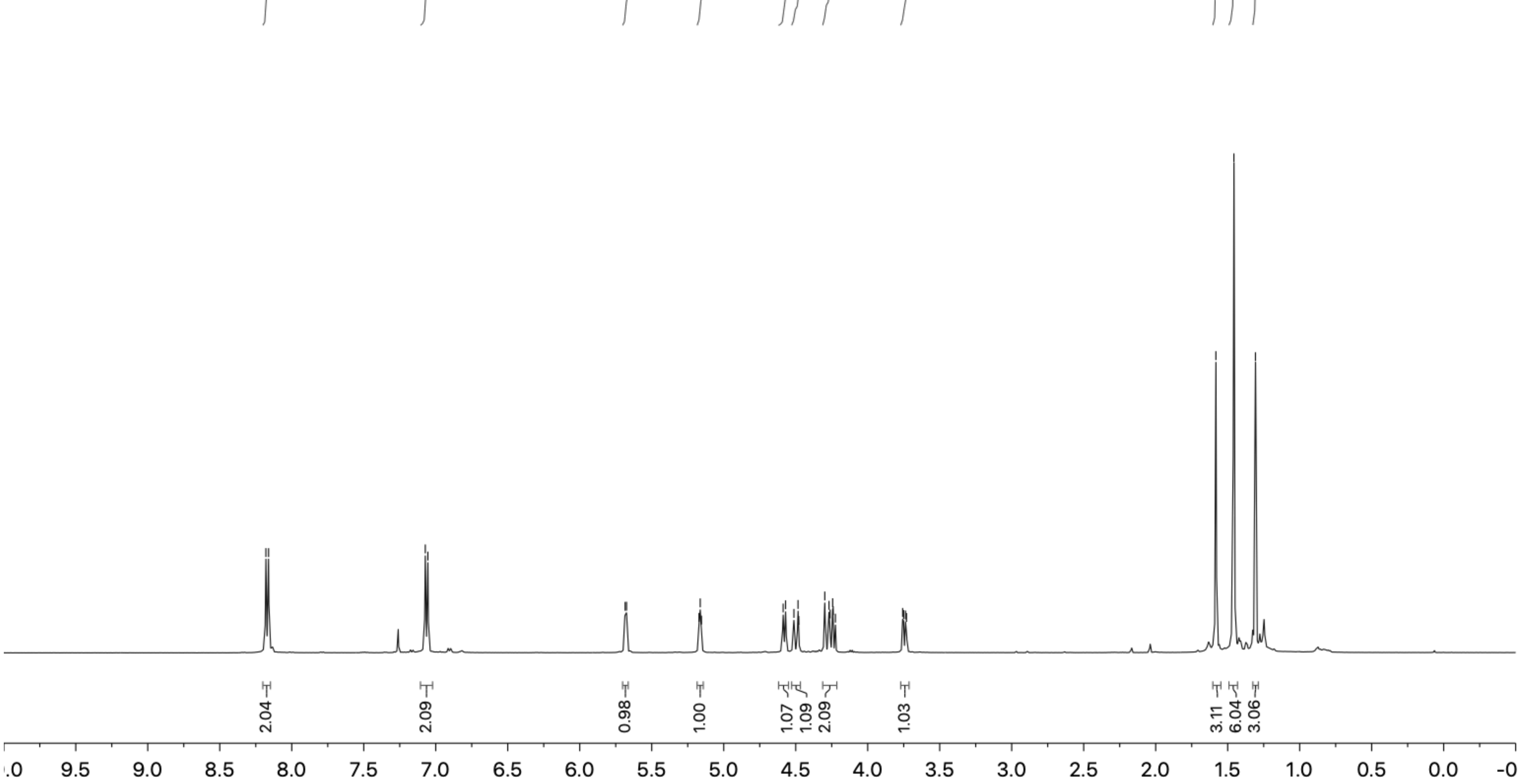

^{13}C NMR spectrum of compound **19**

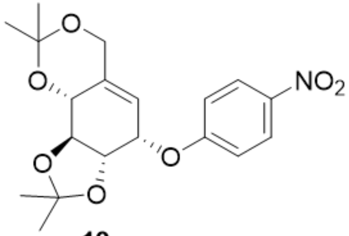

19

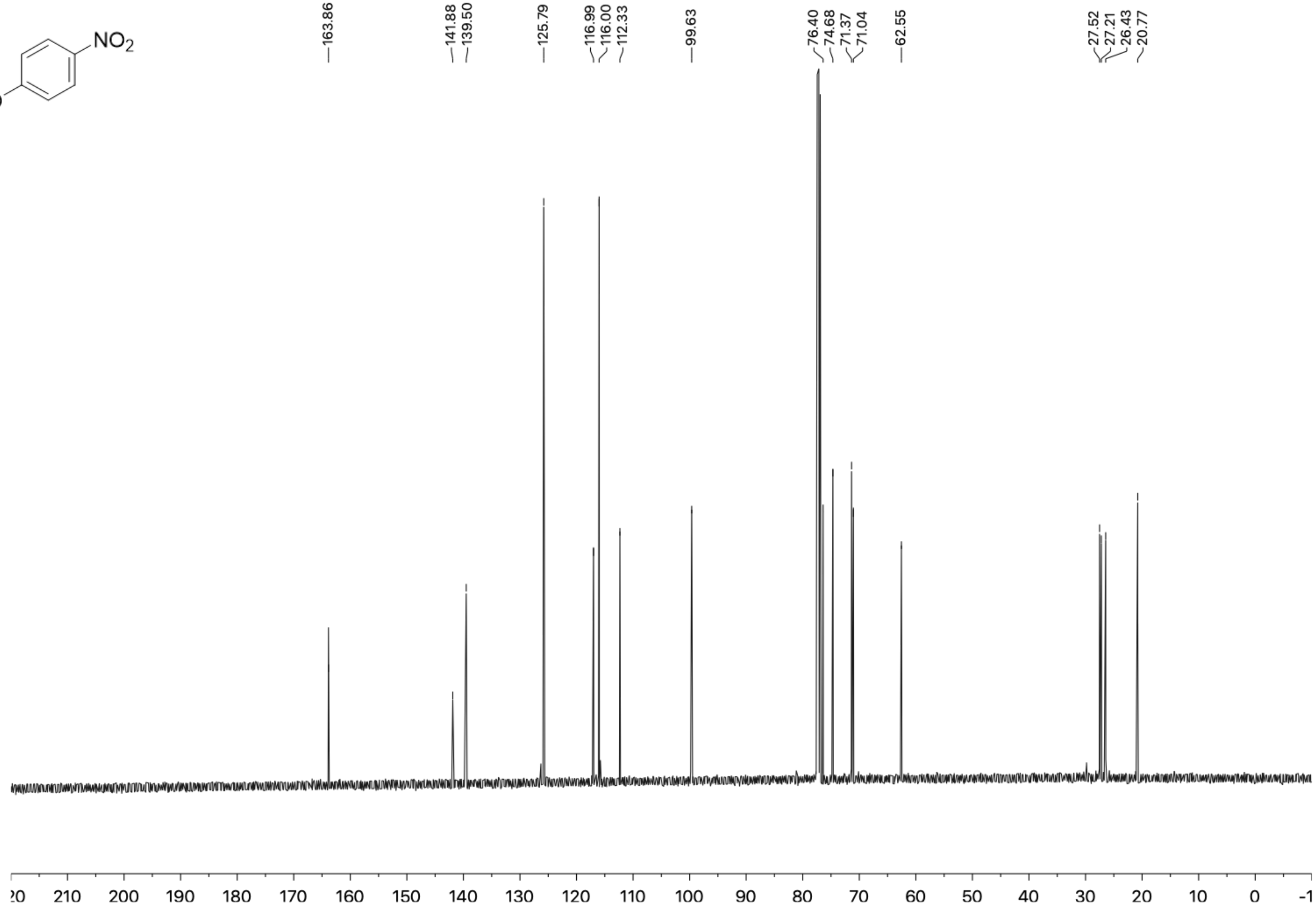

^{1}H NMR spectrum of compound **1'**

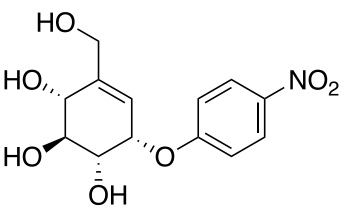

1'

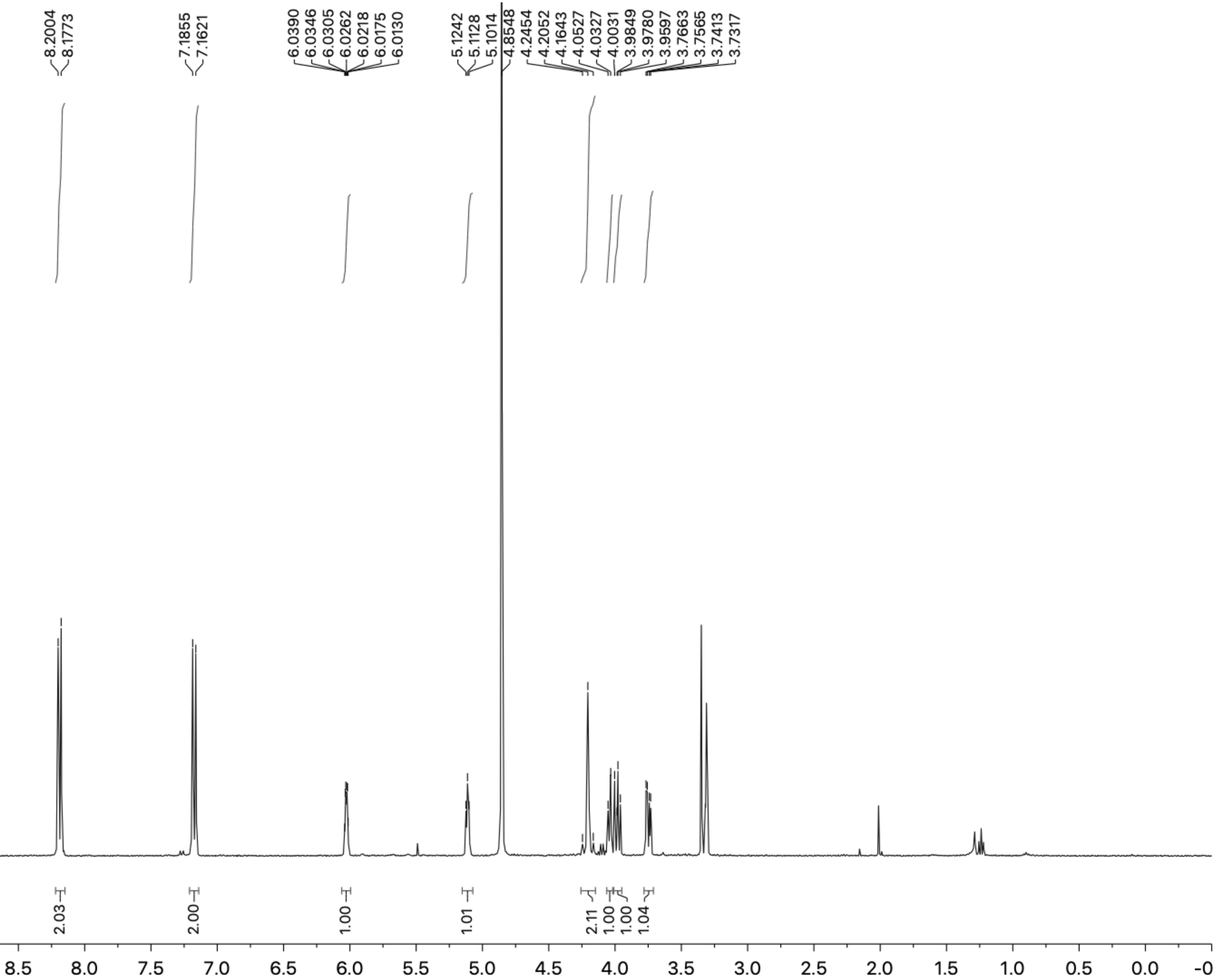

^{13}C NMR spectrum of compound **1’**

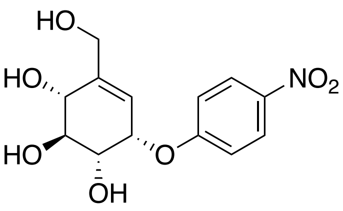

1’

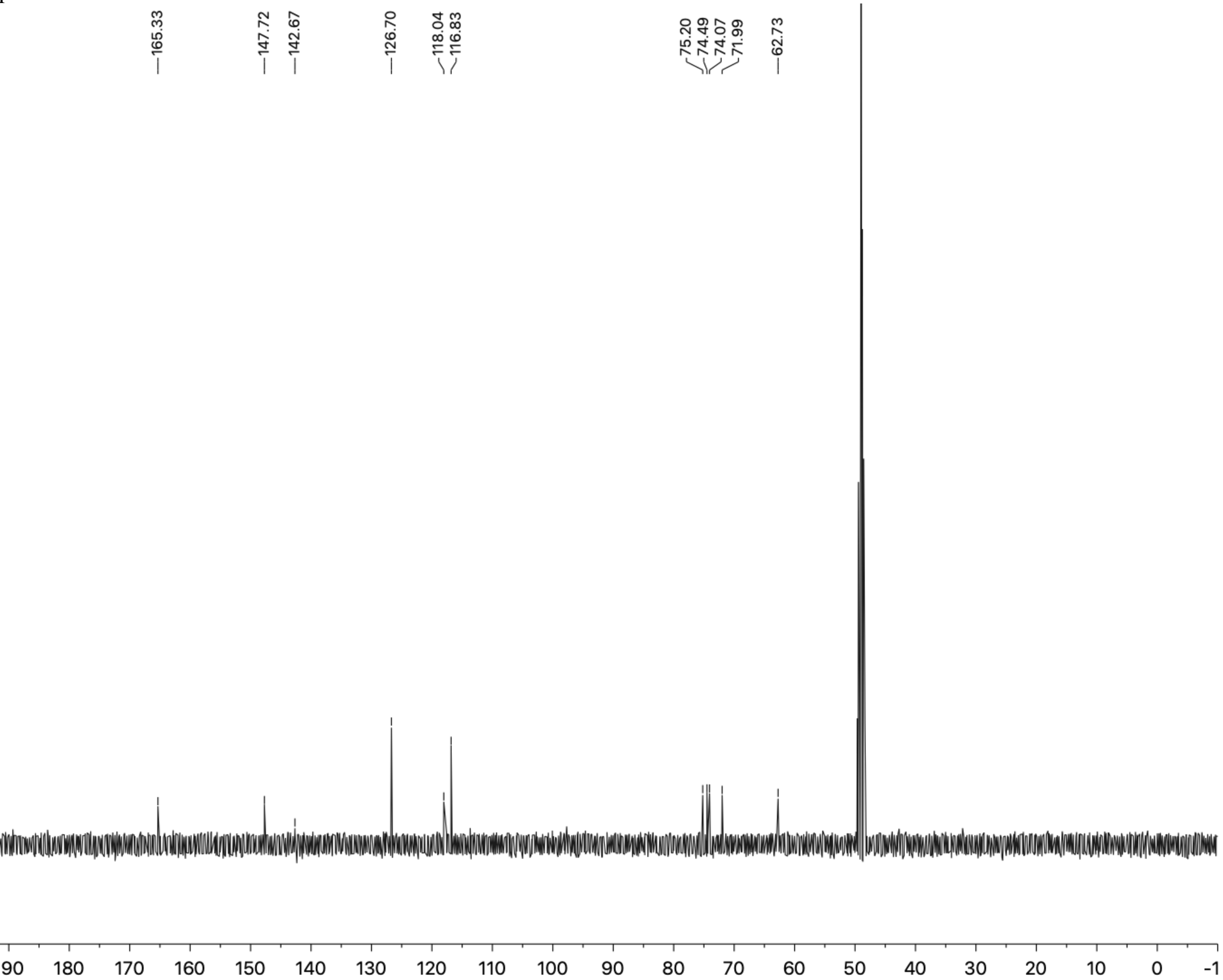

Synthesis of inhibitors **2** and **3**

^{1}H NMR spectrum of compound **7**

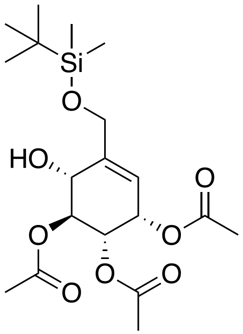

7

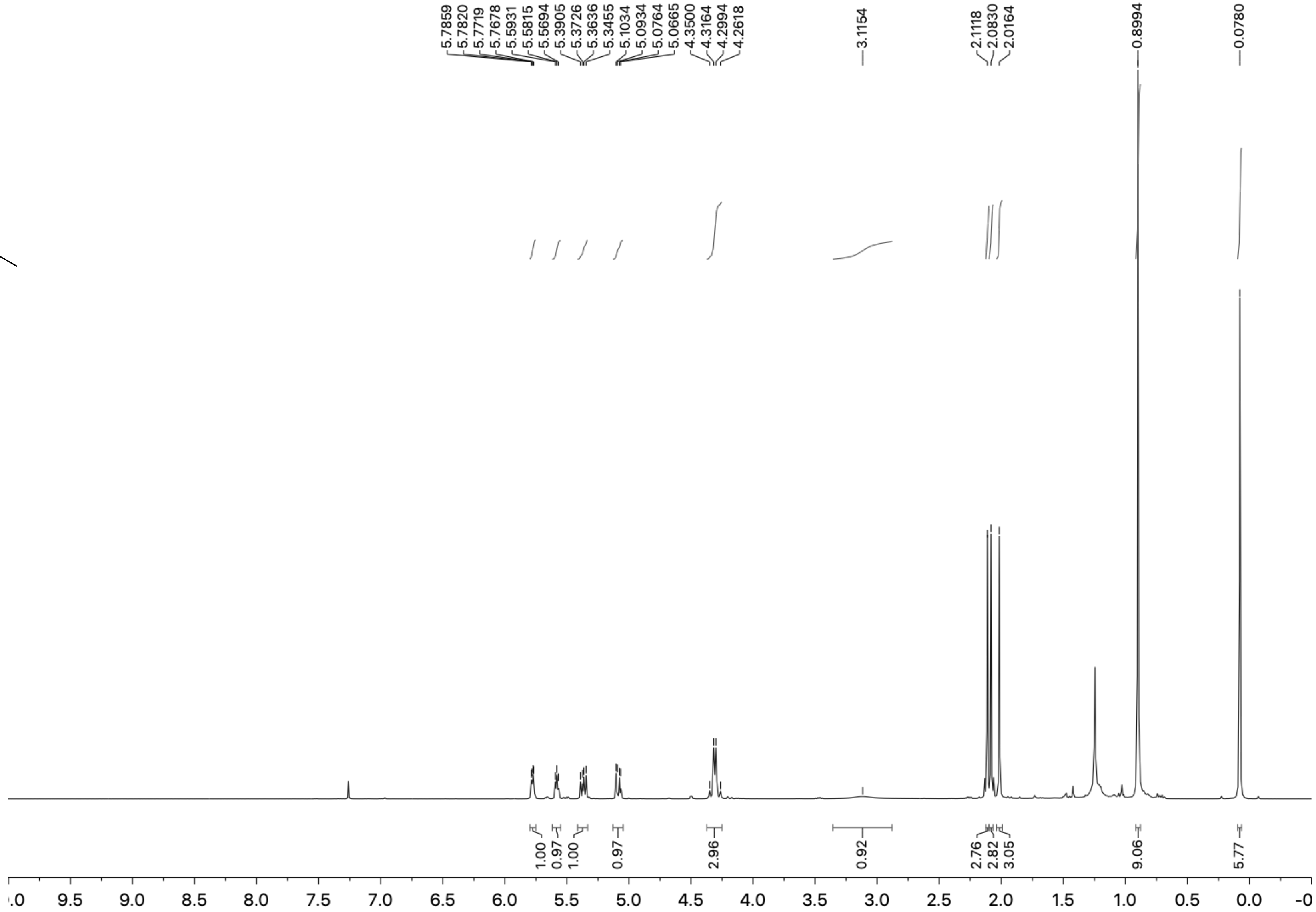

[13]C Spectrum of compound **7**

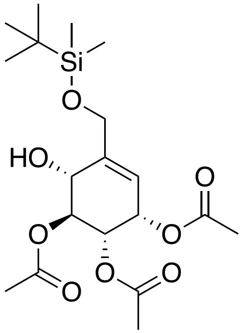

7

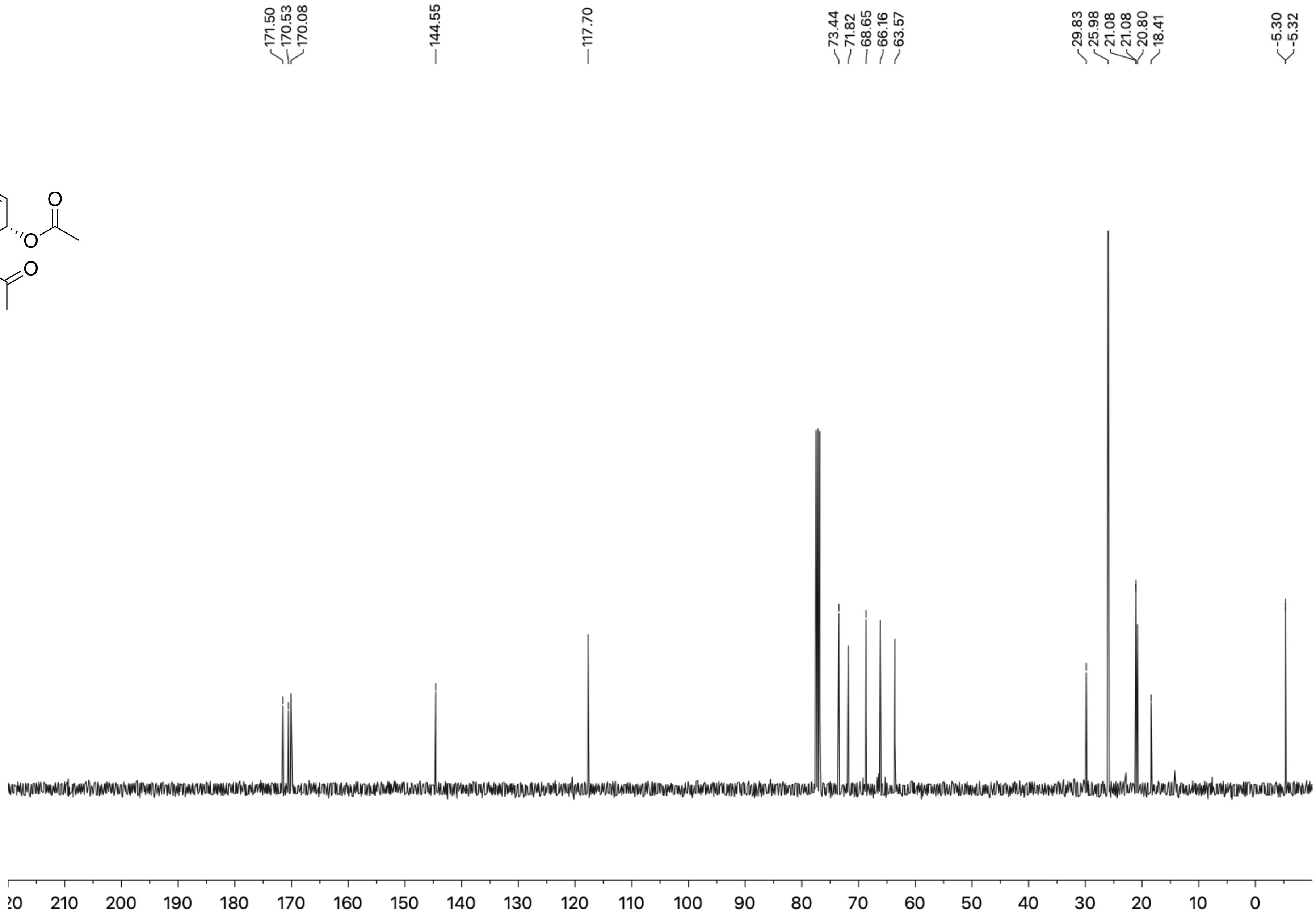

^{1}H NMR spectrum of compound **8**

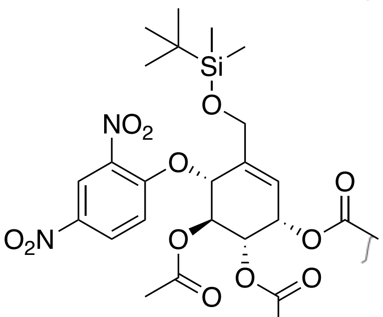

8

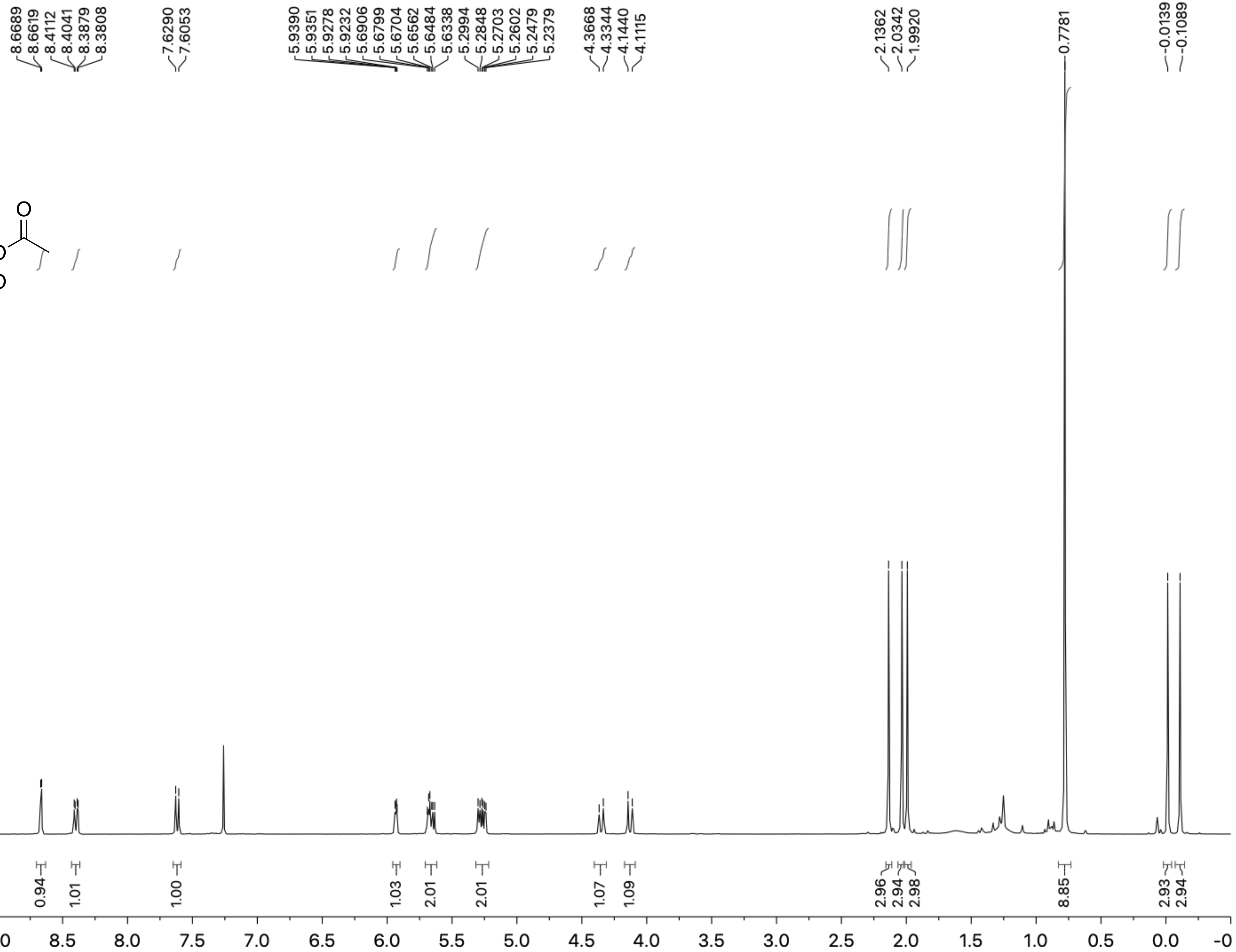

^{13}C Spectrum of compound **8**

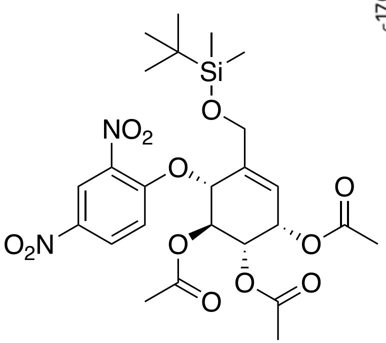

8

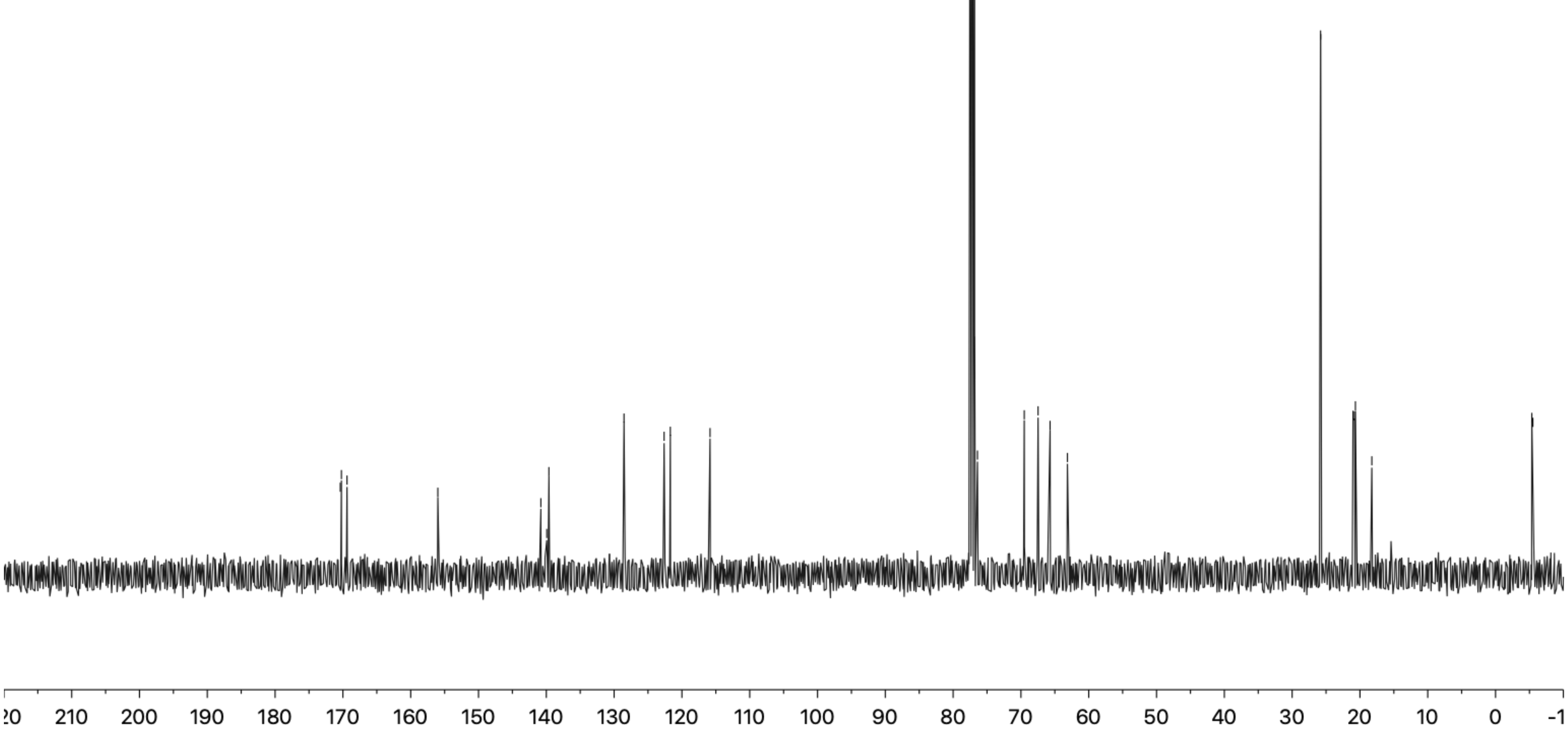

^{1}H NMR spectrum of compound **2**

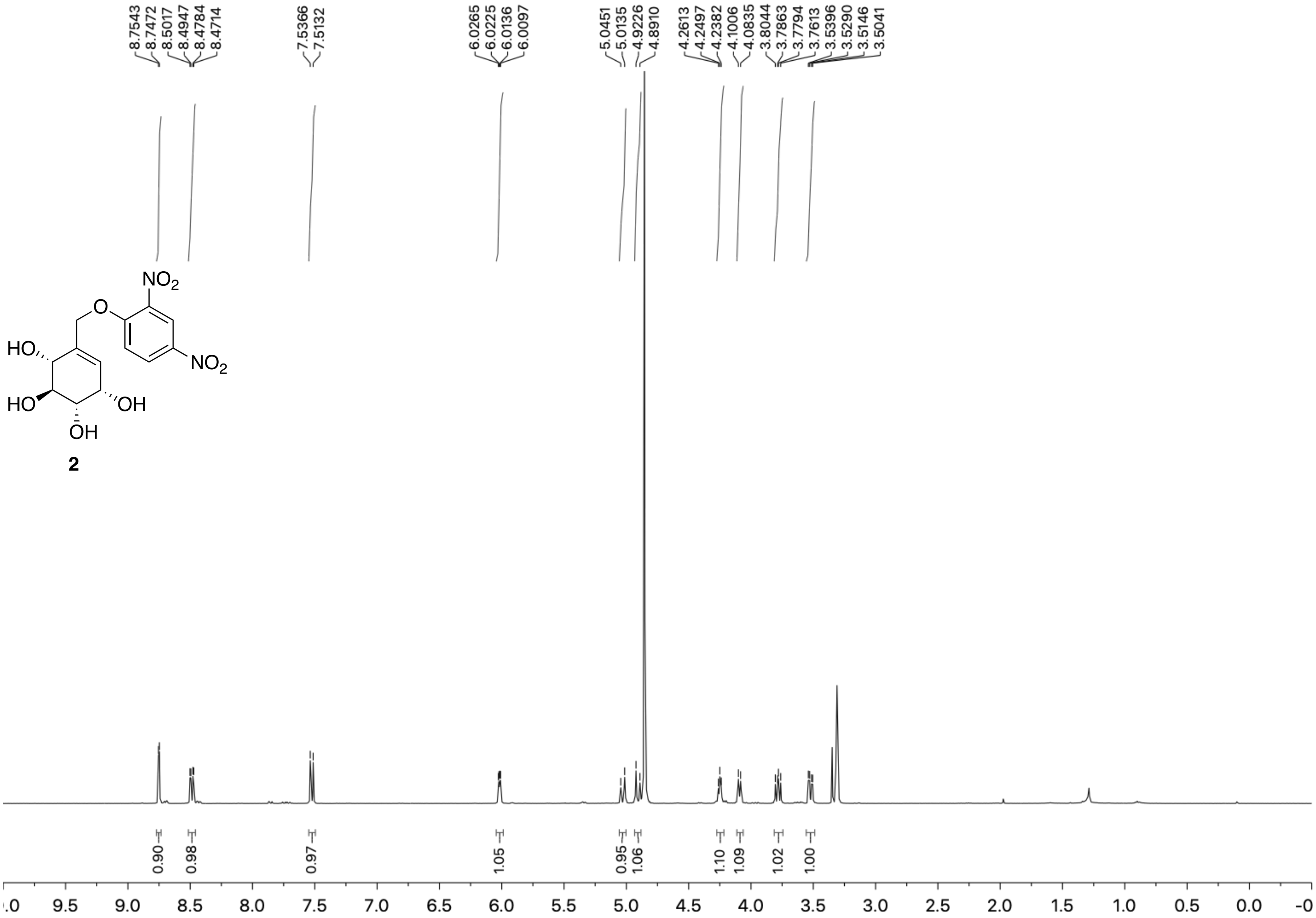

^{13}C NMR spectrum of compound **2**

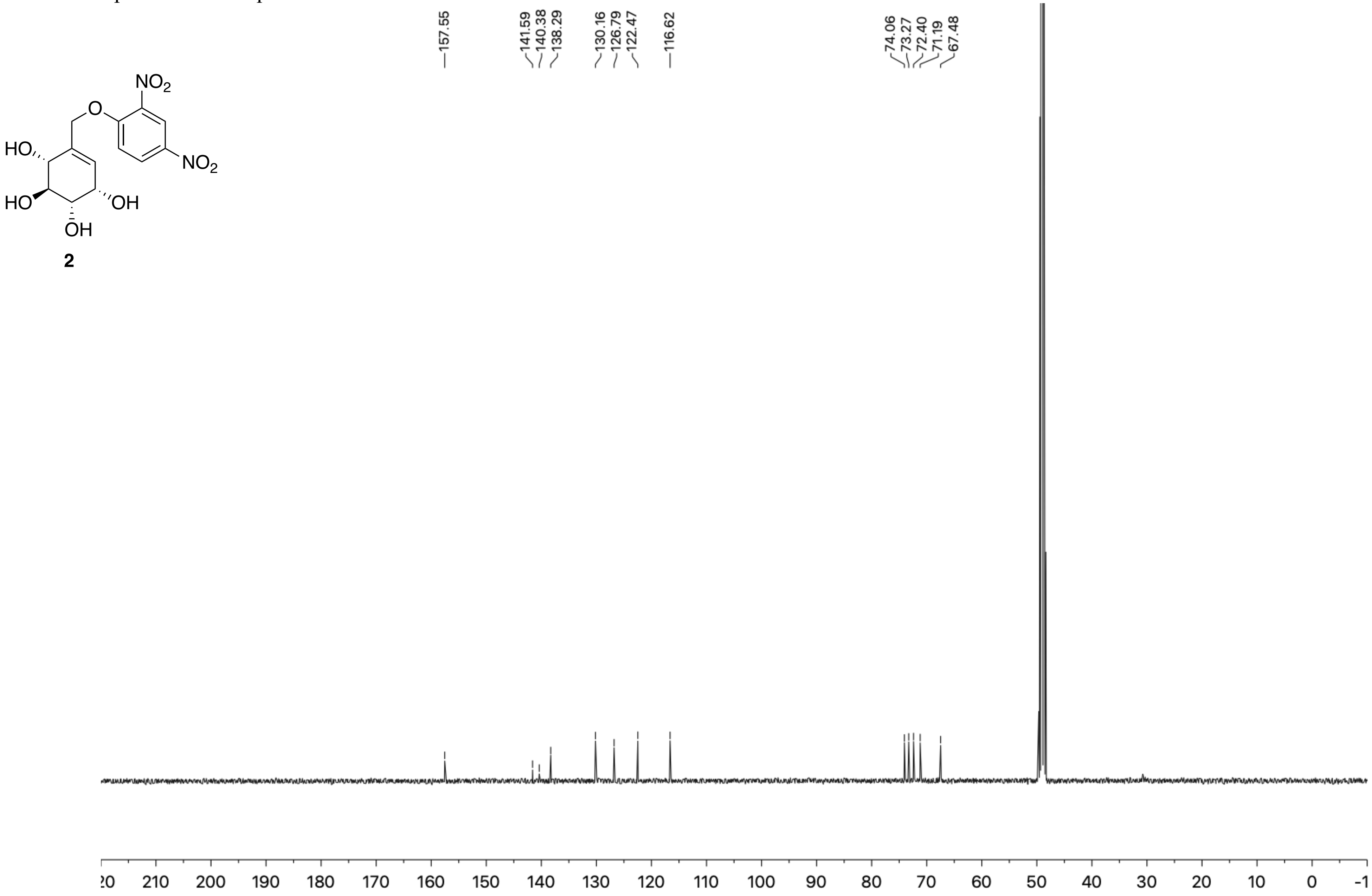

^{1}H NMR spectrum of compound **3**

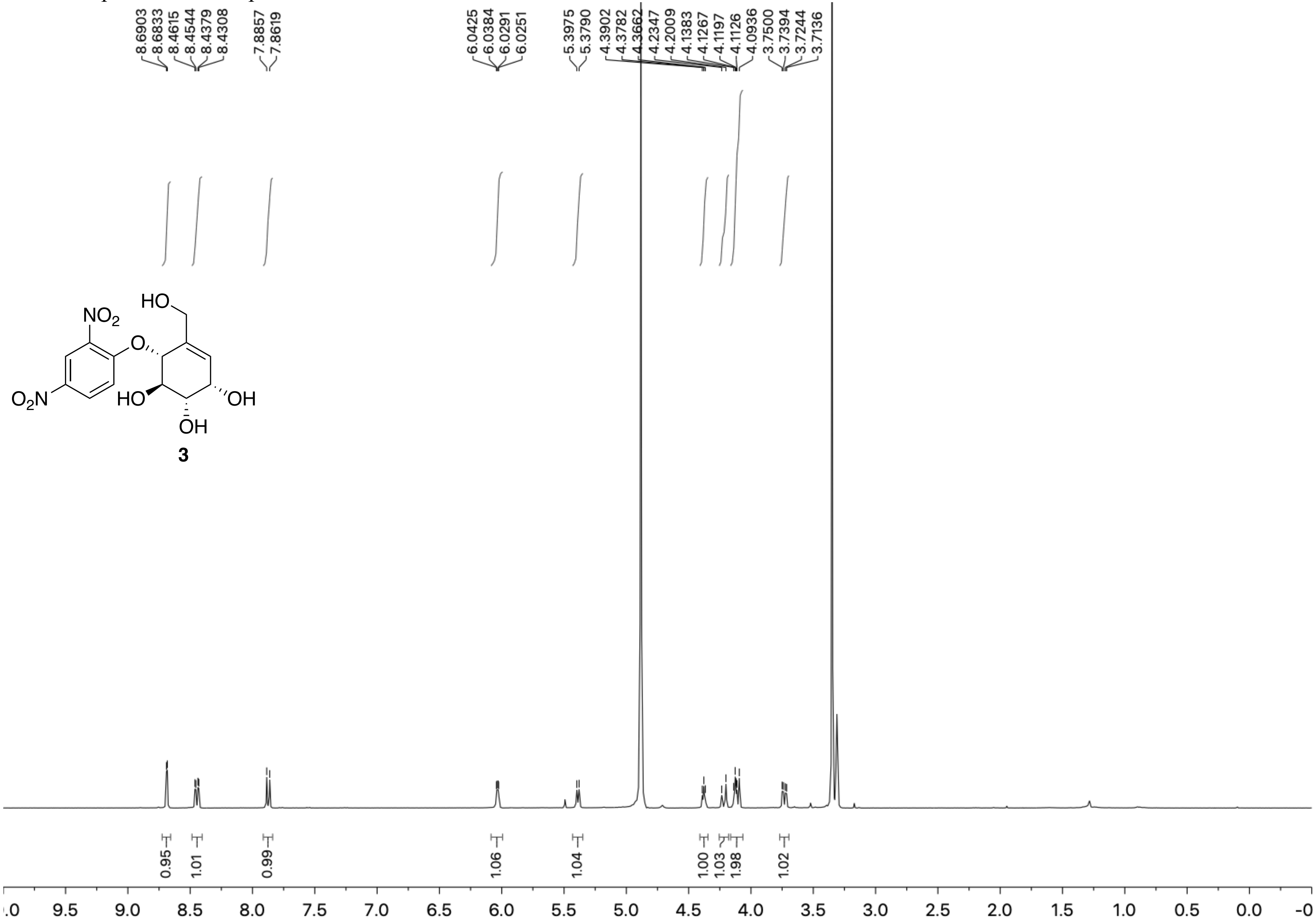

^{13}C NMR spectrum of compound **3**

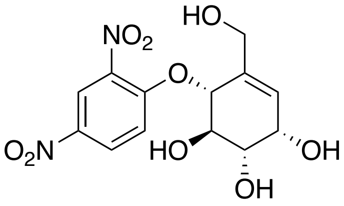

3

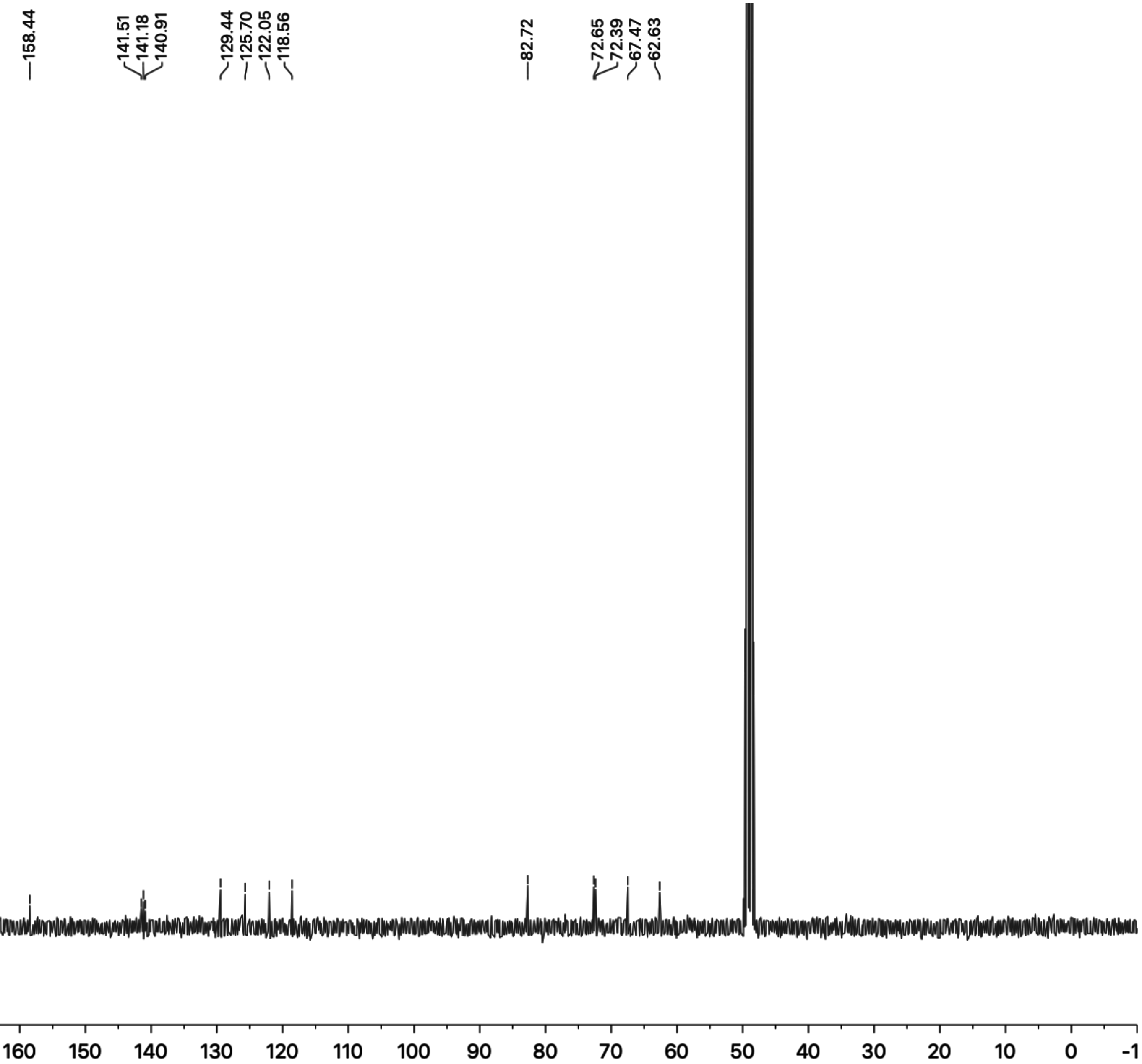

^{1}H NMR Spectrum of compound **10**

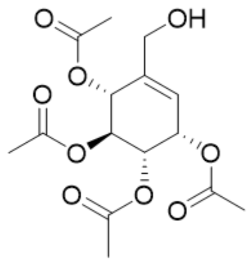

10

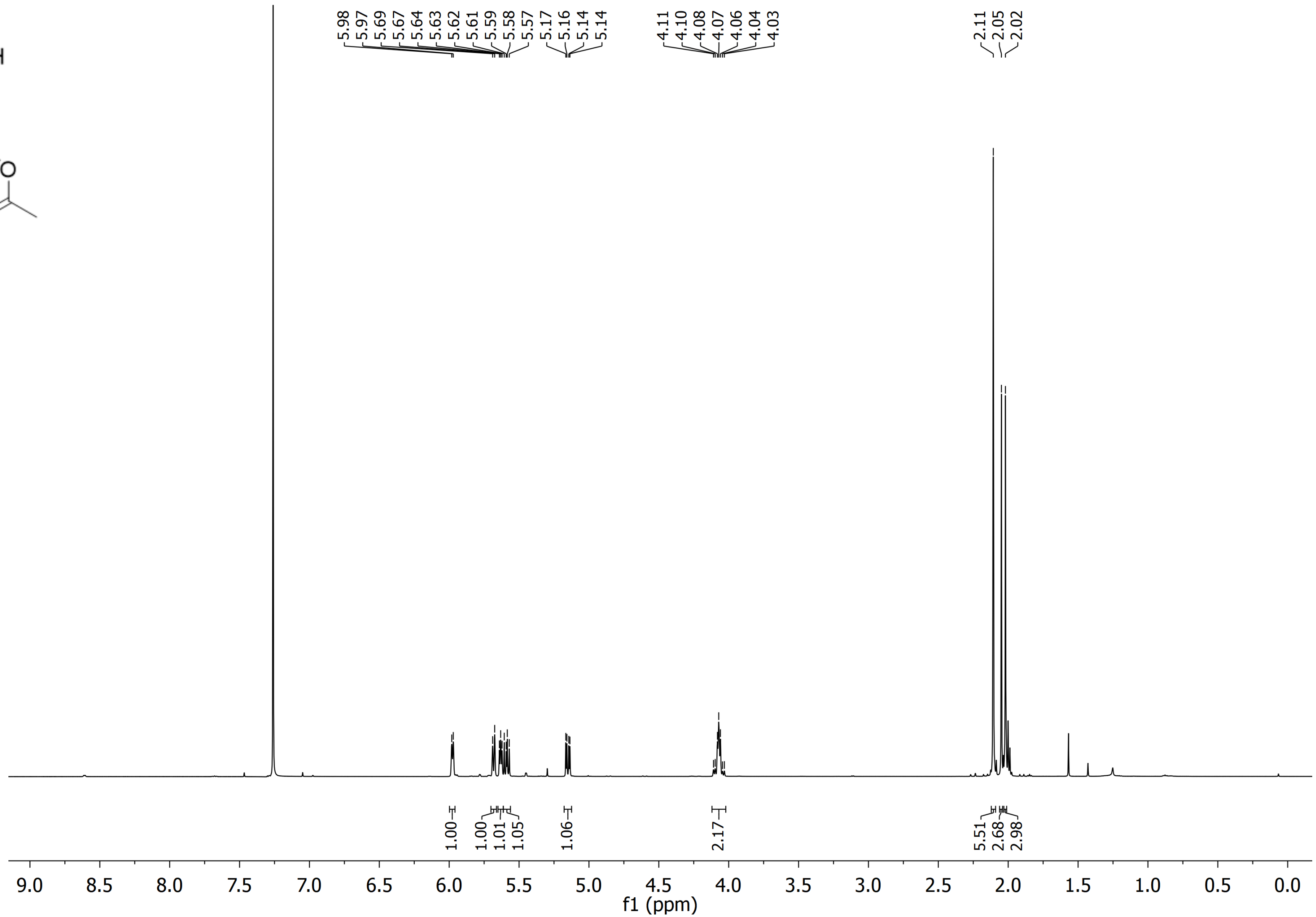

^{13}C NMR Spectrum of compound **10**

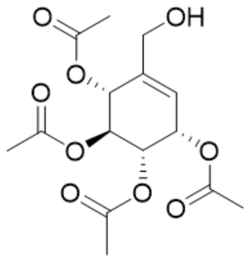

10

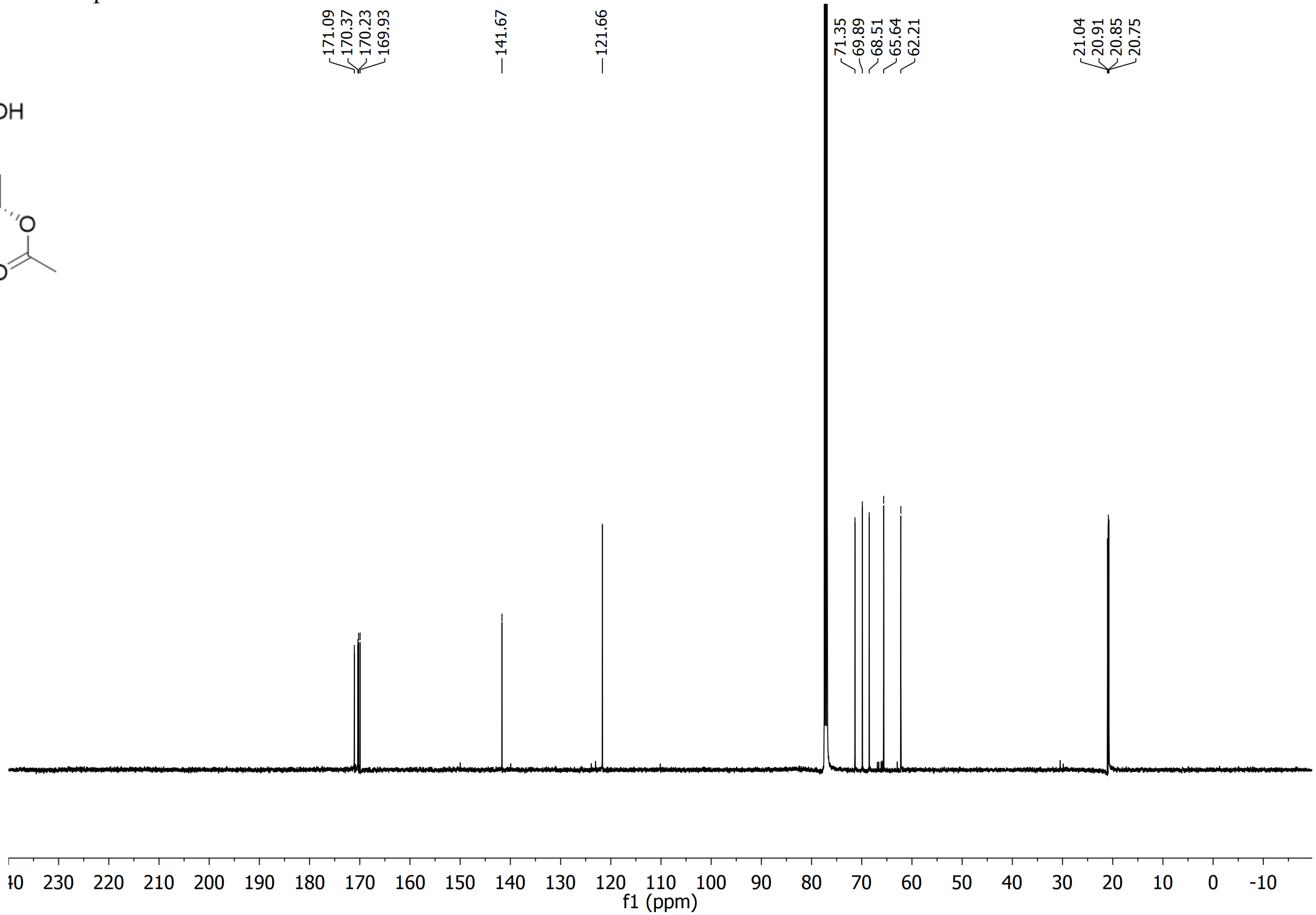

^{1}H NMR Spectrum of compound **11**

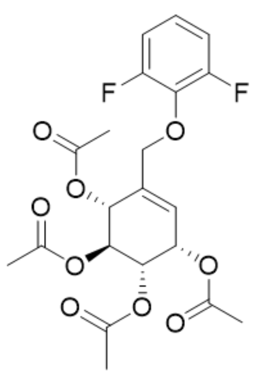

11

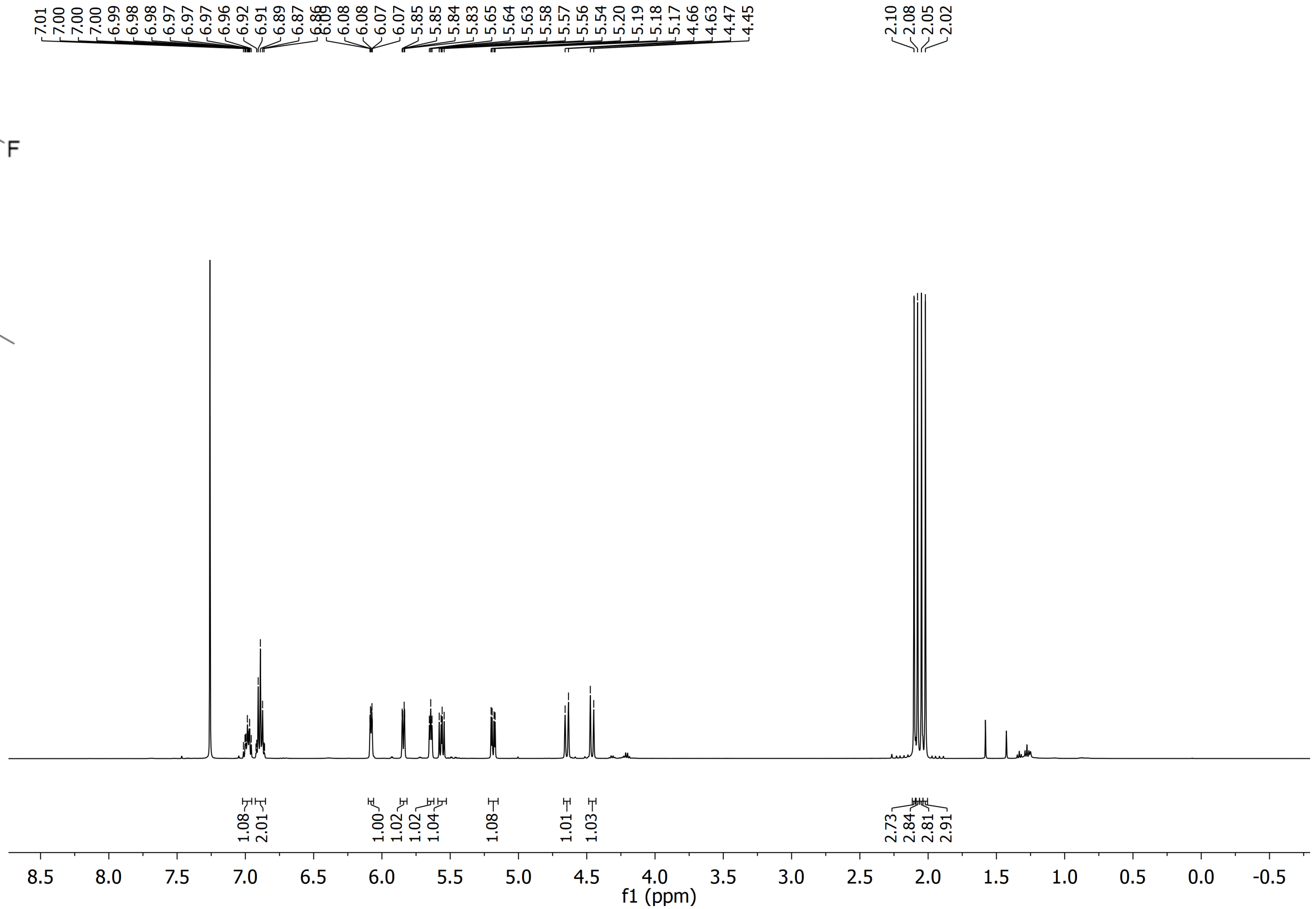

^{13}C NMR Spectrum of compound **11**

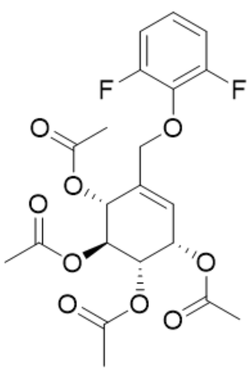

11

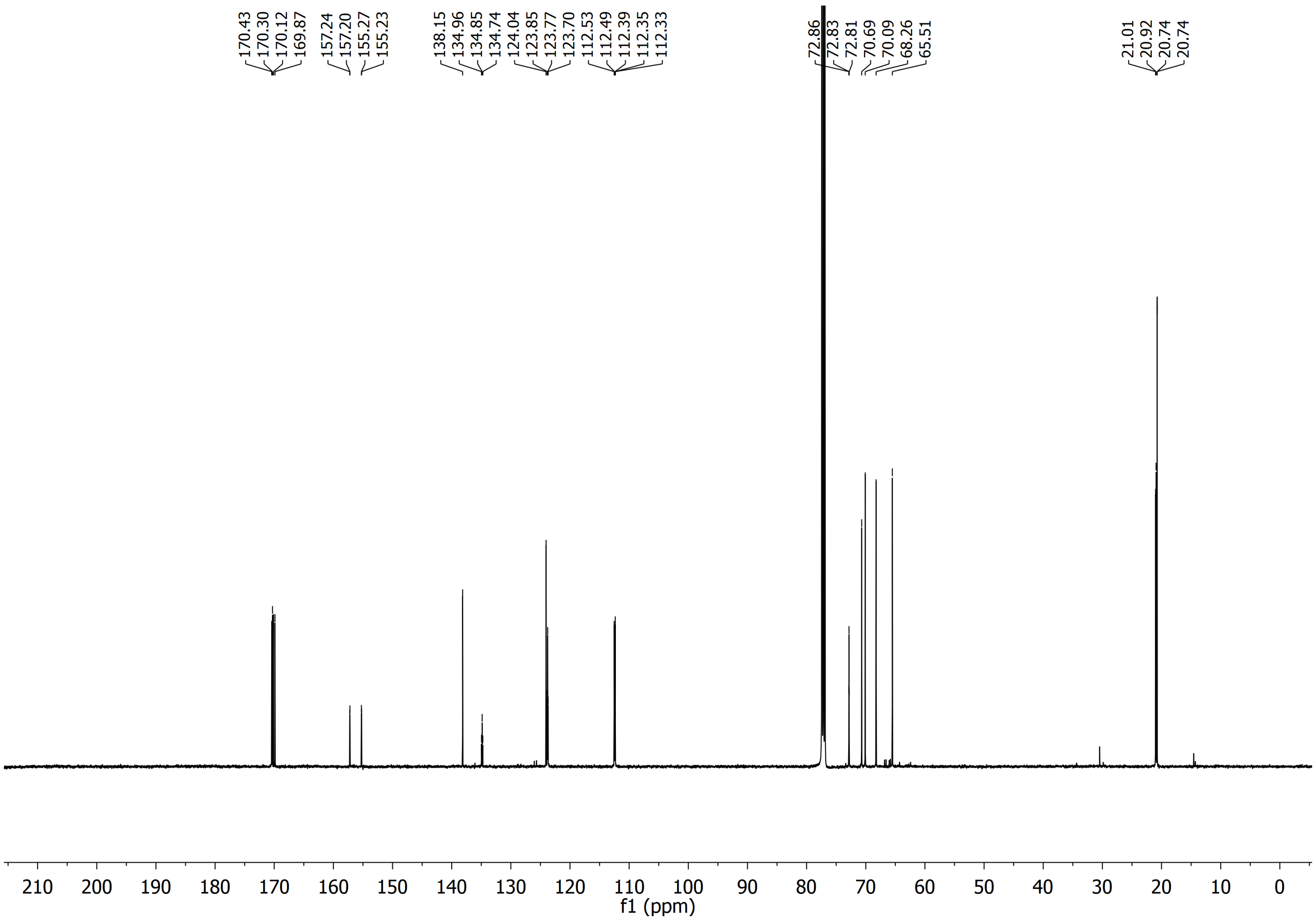

^{19}F NMR Spectrum of compound **11**

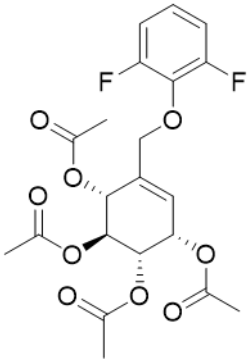

11

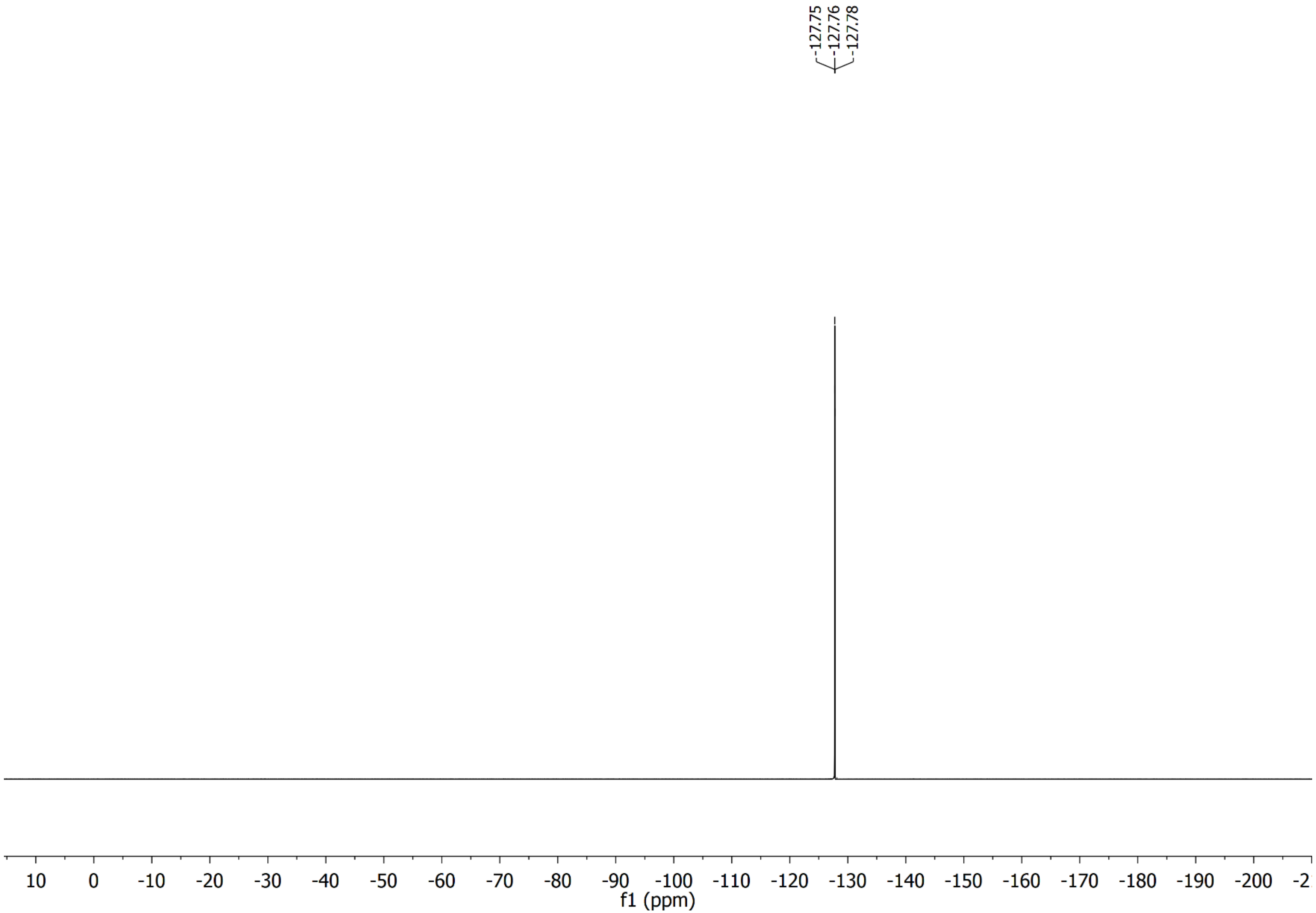

^{1}H NMR Spectrum of compound **9**

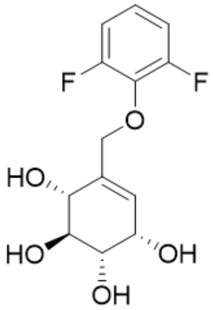

9

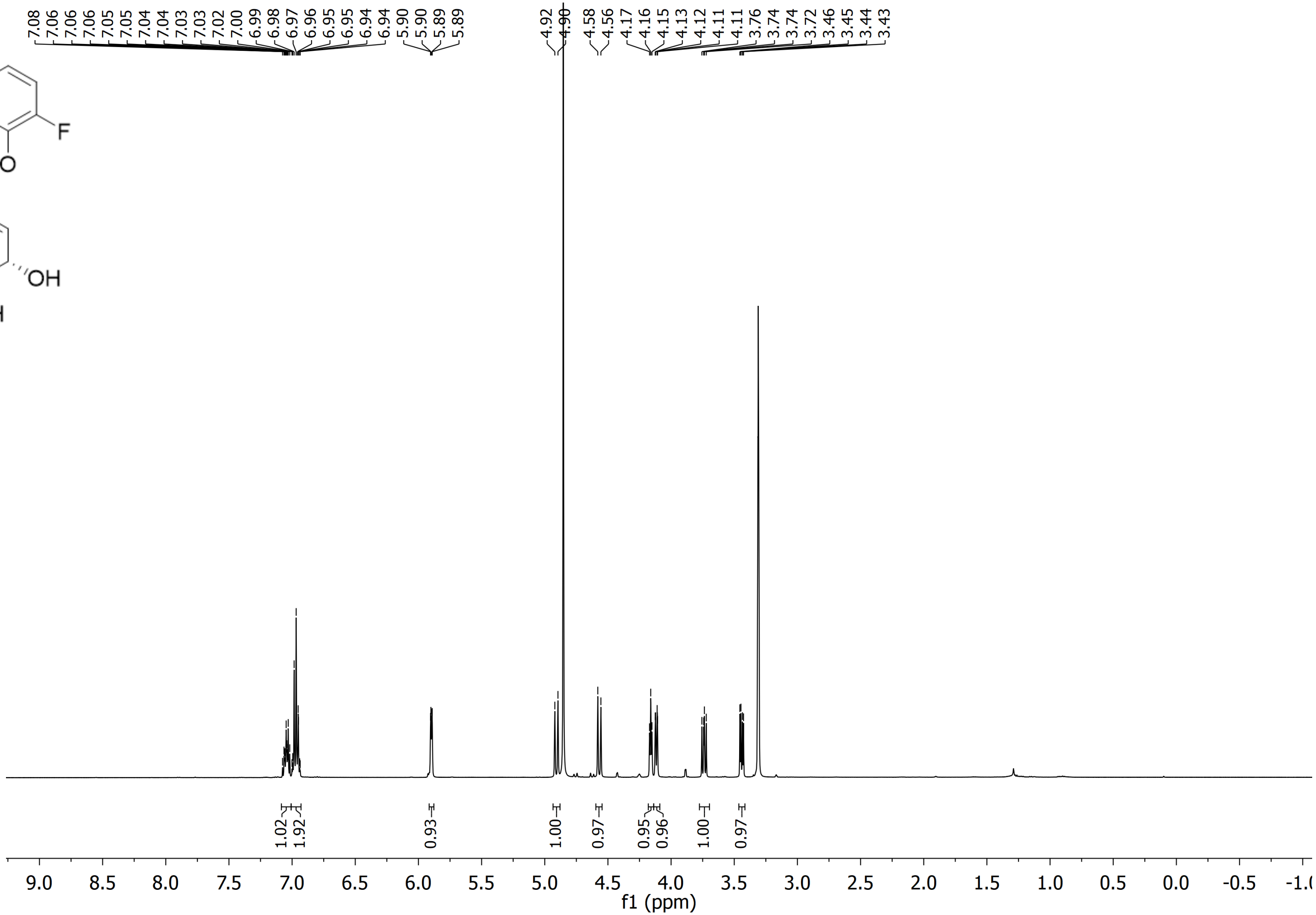

^{13}C NMR Spectrum of compound **9**

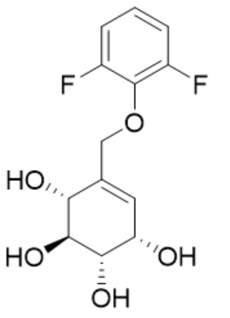

9

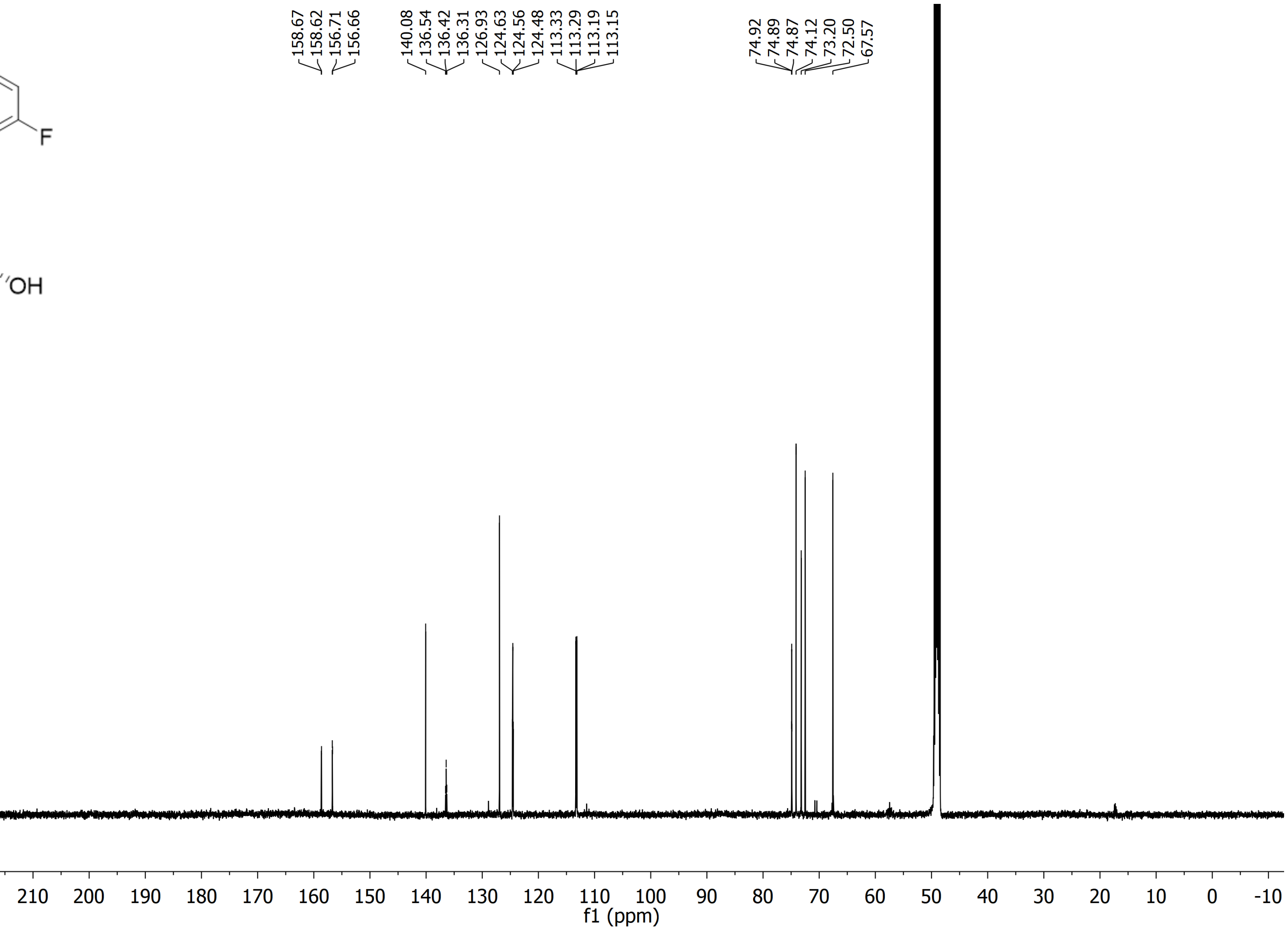

^{19}F NMR Spectrum of compound **9**

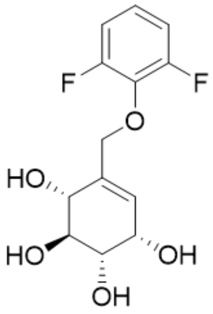

9

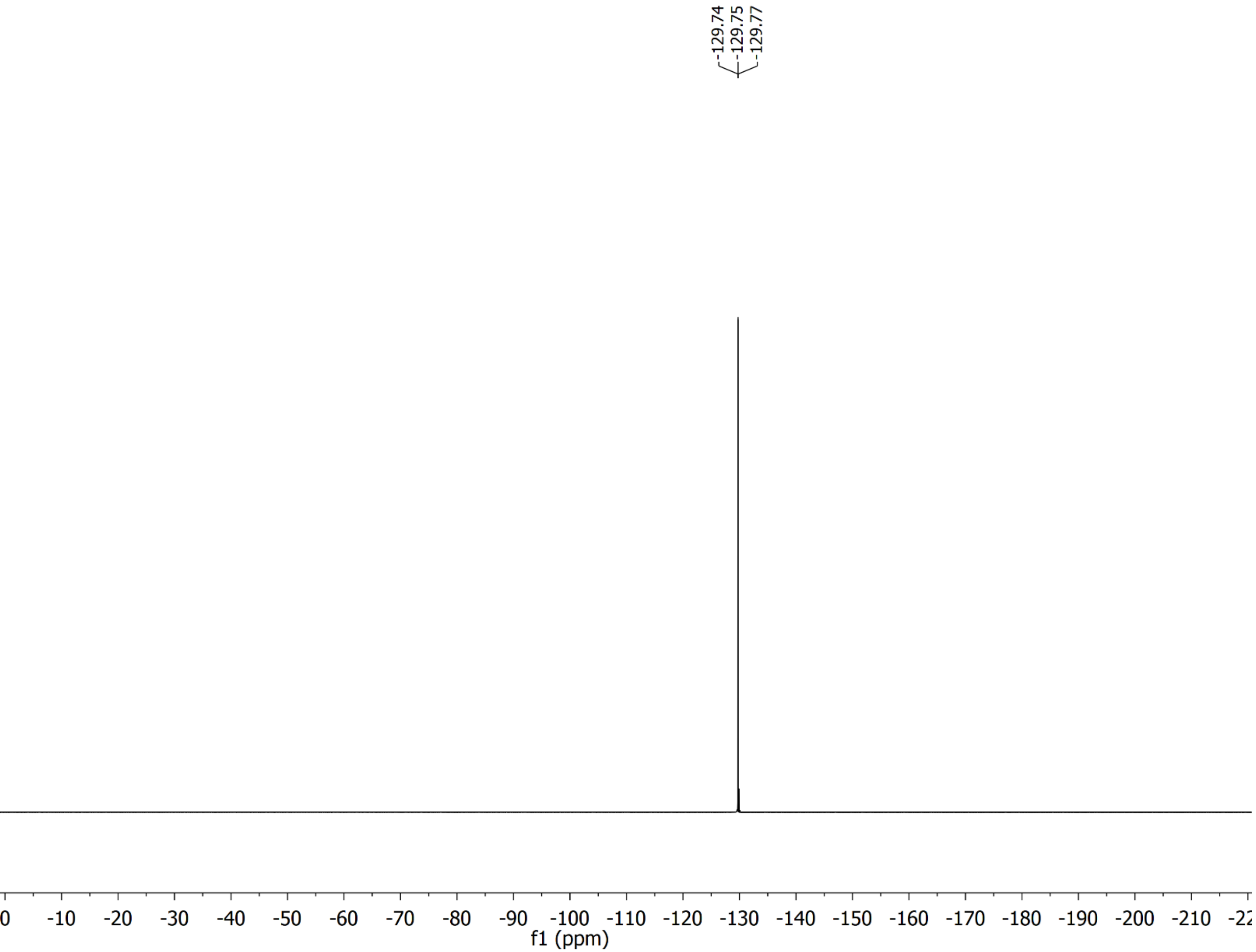

^{1}H NMR Spectrum of compound **11’**

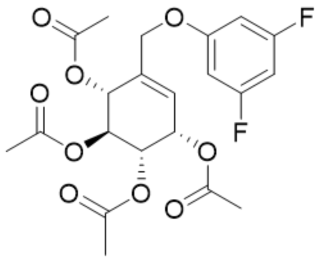

11'

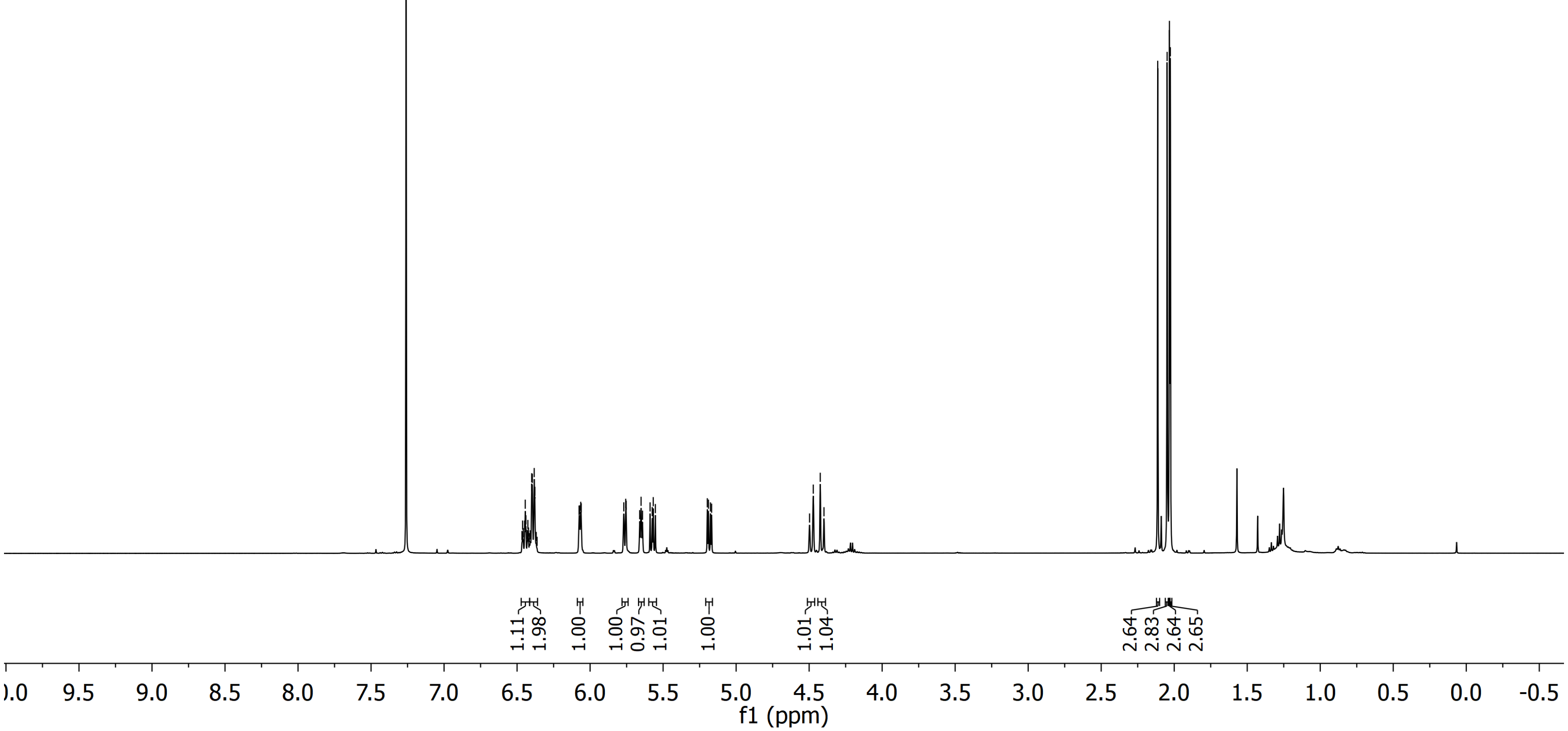

^{13}C NMR Spectrum of compound **11'**

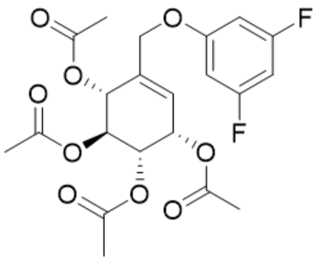

11'

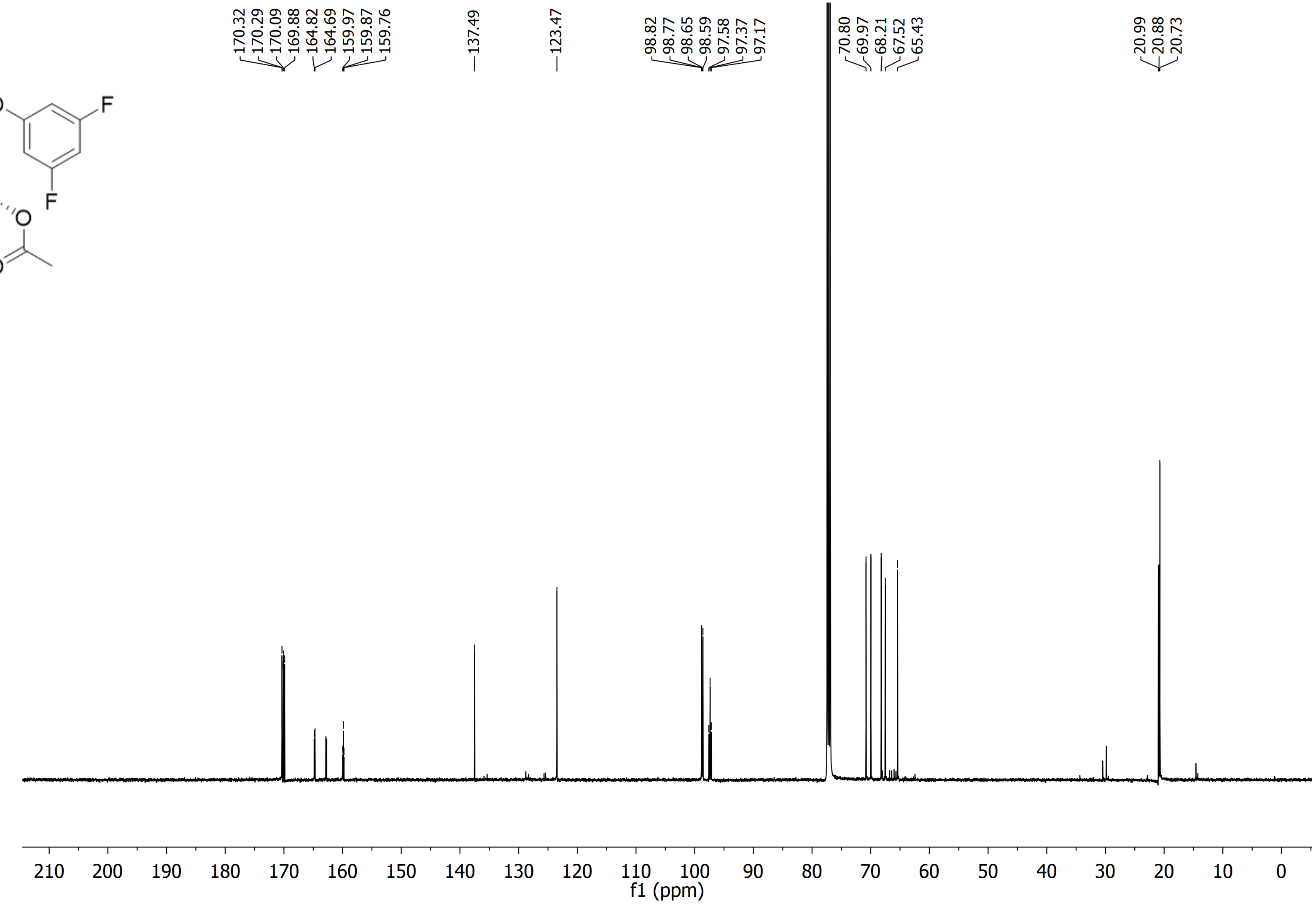

^{19}F NMR Spectrum of compound **11'**

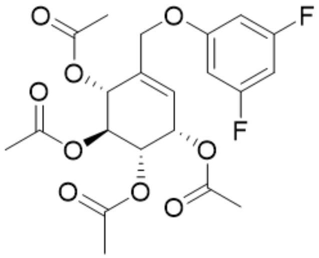

11'

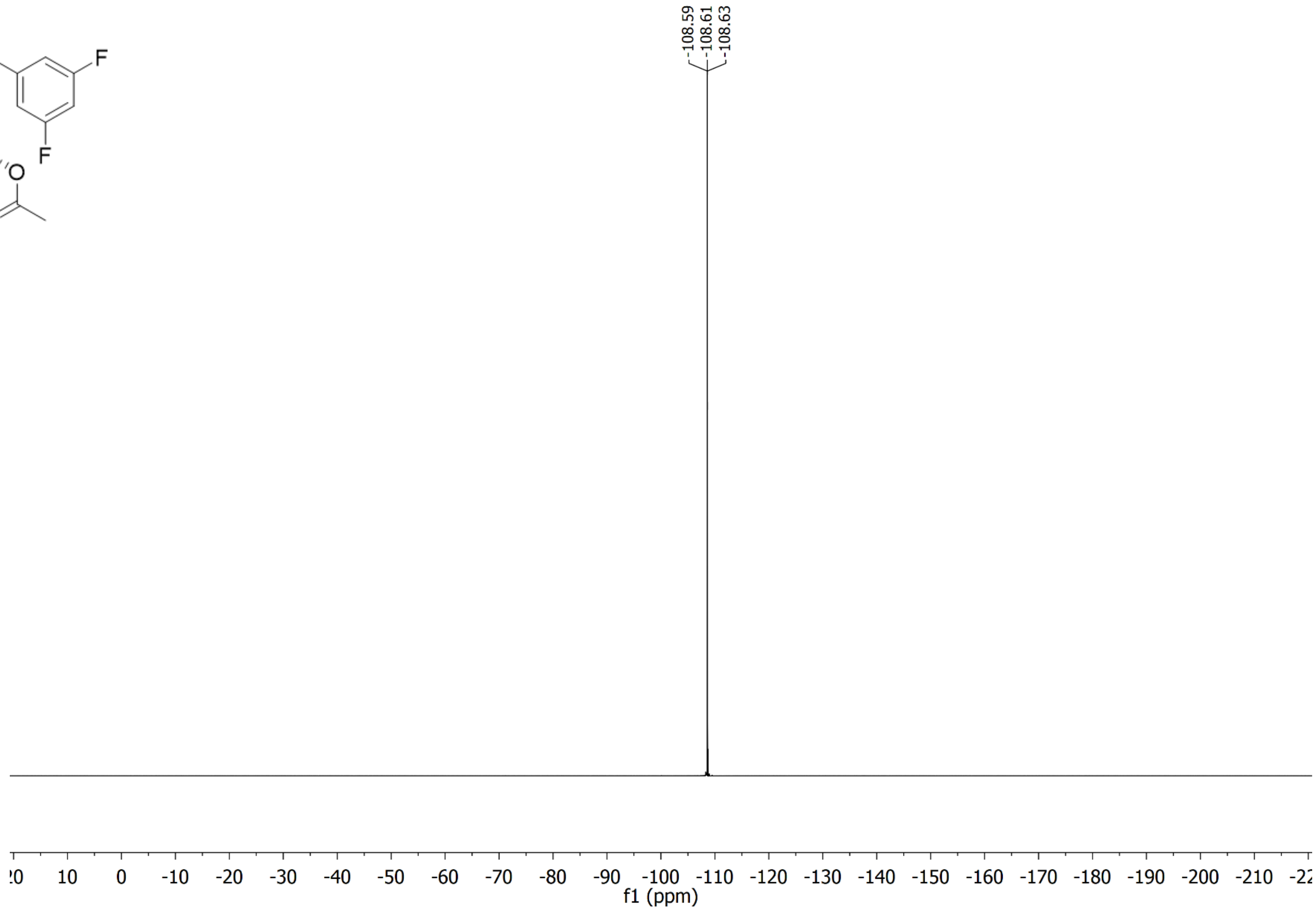

^{1}H NMR Spectrum of compound **9’**

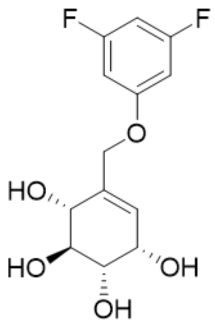

9'

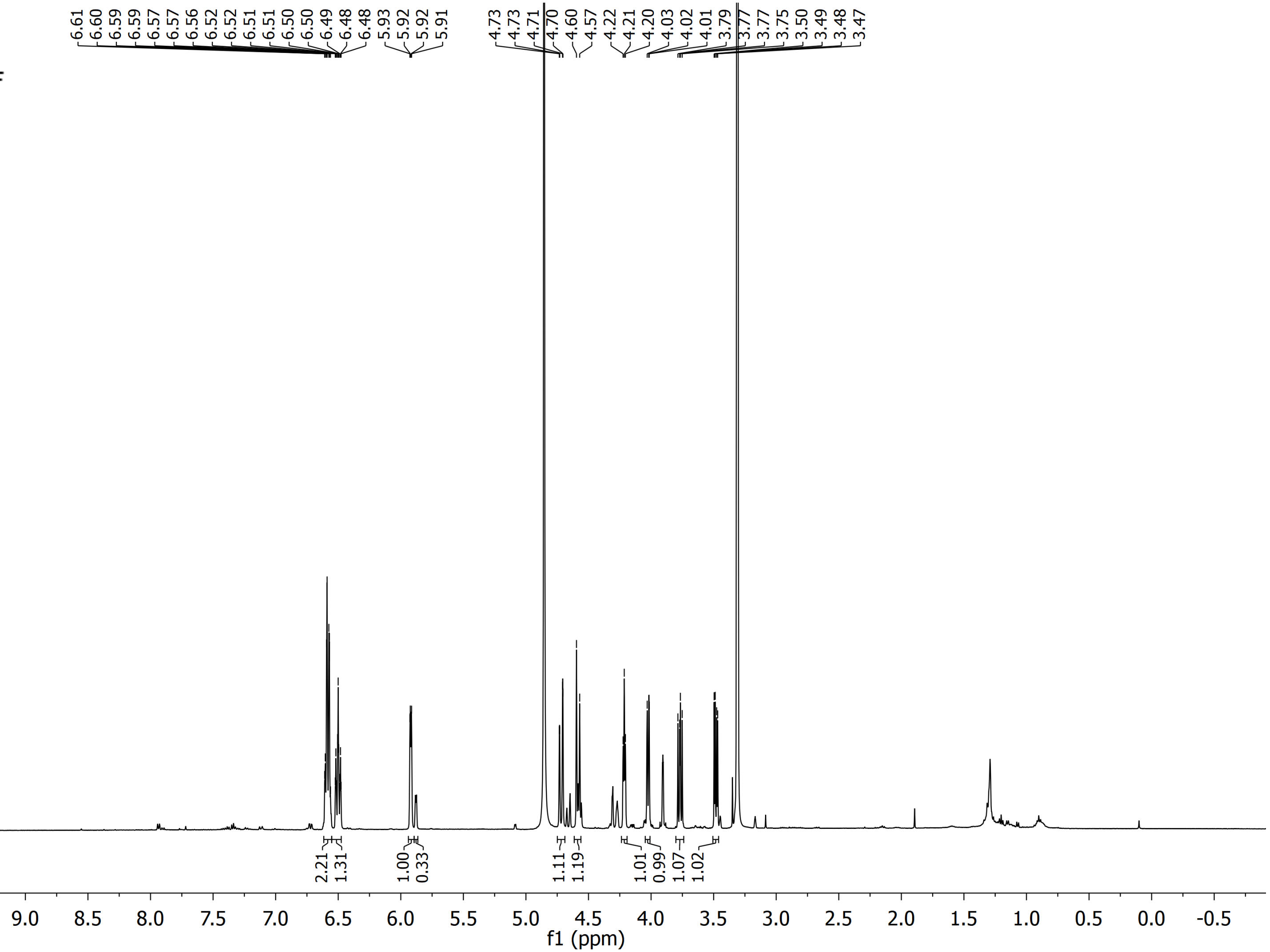

^{13}C NMR Spectrum of compound **9'**

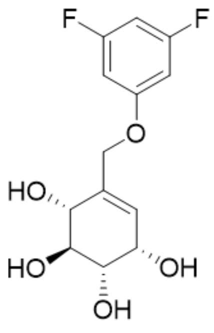

9'

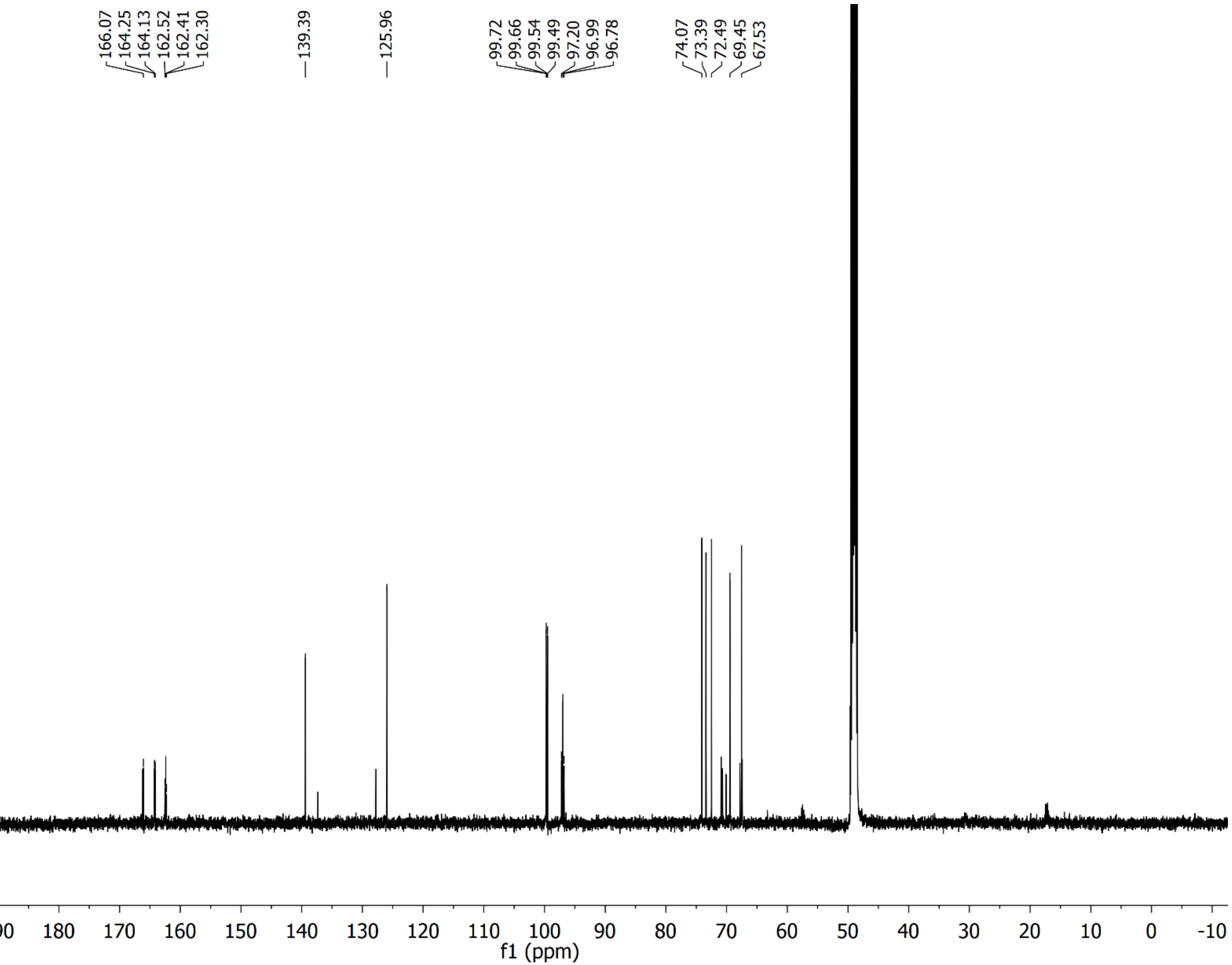

^{19}F NMR Spectrum of compound **9’**

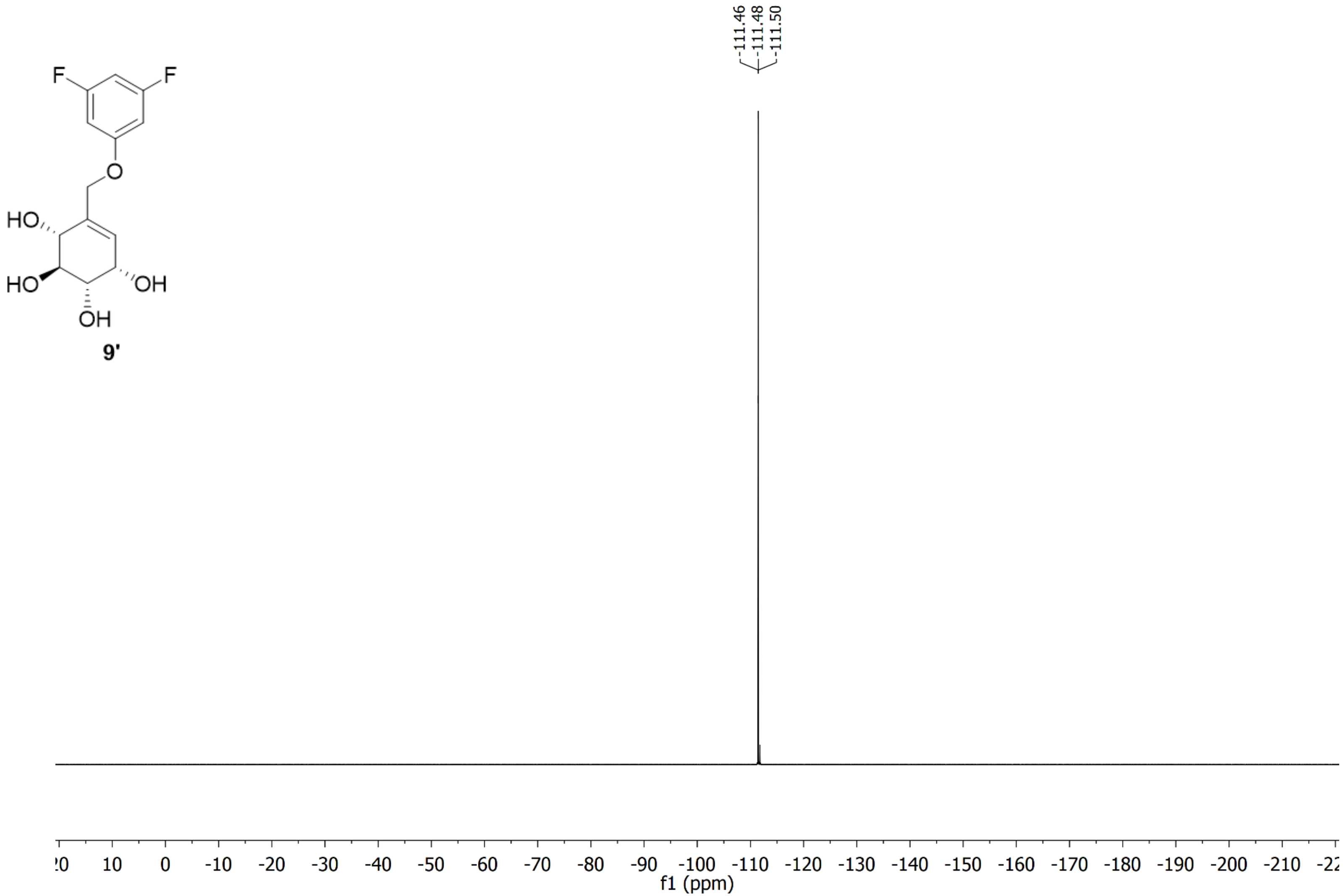

Supplement: Supplementary file 1 — au3c00037_si_001.pdf [file au3c00037_si_001.pdf]
